# Supplementary material for: The membrane insertion of the pro-apoptotic protein Bax is a Tom22-dependent multi-step process: a study in nanodiscs
Source: Cell Death Discov. 2024 Jul 23;10:335. doi: 10.1038/s41420-024-02108-x (PMC11266675; doi:10.1038/s41420-024-02108-x)
Supplement: Supplementary file 1 — supplementary data [file 41420_2024_2108_MOESM1_ESM.pdf]

Supplementary data

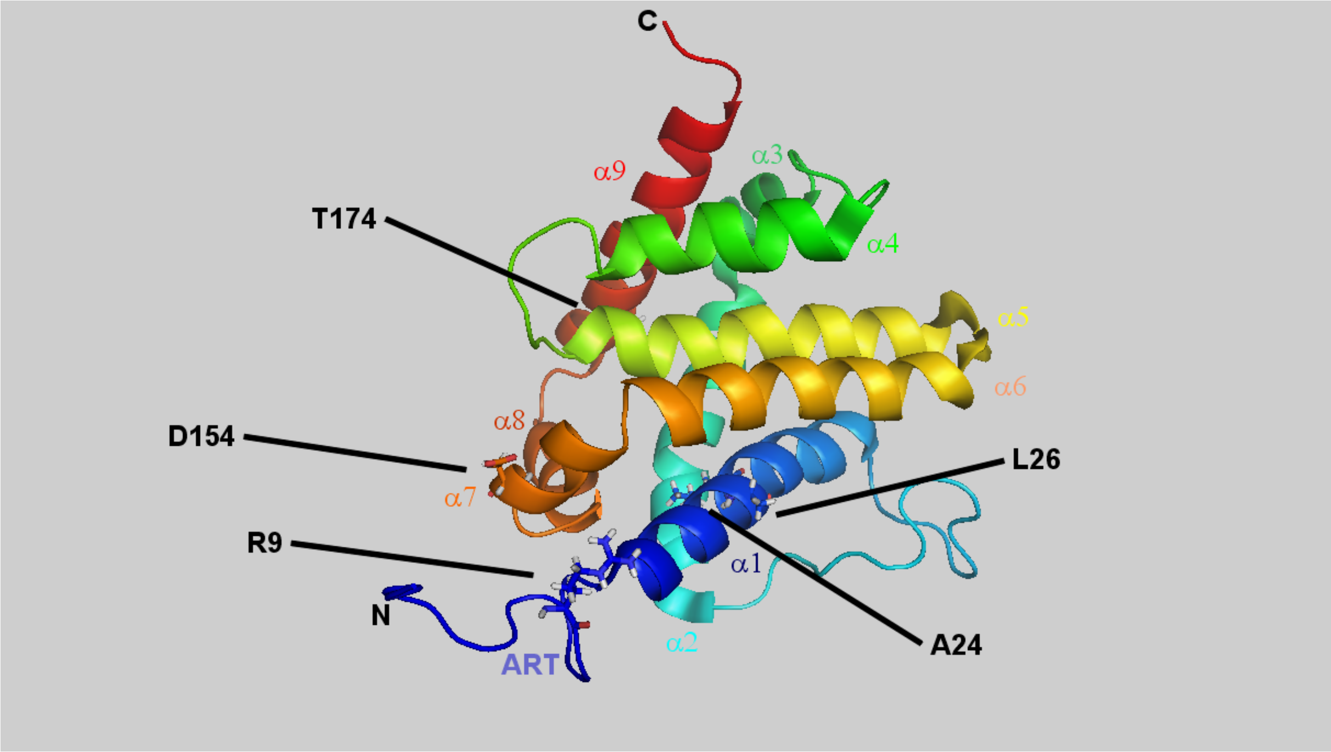

Figure S1: Structure of soluble inactive Bax [3] (pdb 1F16), showing the positions of residues mutated in this study.

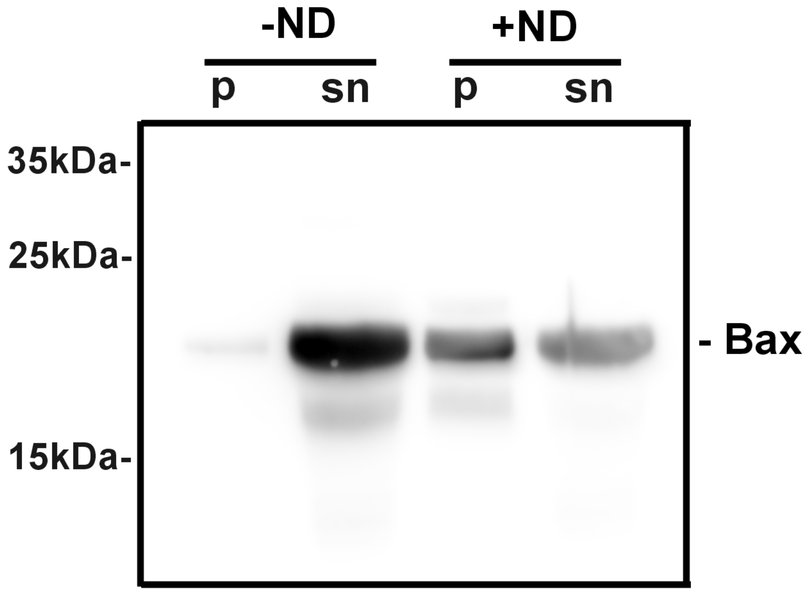

Figure S2. Precipitation of Bax induced by nanodiscs during cell-free synthesis

BaxWT was synthesized in the absence (-ND) or in the presence of 3.5µM nanodiscs (+ND). After the synthesis, the reaction mix was weighted and centrifuged at 20,000 x g for 15 minutes. The pellet was resuspended in the same volume of buffer B (25mM Hepes/Na pH 7.4, 200mM NaCl, 0.1mM EDTA). Aliquots from both the pellet (p) and supernatant (sn) were mixed with an equal volume of Laemmli buffer 2X, and analyzed by SDS-PAGE and western blot against Bax. Blot is representative of at least 10 independent experiments.

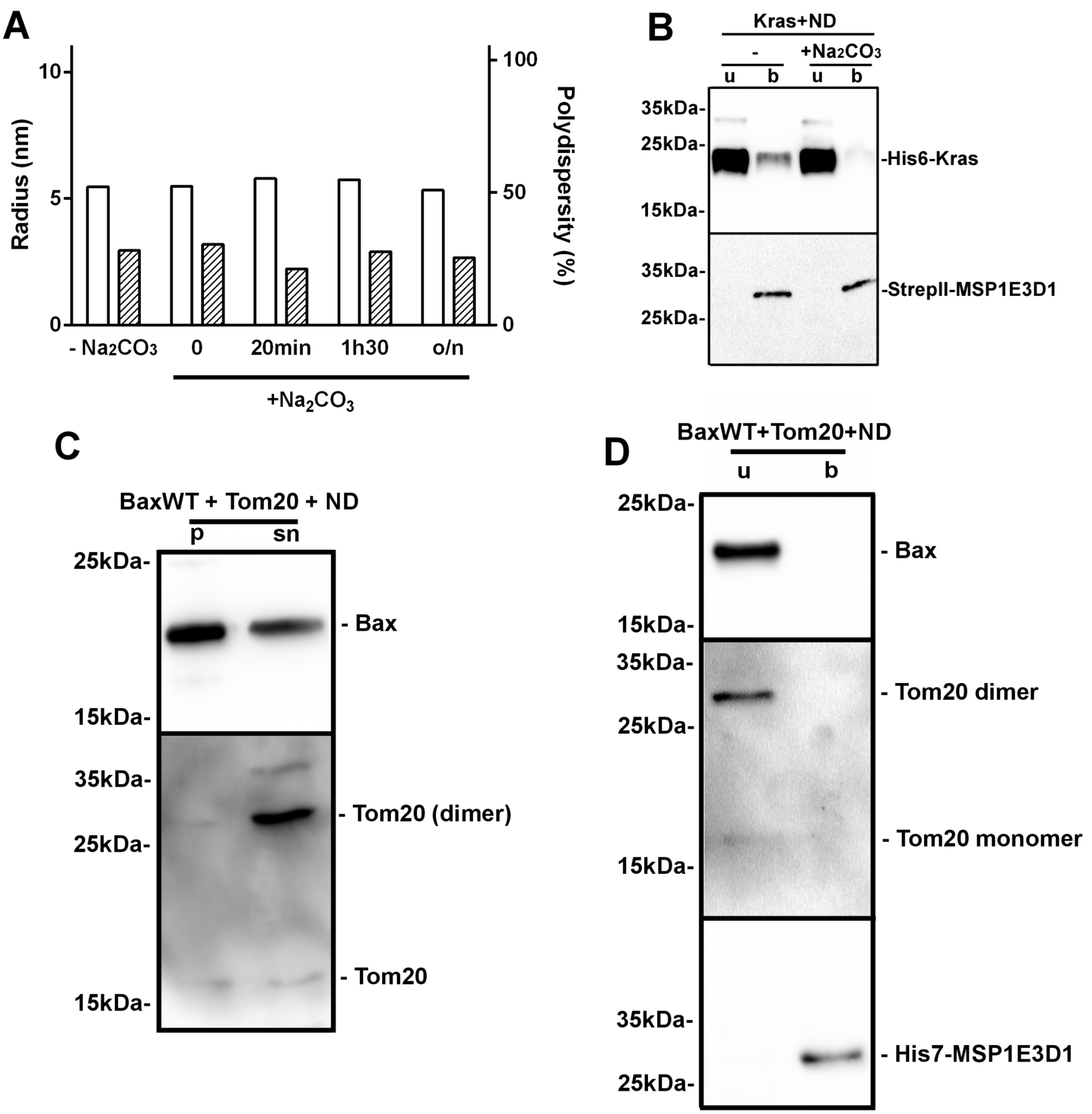

Figure S3. Controls of the effect of alkaline Sodium Carbonate treatment on nanodiscs and of the specificity of the effect of Tom22.

(A) Nanodiscs were incubated for the indicated times in the presence of 0.1M Sodium Carbonate (pH 10.0). Radius (white bars) and polydispersity (grey bars) were measured by DLS (3 measurements of 10 acquisitions each), showing that the integrity of nanodiscs was not affected by the treatment.

(B) His6-tagged human Kras4b was synthesized in cell-free in the presence of StrepII-MSP1E3D1 nanodiscs. Nanodiscs were purified on StrepII-Tactin, then incubated for 15 minutes in the absence or in the presence of 0.1M Sodium Carbonate (pH 10.0). They were then re-purified on StrepII-Tactin, and the unbound and bound fractions were analyzed by western-blots. A minor fraction of Kras4b remained bound to nanodiscs, but was removed after alkaline treatment. Blot is representative of 2 independent experiments.

(C,D) Bax was synthesized alone or co-synthesized with Tom20 and analyzed as in Fig.1A and 1C showing that Tom20 did not prevent Bax precipitation and did not promote its insertion. Blots are representative of 2 independent experiments.

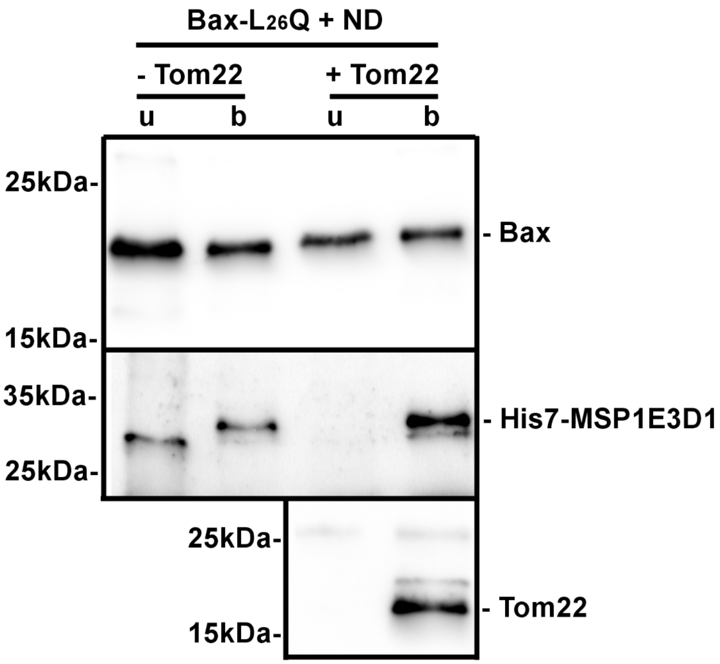

Figure S4: The mutation L^{26}Q in the GALLL motif partially abolished Tom22-induced Bax insertion.

Same experiment as in Fig.2H with BaxL^{26}Q instead of BaxA^{24}R. Blot is representative of 2 independent experiments.

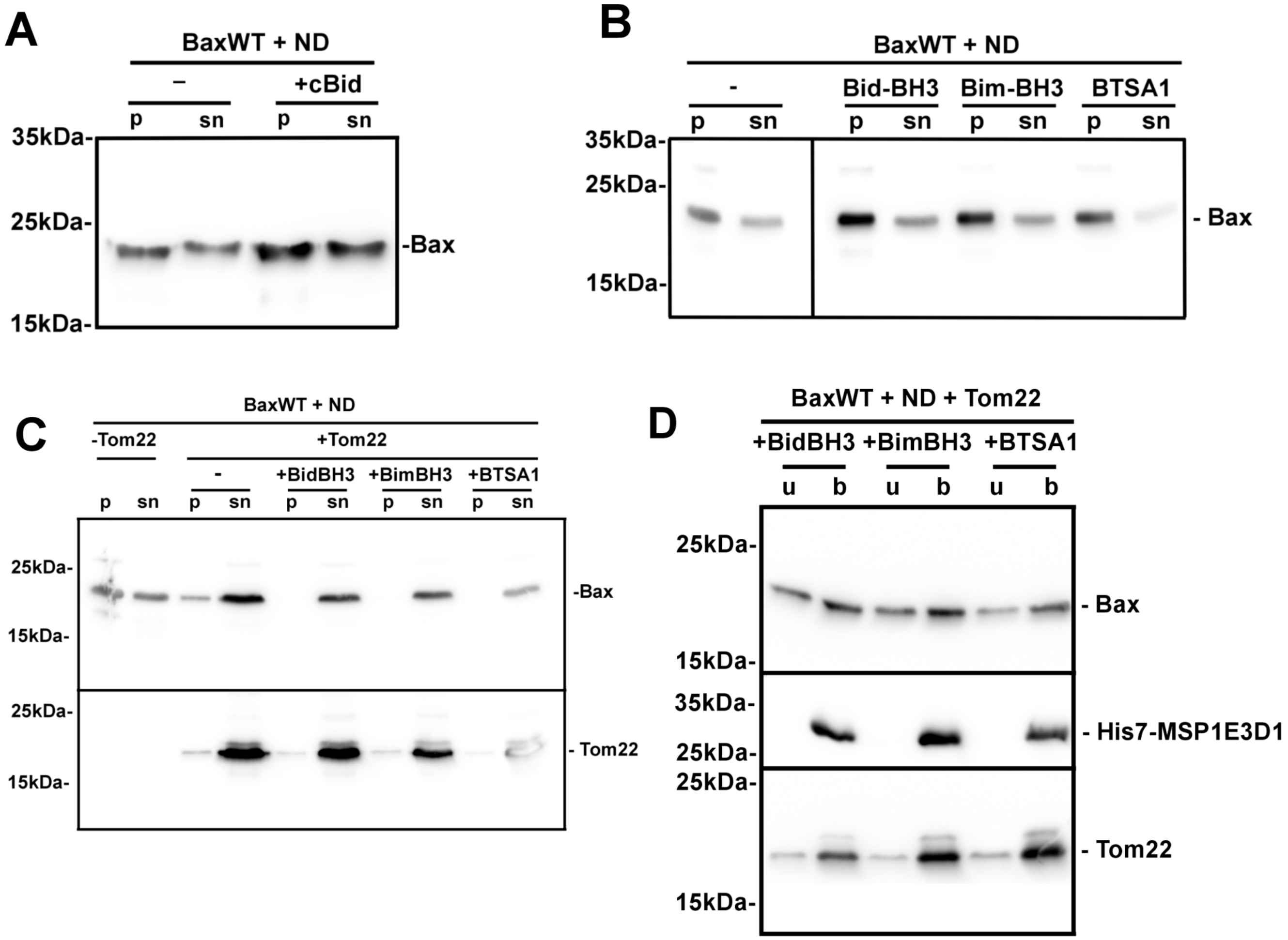

Figure S5. BH3-activators did not prevent Bax precipitation in the presence of nanodiscs

(A) Same experiments as in Fig.1A (without Tom22), except that 10µg caspase-8-cleaved Bid (cBid) was included in the reaction mix. We determined that the maximal concentration of Bax produced in the cell-free system was 0.5 mg/mL, *i.e.* 50µg of Bax in the 100µL-reaction mix. We therefore set up a cBid to Bax ratio of ~1 to 5 (considering that the sizes of the two proteins are close to each other) [70]. Blot is representative of 2 independent experiments.

(B) Same experiment as in Fig.1A (without Tom22) in the presence of 1µM Bim-BH3 (PEIWIAQELRRIGDEFNAYYA), Bid-BH3 (ESQEDIIRNIARHLAQVGDSMDRSIPPG) (Genscript), or BTSA1 (Medchem). The concentration refers to the whole mix (reaction mix + feeding mix) because the sizes of the three molecules are below the cut-off of the dialysis membrane. Blot is representative of 2 independent experiments.

(C) Same experiments as in **(B)** in the presence of Tom22. Blot is representative of 2 independent experiments.

(D) Same experiments as in Fig.3B in the presence of Tom22. Blot is representative of 2 independent experiments.

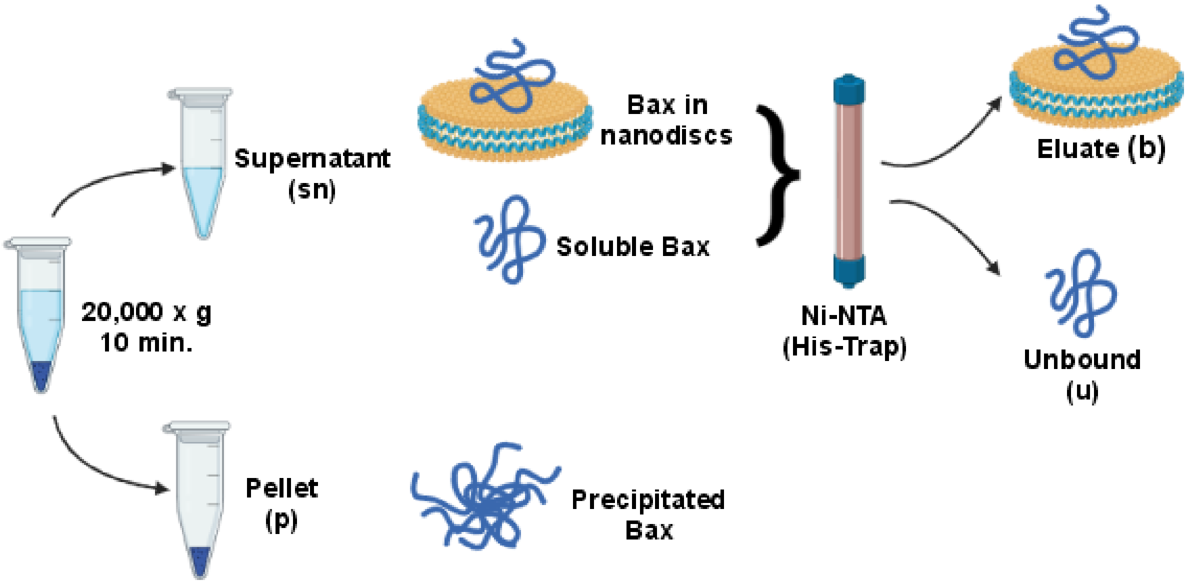

Figure S6. Schematic representation of the experimental flow.

After cell-free synthesis, a 15-minutes, 20,000 x g centrifugation led to a pellet (p) of precipitated proteins and a supernatant (sn) containing soluble Bax and nanodiscs (empty or with inserted Bax). The supernatant was loaded on Ni-NTA (His-Trap). The flow-through contained soluble Bax unbound to nanodiscs (u). After an elution with 300mM imidazole, the eluate contained nanodiscs (empty or with inserted Bax) (b).

Table S1: antibodies used in this study

Bax	sc-20067, 2D2, Santa-Cruz Biotechnology	Mouse monoclonal	1:10000 PBST/milk<br>IP (1µg)
Bax	sc-23559, 6A7, Santa-Cruz Biotechnology	Mouse monoclonal	IP (1µg)
Bax	ab182734, Abcam	Rabbit monoclonal	1:10000 PBST/milk
Tom22	ab179826, Abcam	Rabbit monoclonal	1:5000 PBST/milk<br>inhibition (1µg)
Tom20	ab186735, Abcam	Rabbit monoclonal	1:5000 PBST/milk
His6 tag	ma1-135, ThermoFisher Scientific	Mouse monoclonal	1:10000 PBST/milk
HA tag	71-5500, ThermoFisher Scientific	Rabbit polyclonal	1:10000 PBST/milk
StrepII tag	2-1507-001, IBA Life Sciences	Mouse monoclonal	1:5000 PBST/milk
FITC	701078, ThermoFisher Scientific	Rabbit monoclonal	Quenching (4µg)
Bcl-xL	E18, ab32370, Abcam	Rabbit monoclonal	1:10000 TBST/milk
Cyt.c	NB100-56503, R&D Systems	Mouse monoclonal	1:10000 PBST/milk
VDAC	MSA03, Mitosciences	Mouse monoclonal	1:10000 PBST/milk
