# Supplementary material for: The membrane insertion of the pro-apoptotic protein Bax is a Tom22-dependent multi-step process: a study in nanodiscs
Source: Cell Death Discov. 2024 Jul 23;10:335. doi: 10.1038/s41420-024-02108-x (PMC11266675; doi:10.1038/s41420-024-02108-x)

Fig1A_20230214

Bax

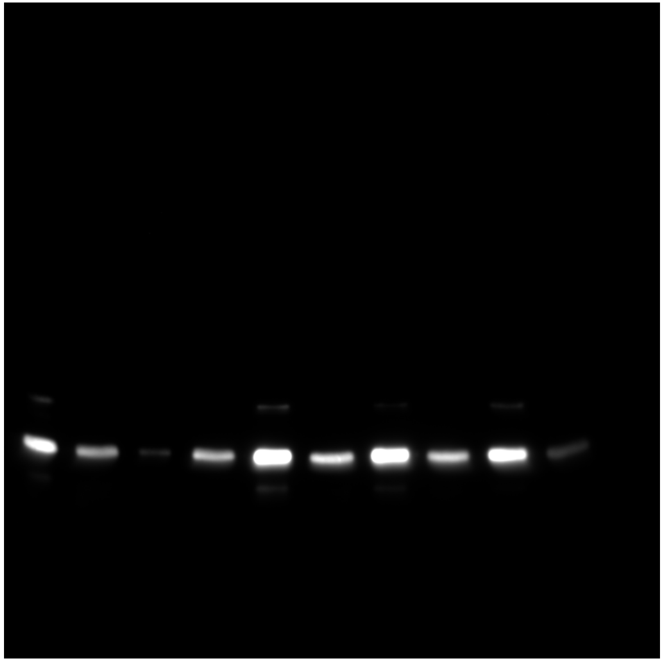

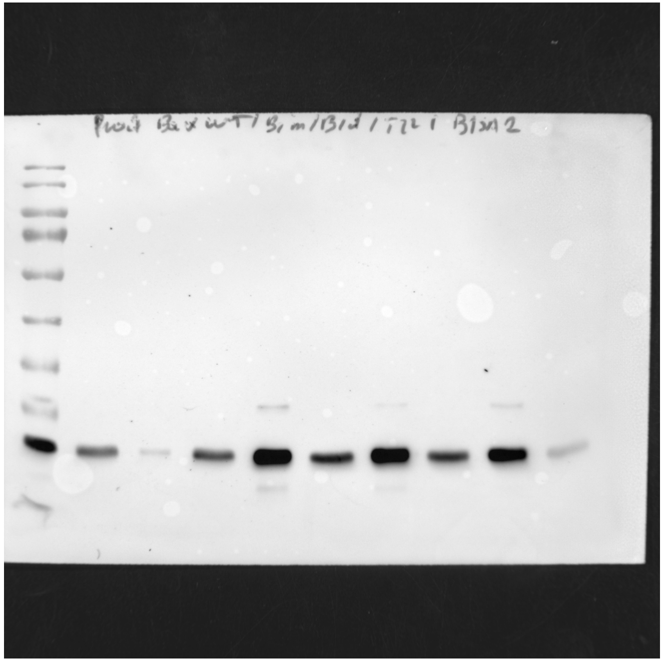

Fig1A_20230214

Tom22

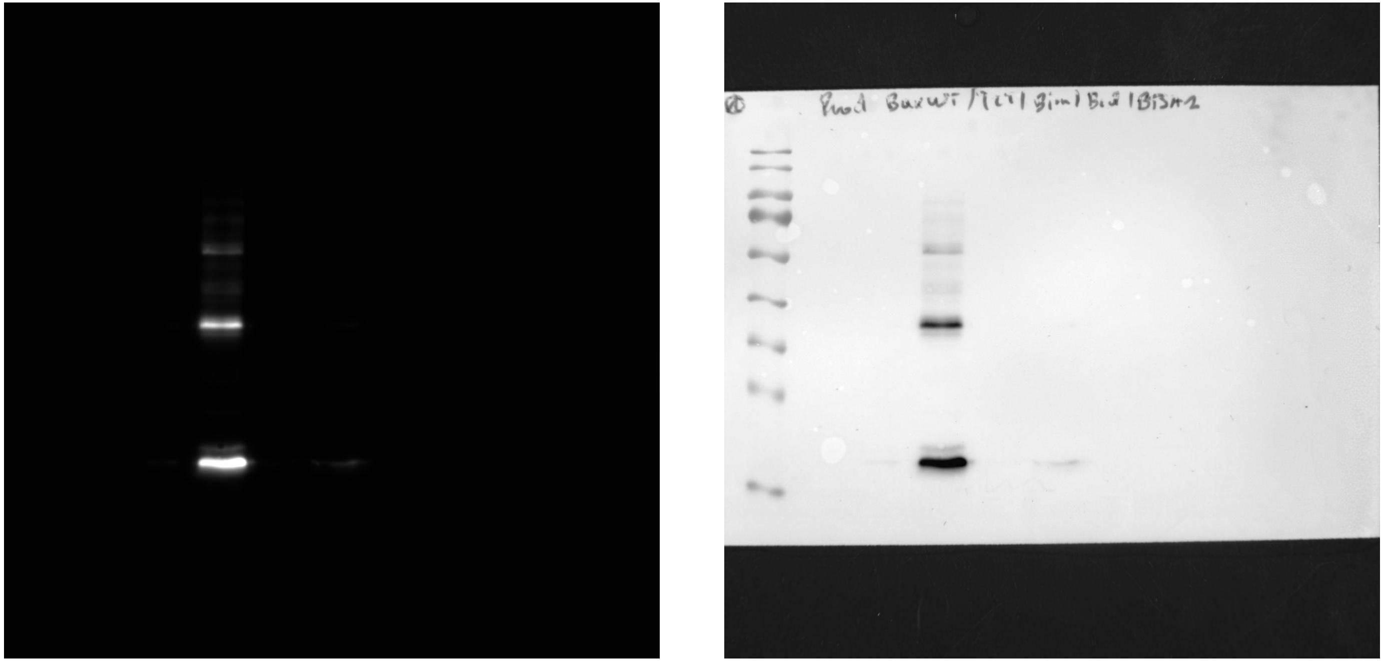

Fig1C_20230214

Bax

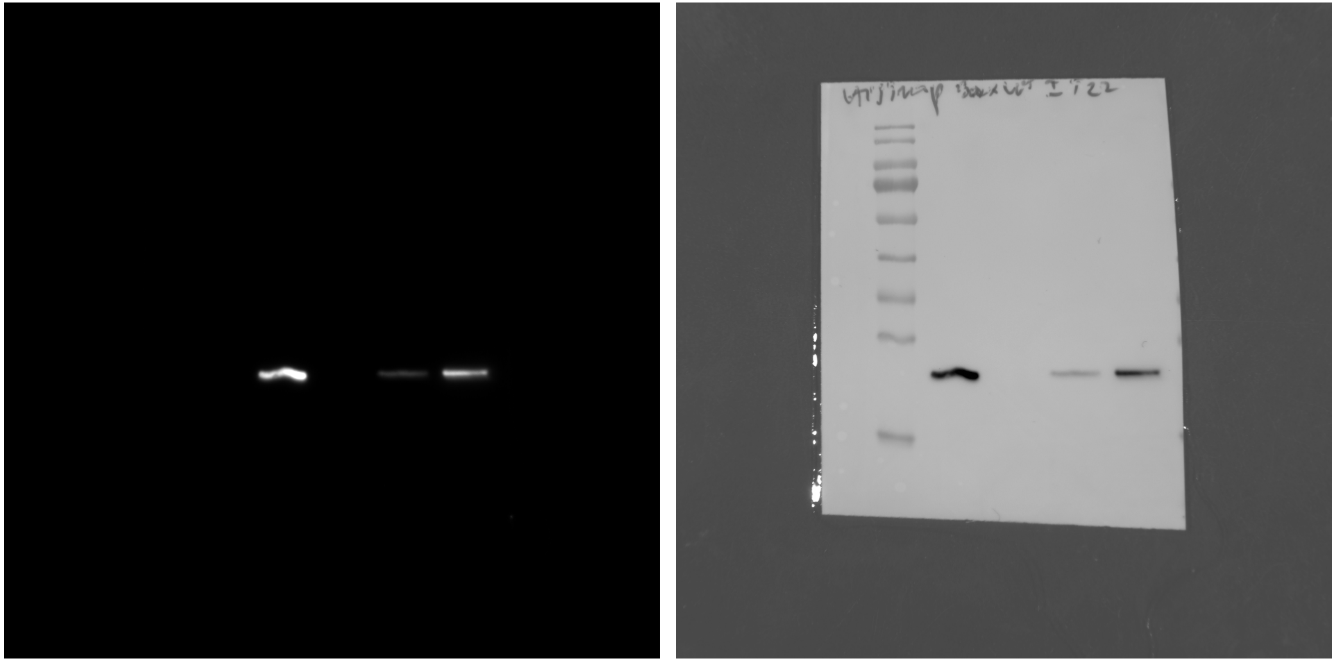

Fig1C_20230214

His6

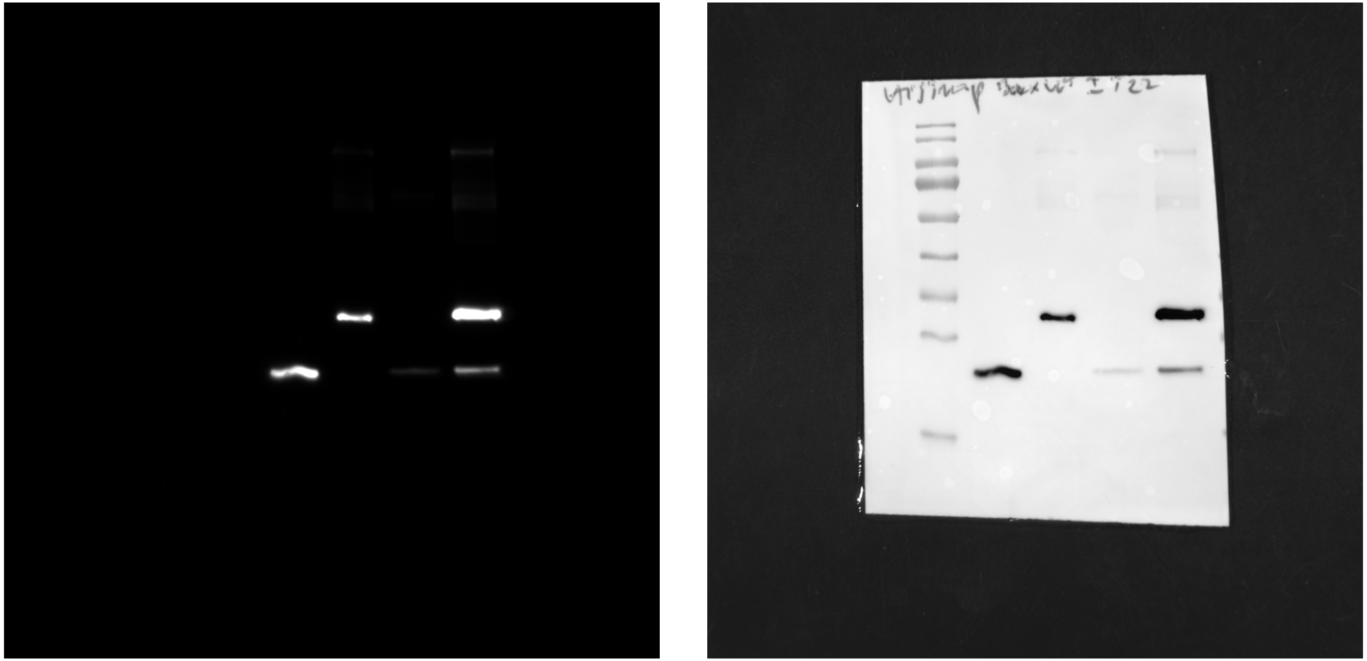

Fig1C_20230214

Tom22

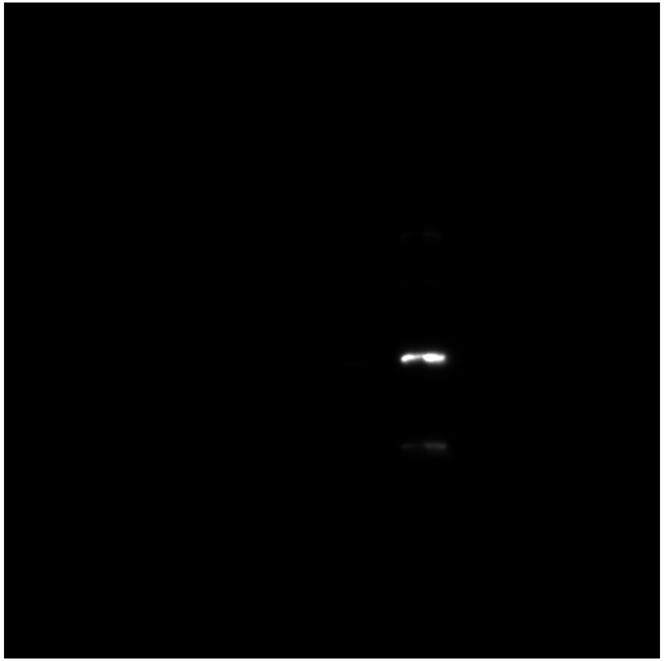

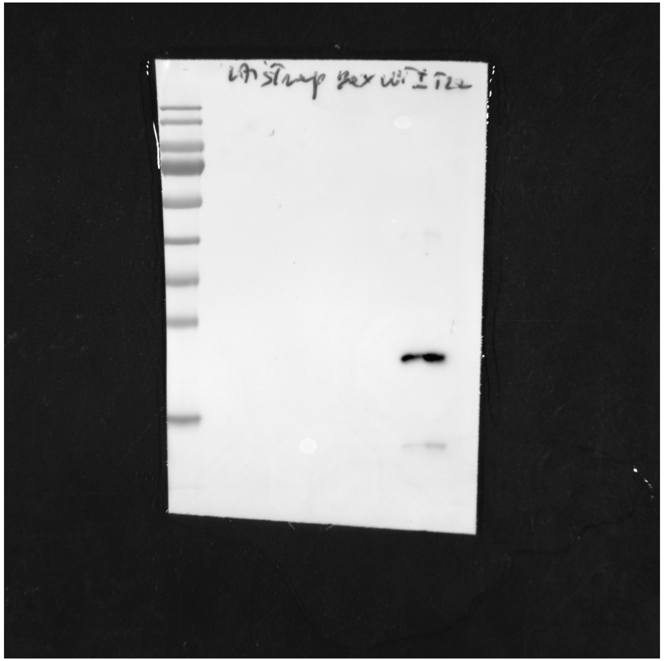

Fig1E_20221216

Bax

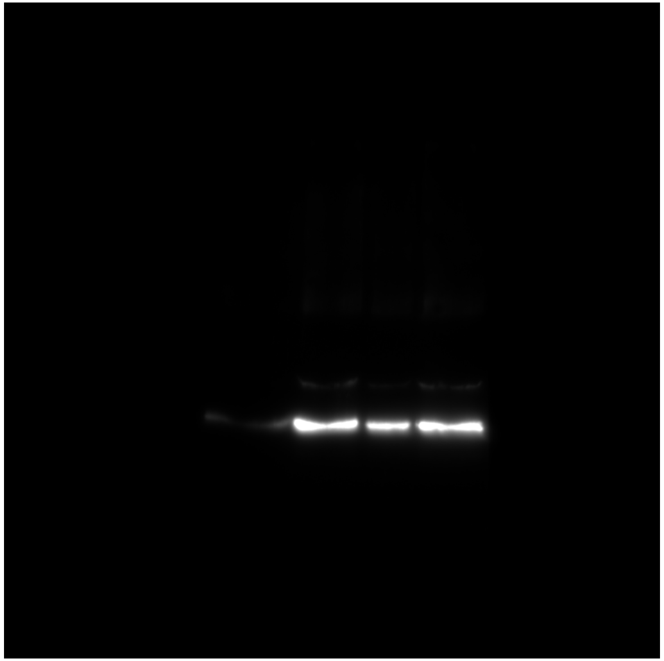

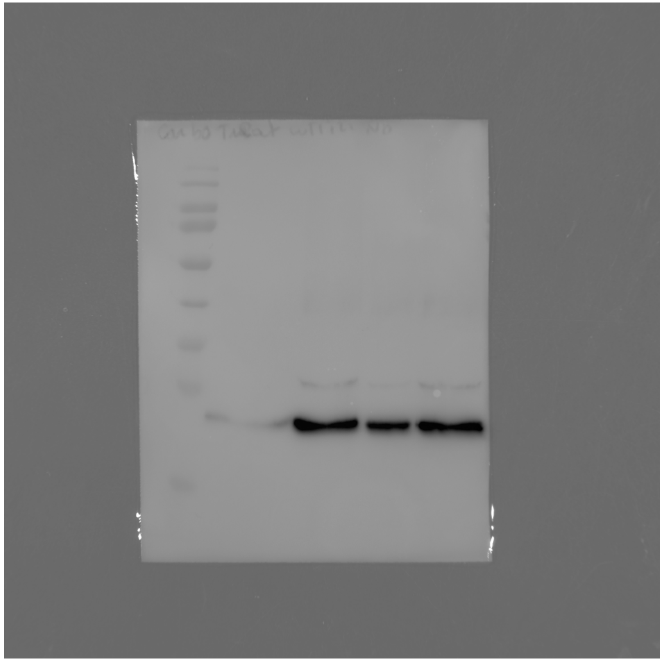

Fig1E_20221216

His6

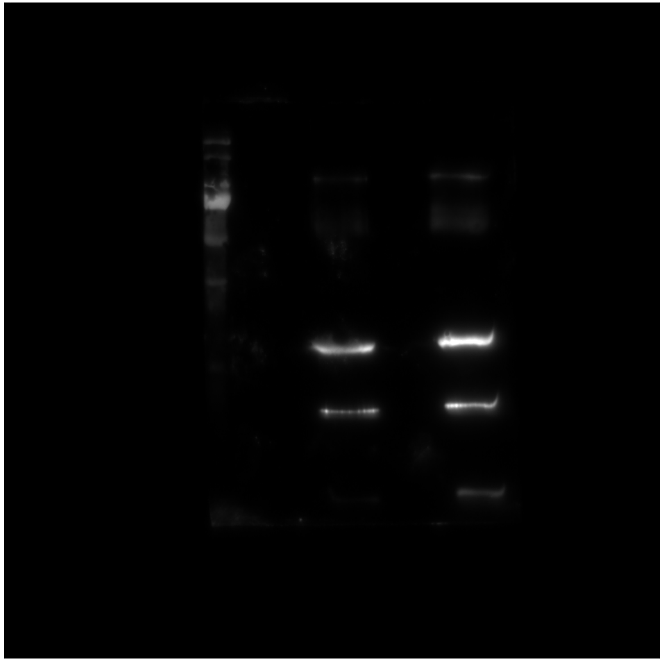

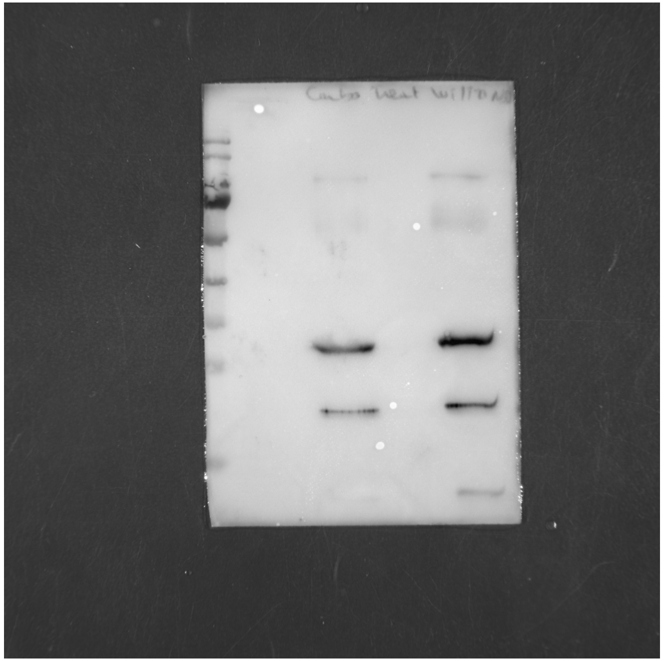

Fig1E_20221216

Tom22

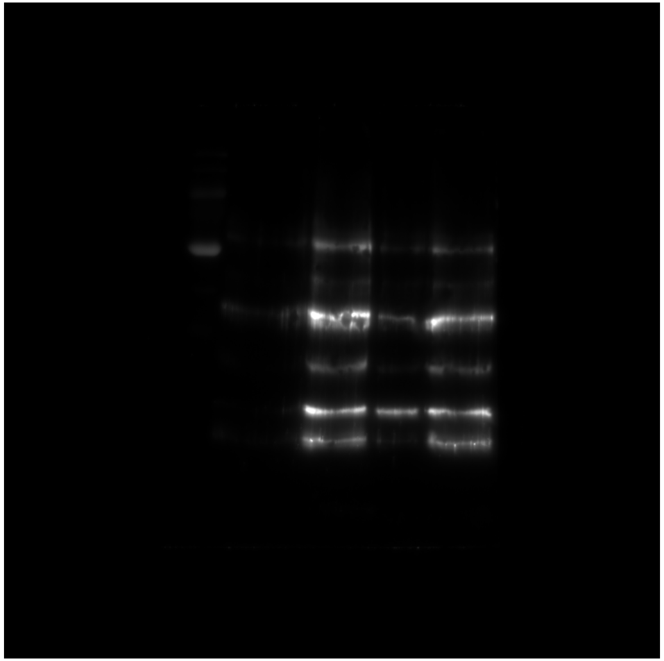

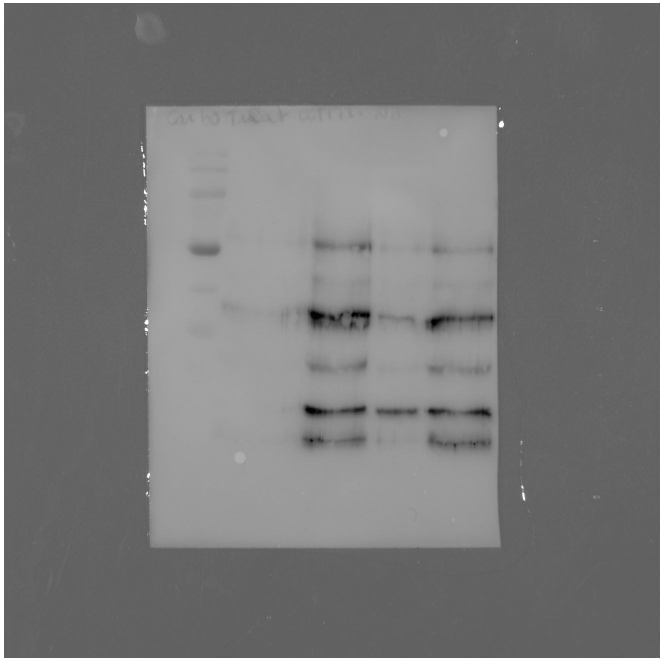

Fig1F_20230220

Bax

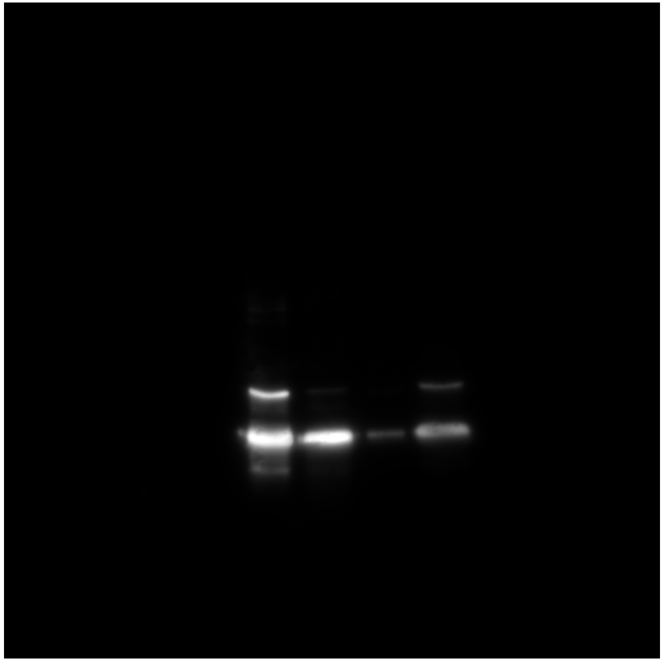

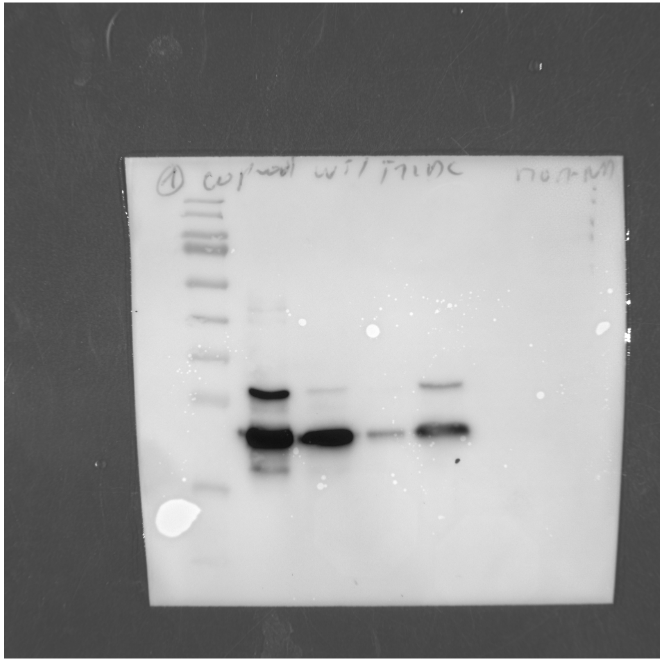

Fig1F_20230220

Tom22

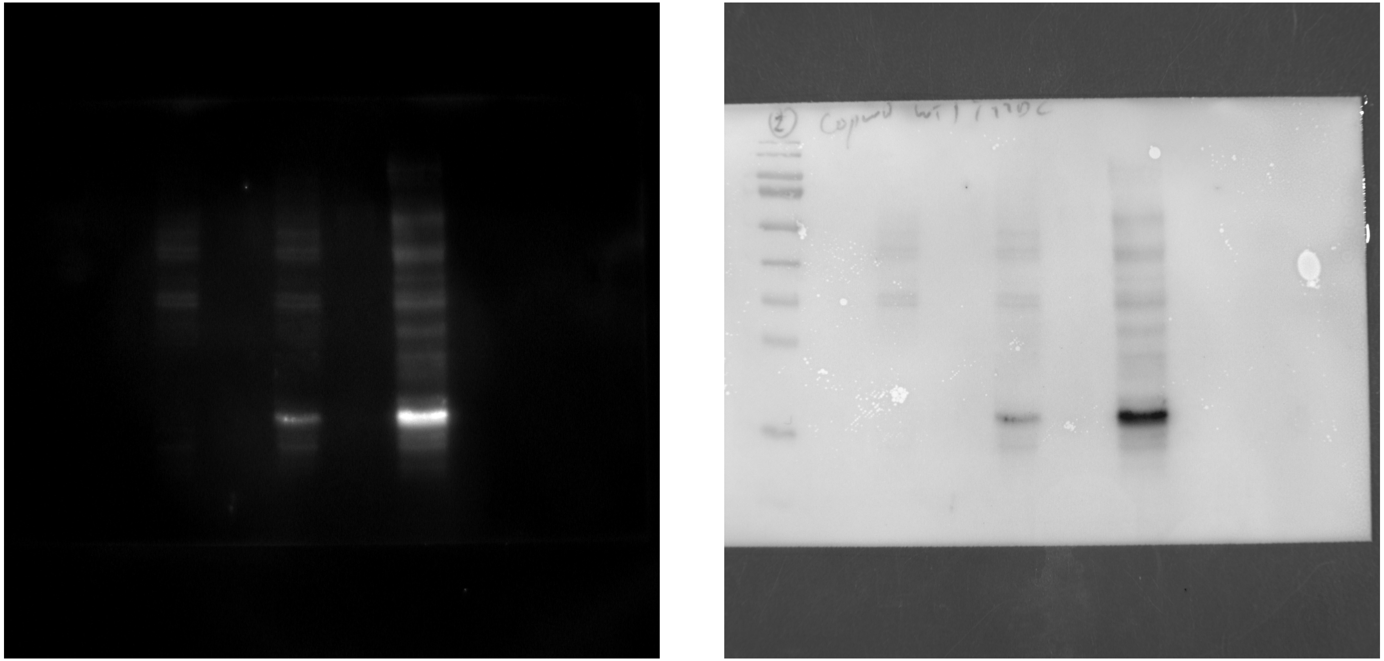

Fig1G_20230220

Bax

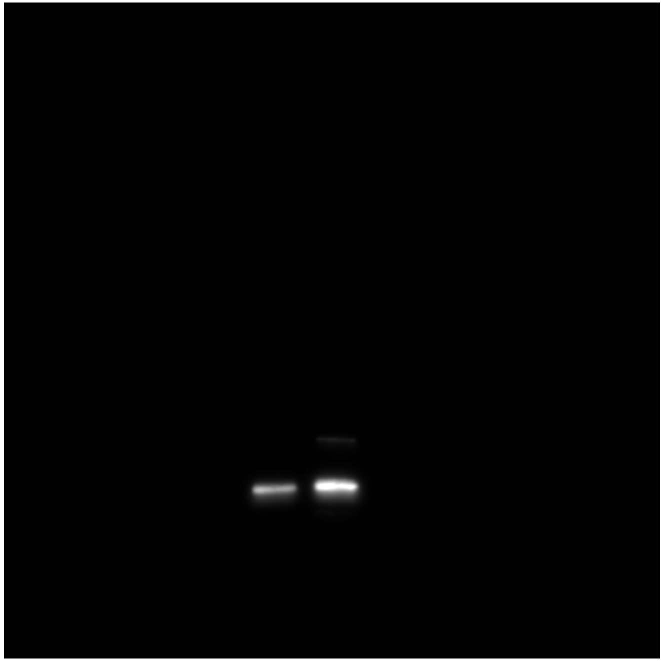

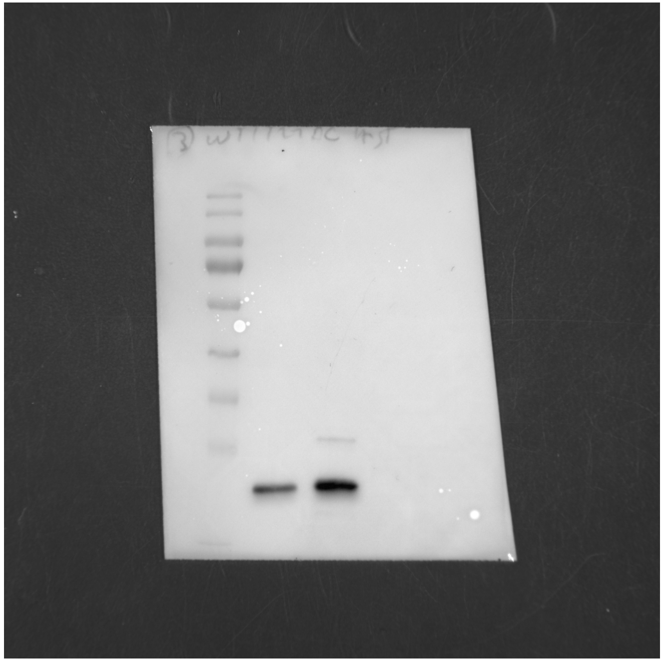

Fig1G_20230220

His6

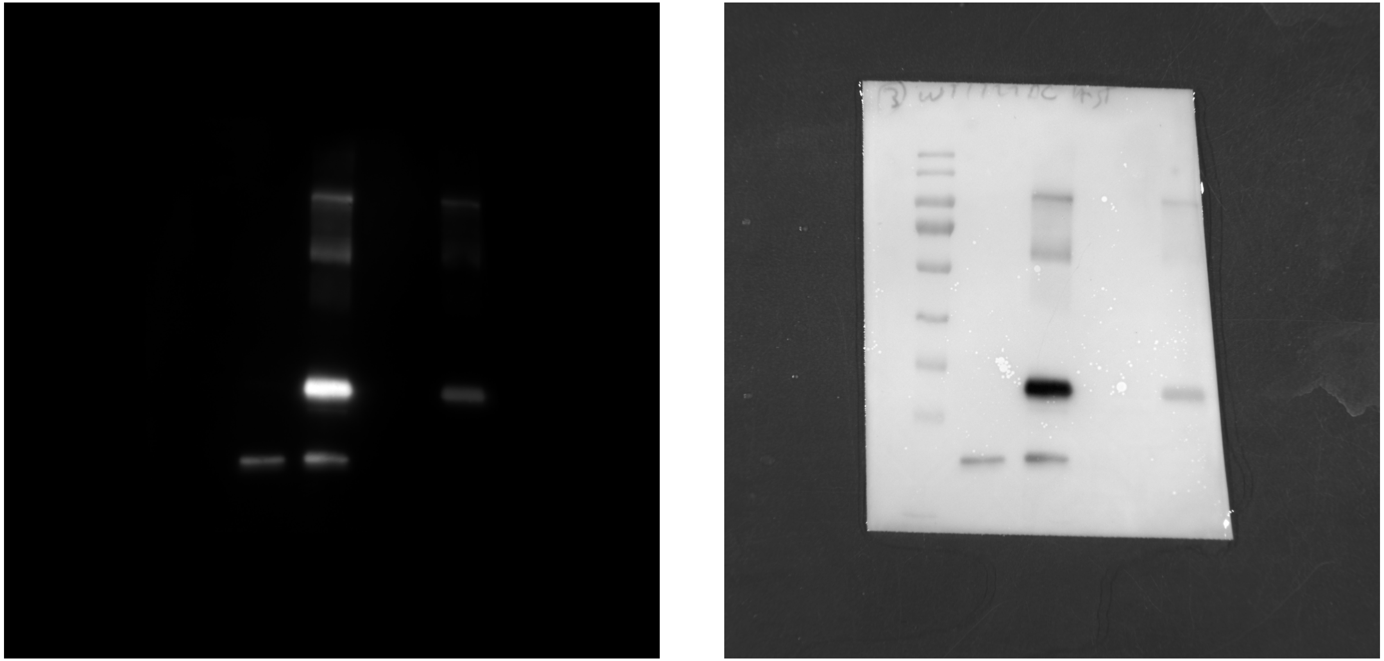

Fig1H_20230705

Bax

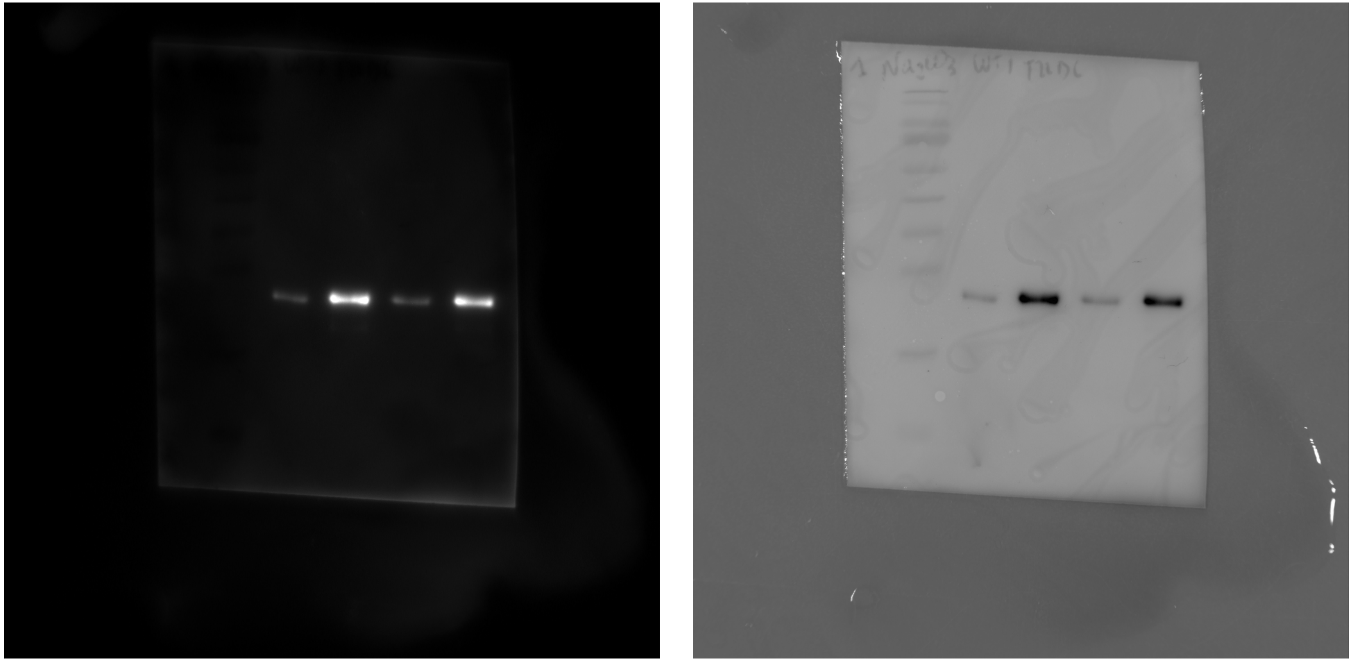

Fig1H_20230705

His6

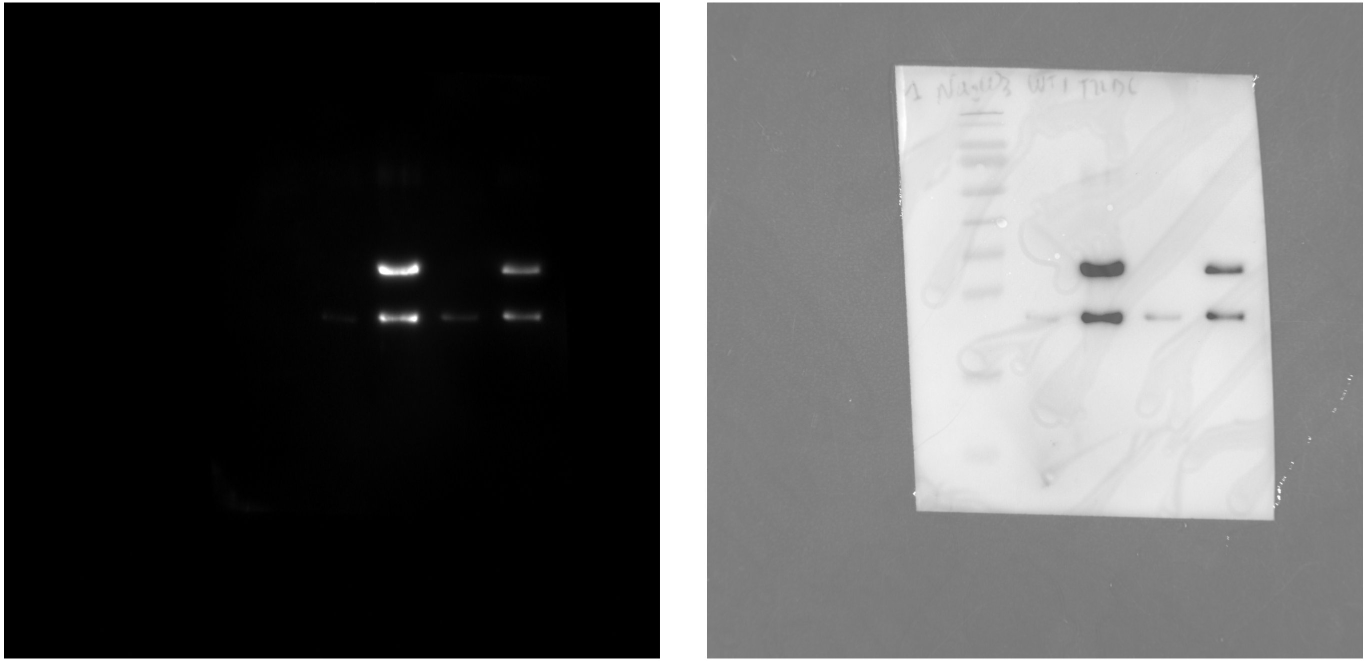

Fig2A_20230421_20230821

2d2

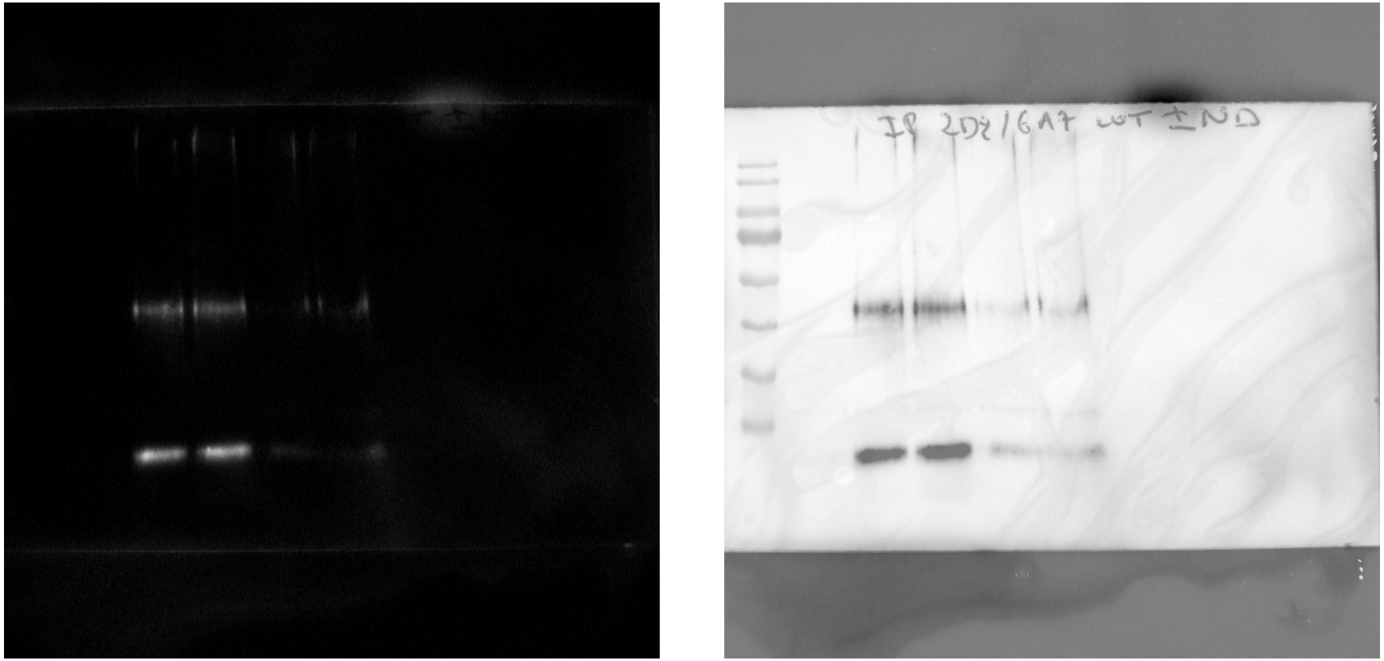

Fig2A_20230421_20230821

6a7

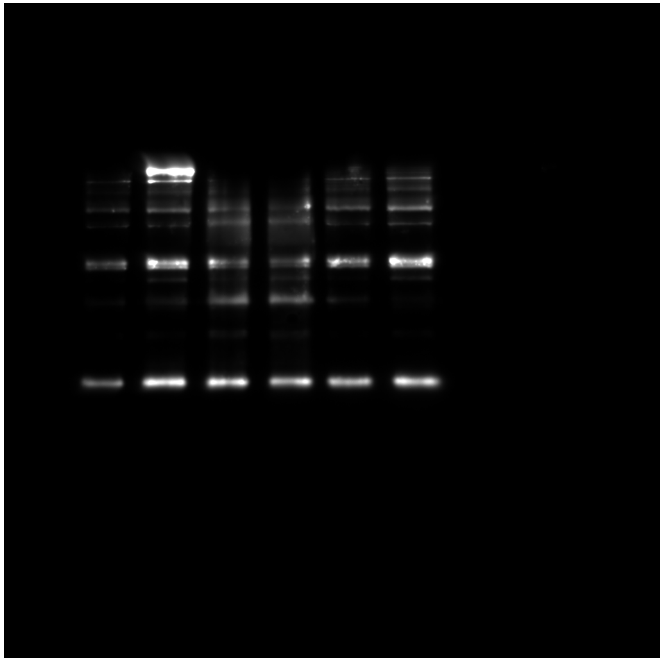

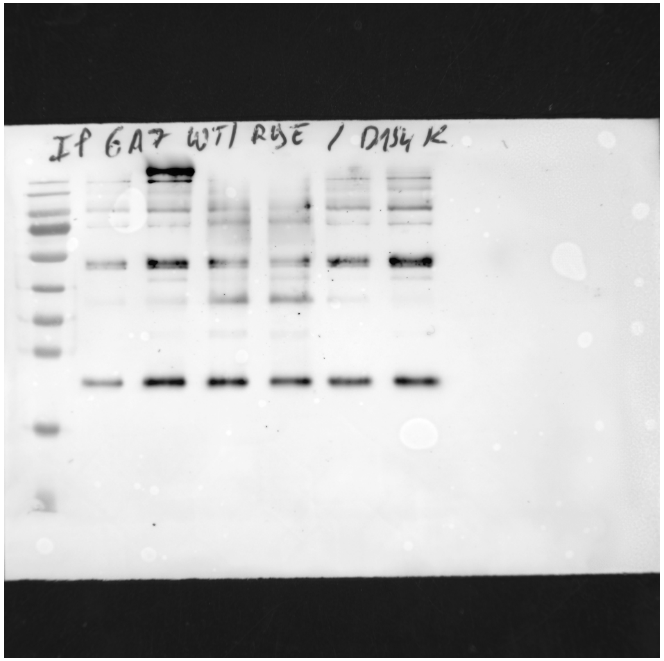

Fig2B_20221103

Bax

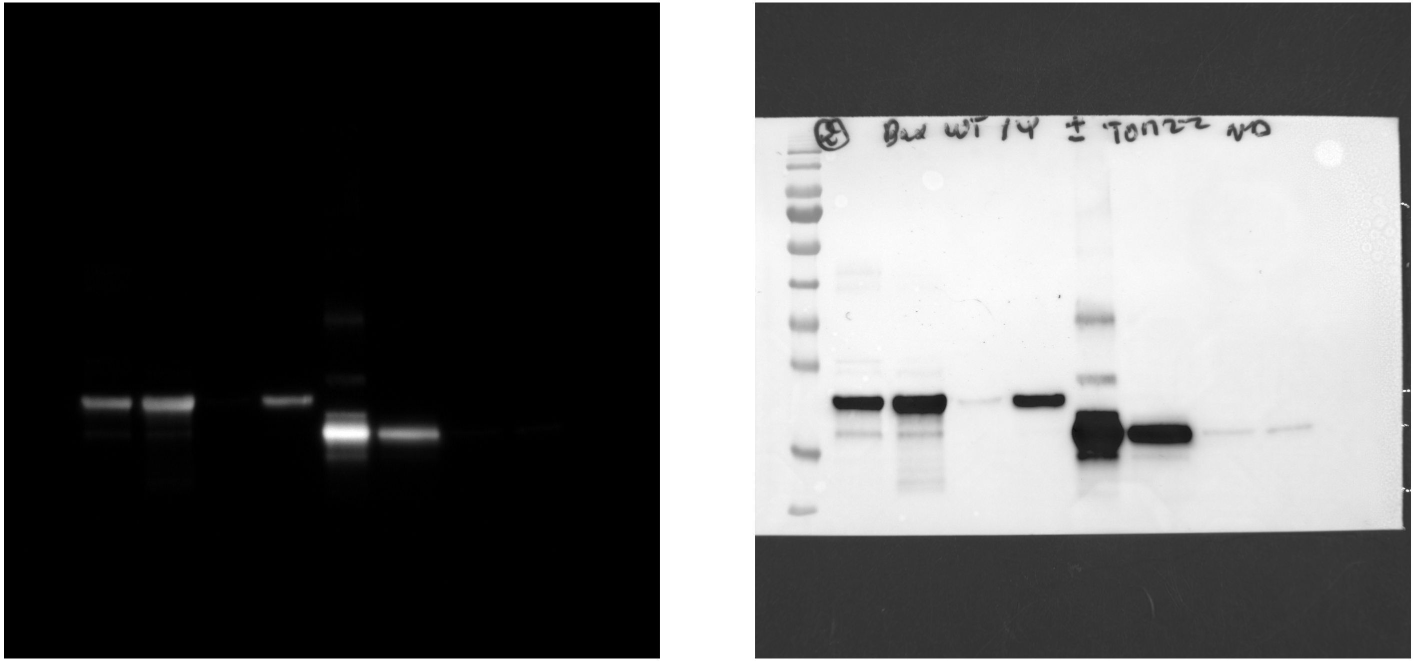

Fig2C_20230228

Bax

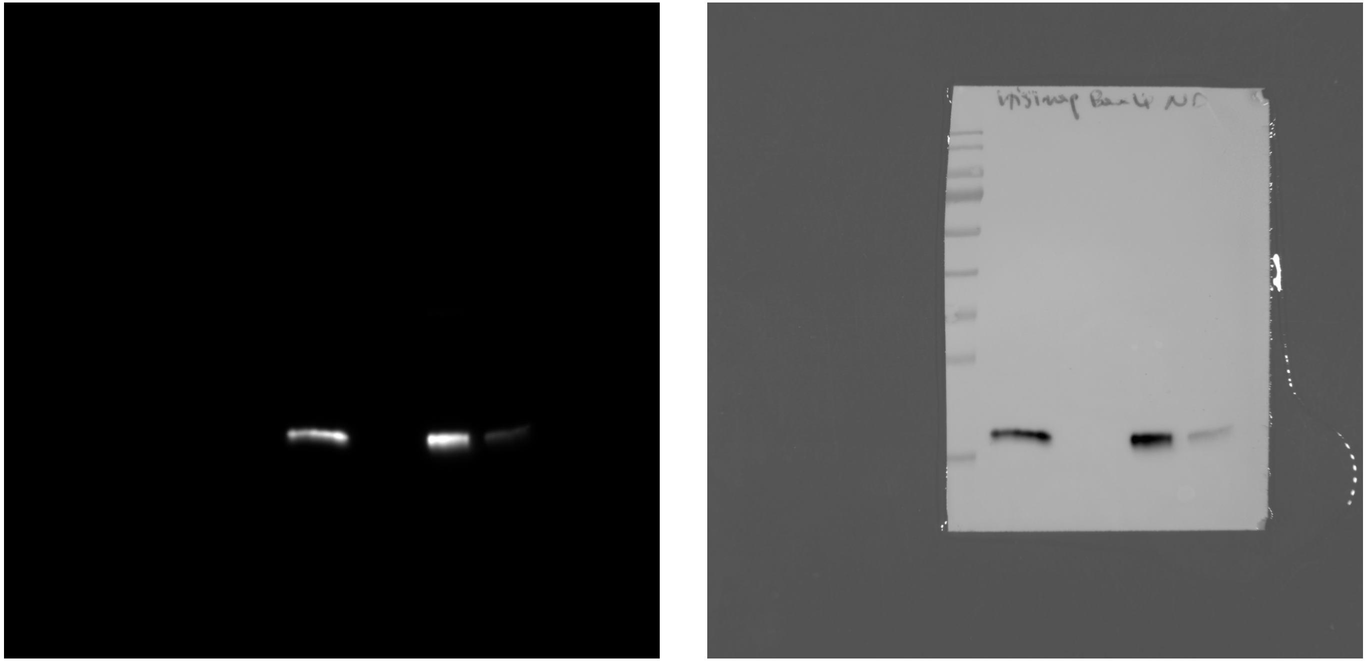

Fig2C_20230228

His6

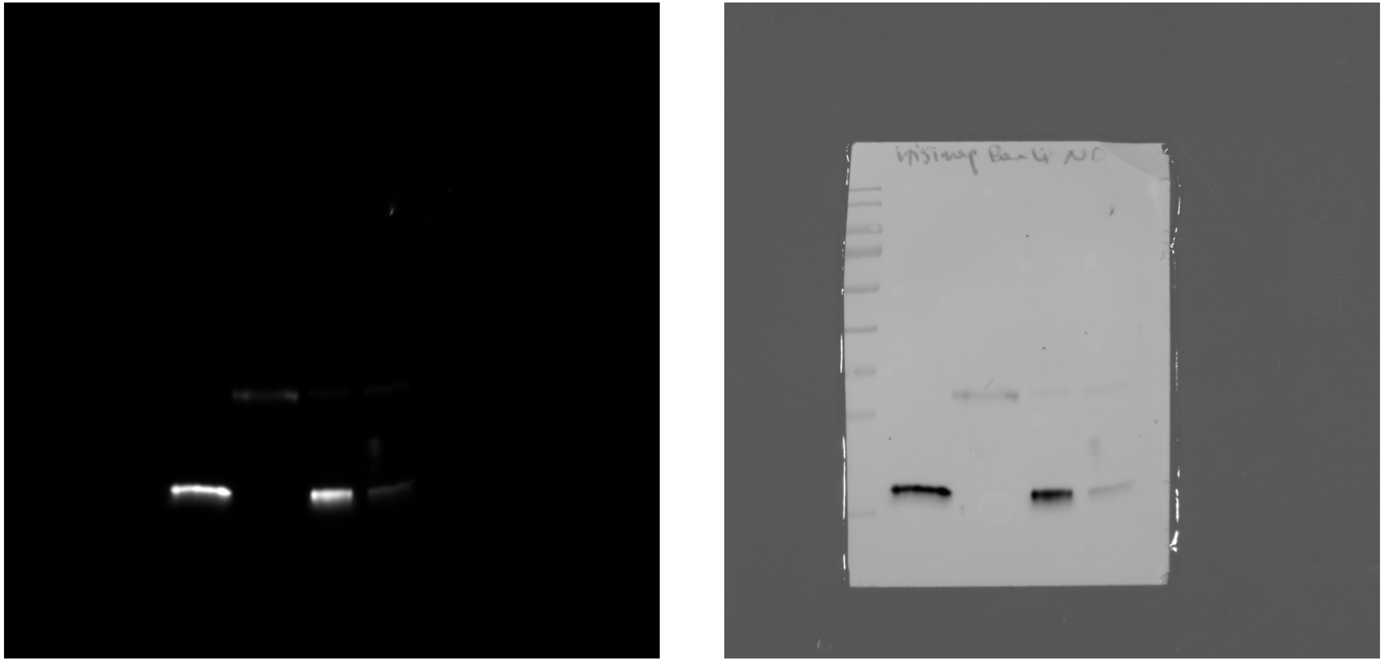

Fig2D_20221201

Bax+Tom22

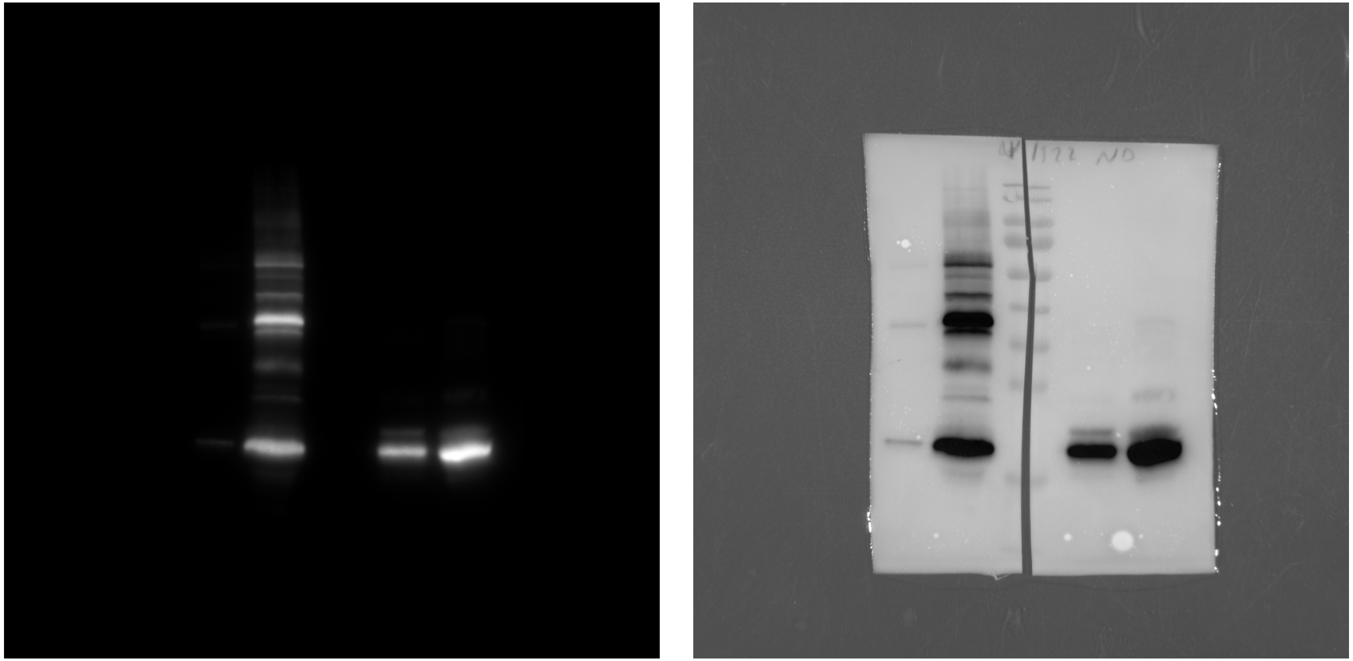

Fig2E_20221201

Bax

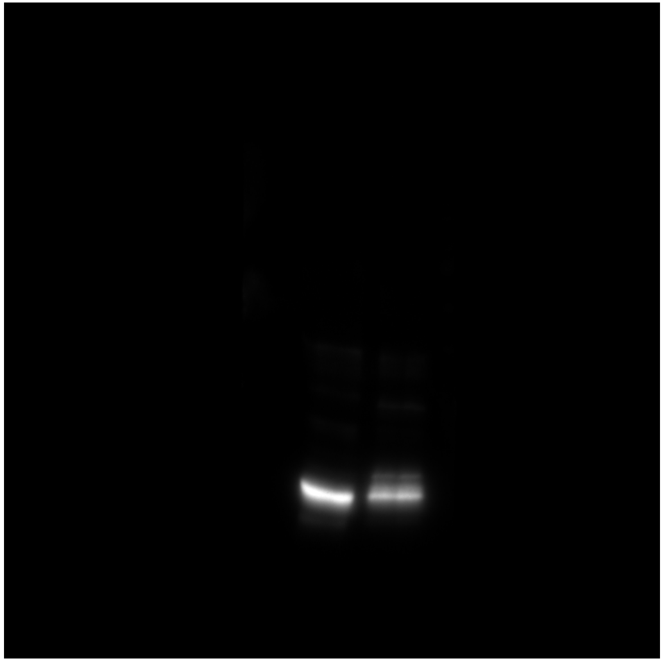

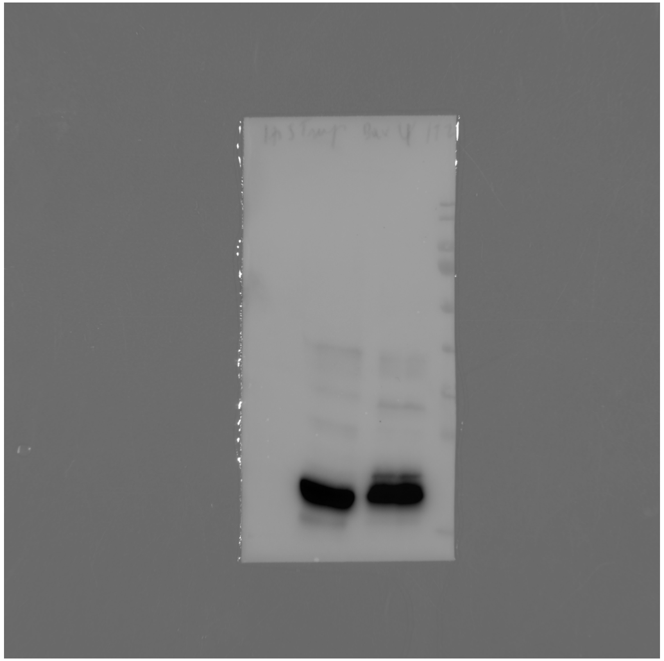

Fig2E_20221201

Tom22

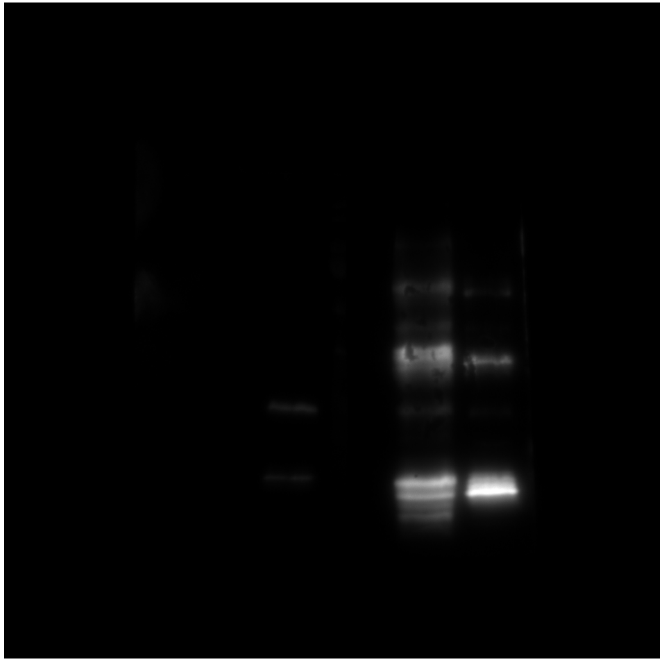

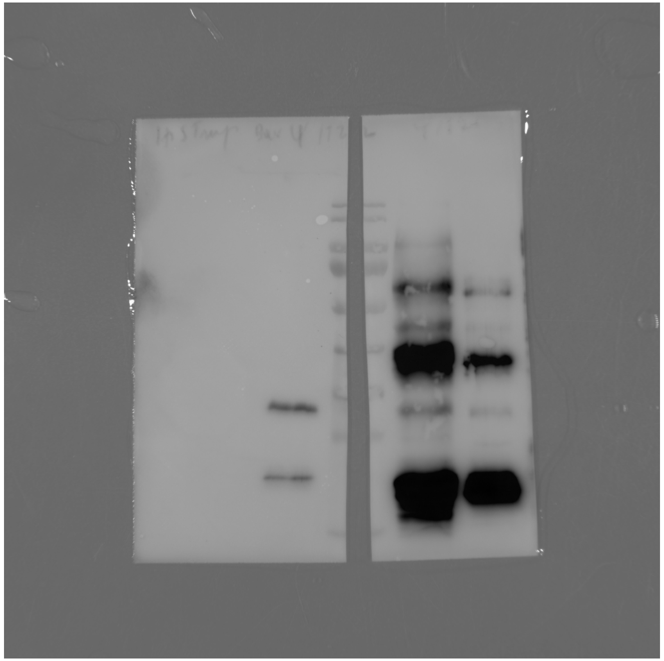

Fig2E_20221201

His6 (+Tom22)

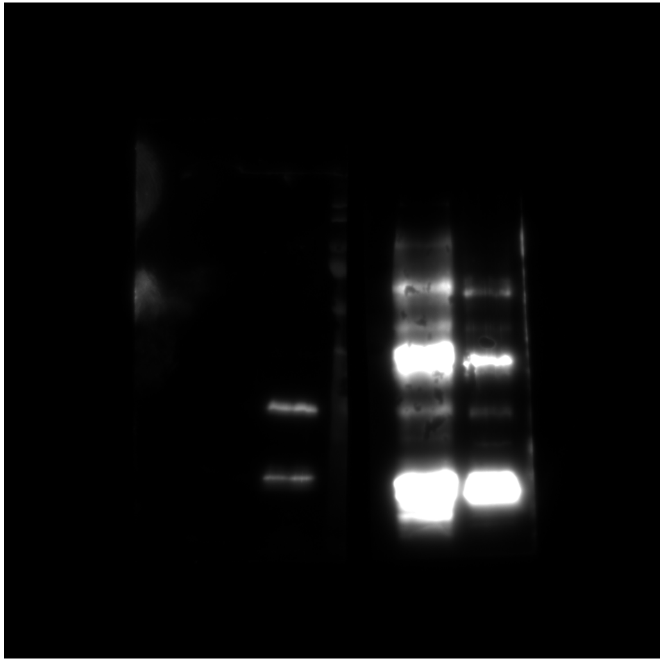

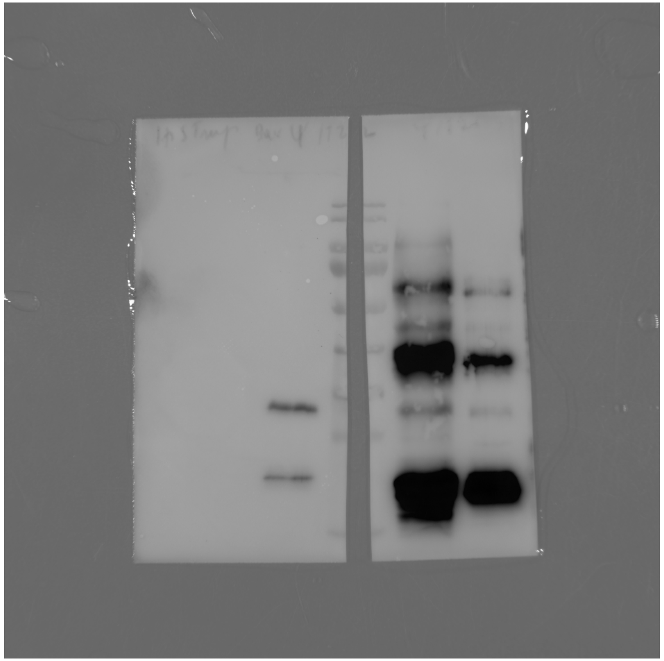

Fig2G_20230830_20230816

BaxL26Q

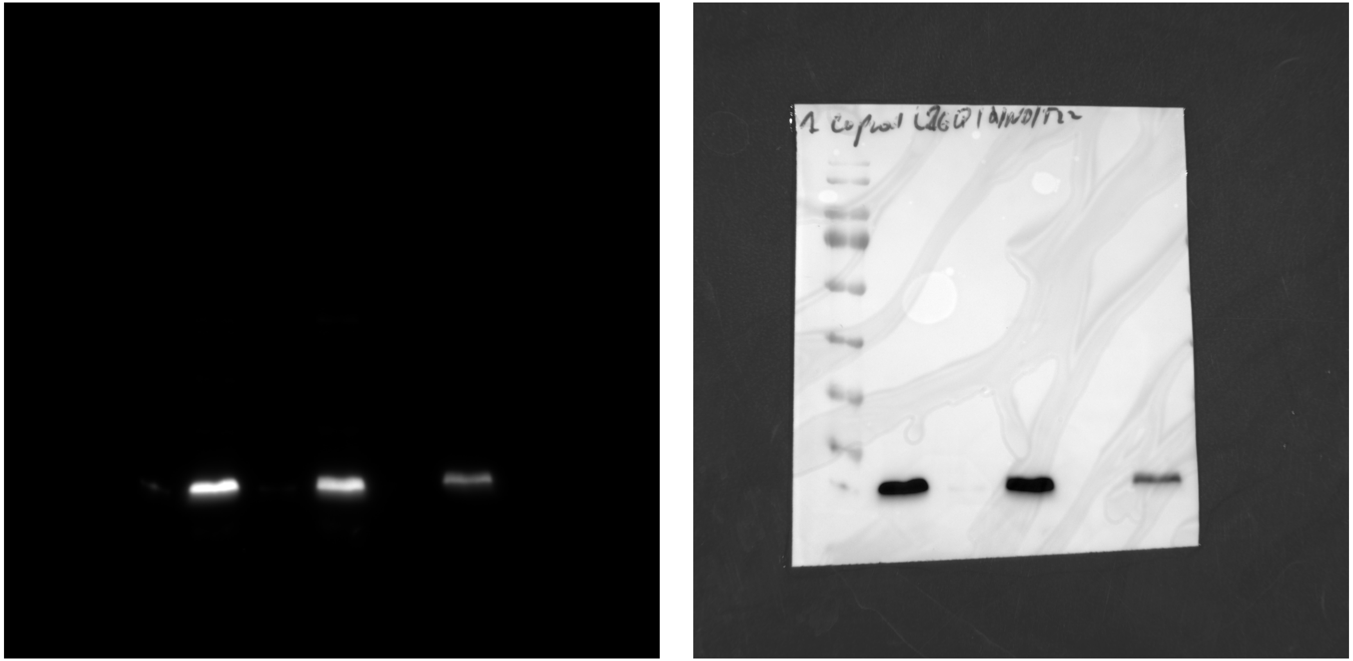

Fig2G_20230830_20230816

BaxWT + BaxA24R

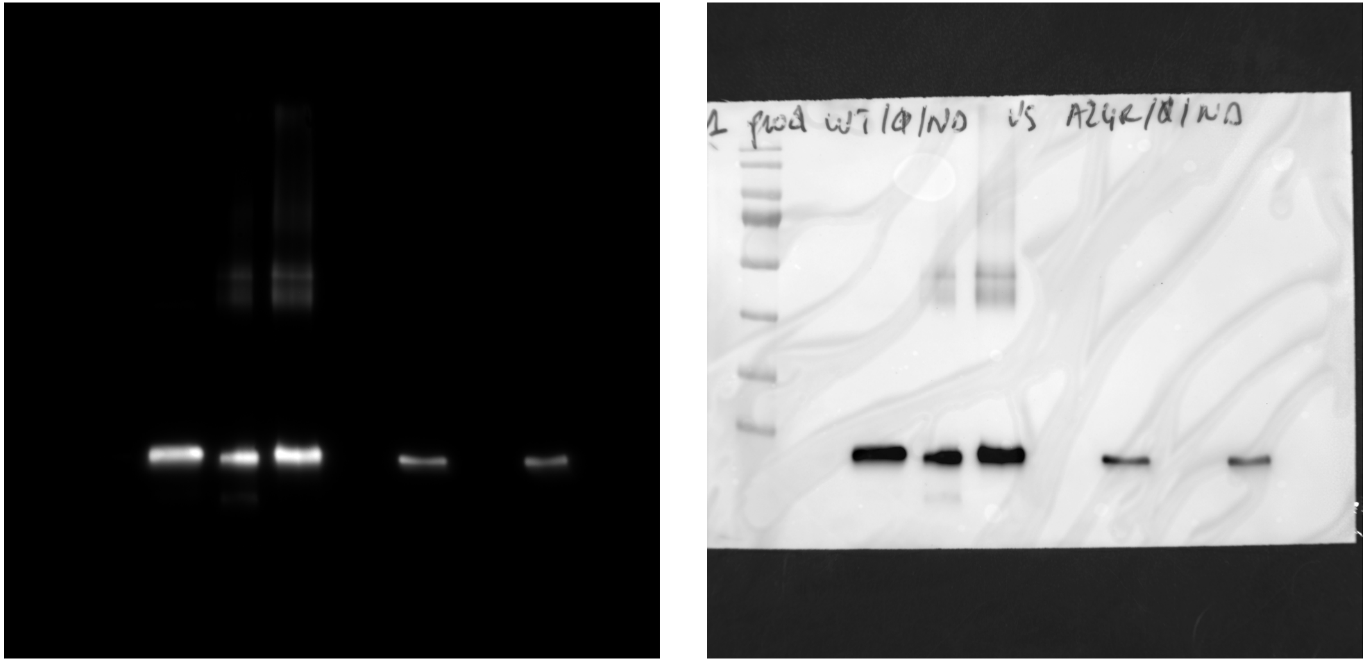

Fig2H_20230726

Bax

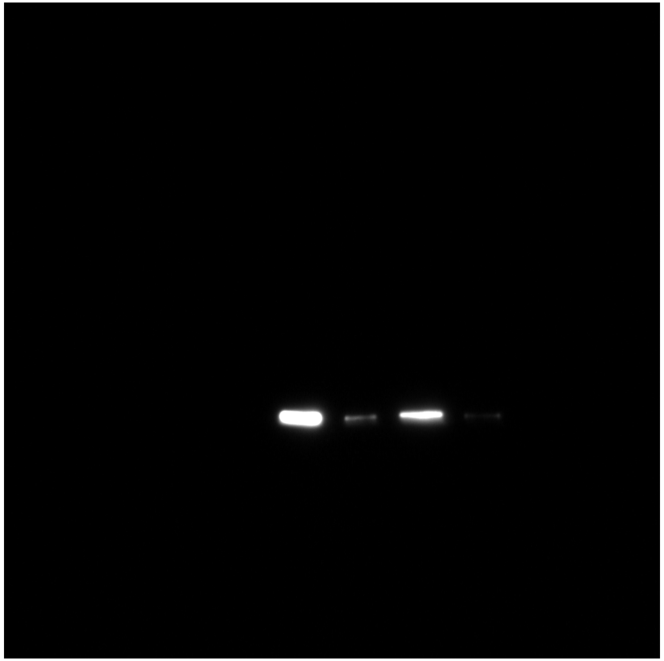

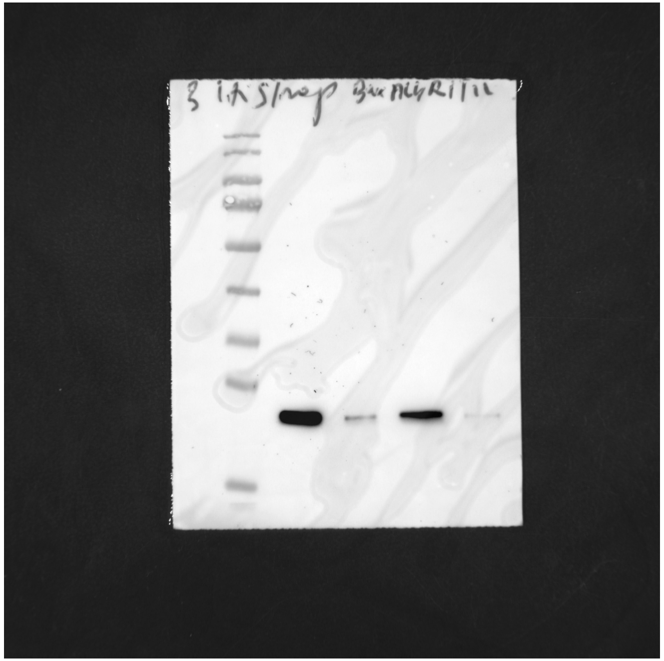

Fig2H_20230726

His6

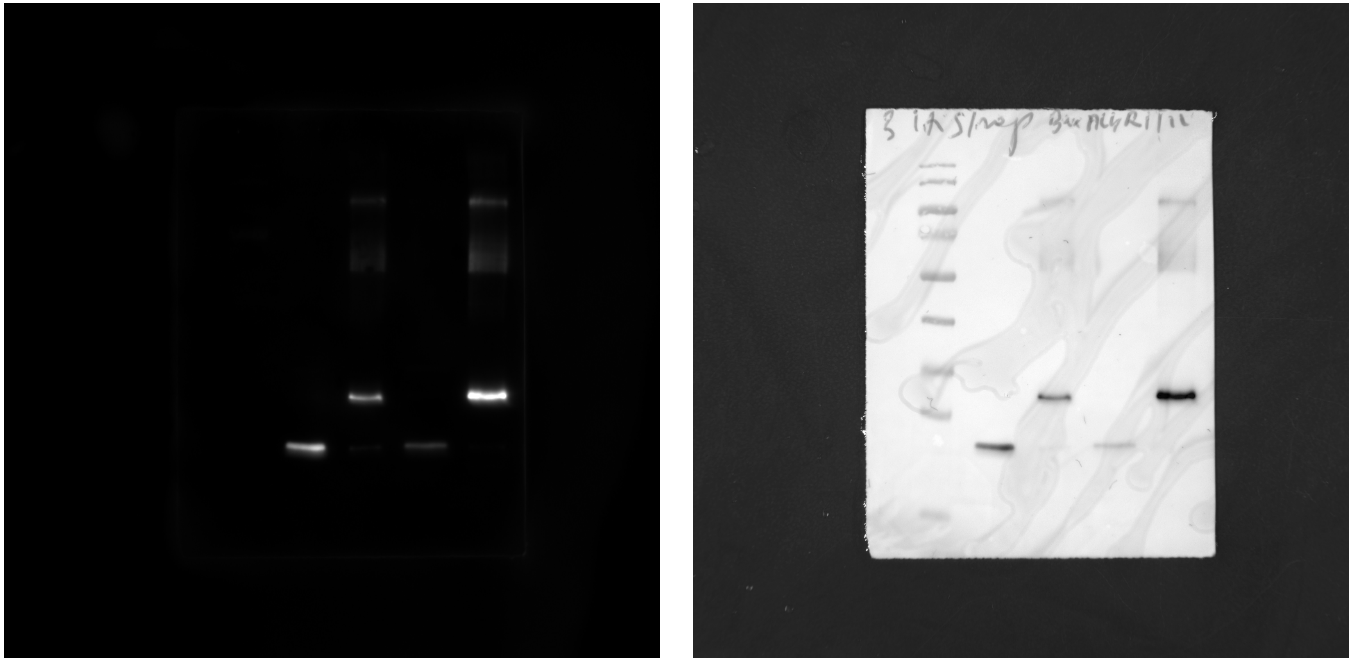

Fig2H_20230726

Tom22

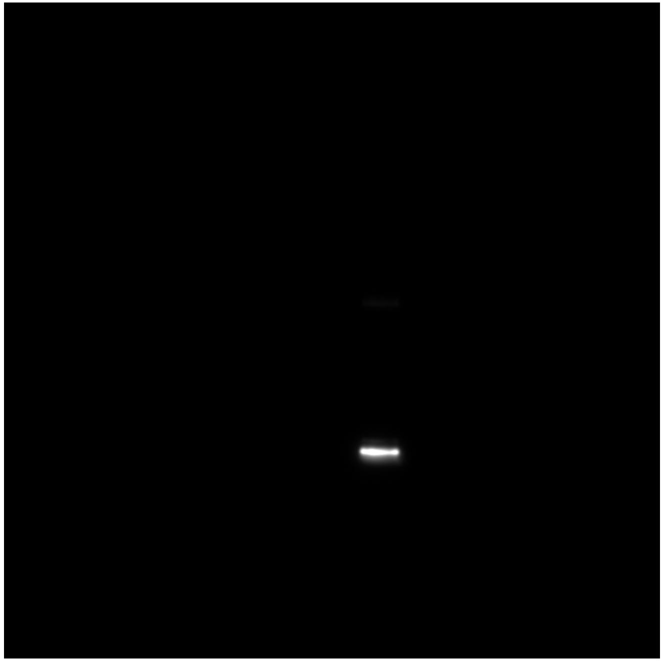

Fig3A_20230809

Bax+His6

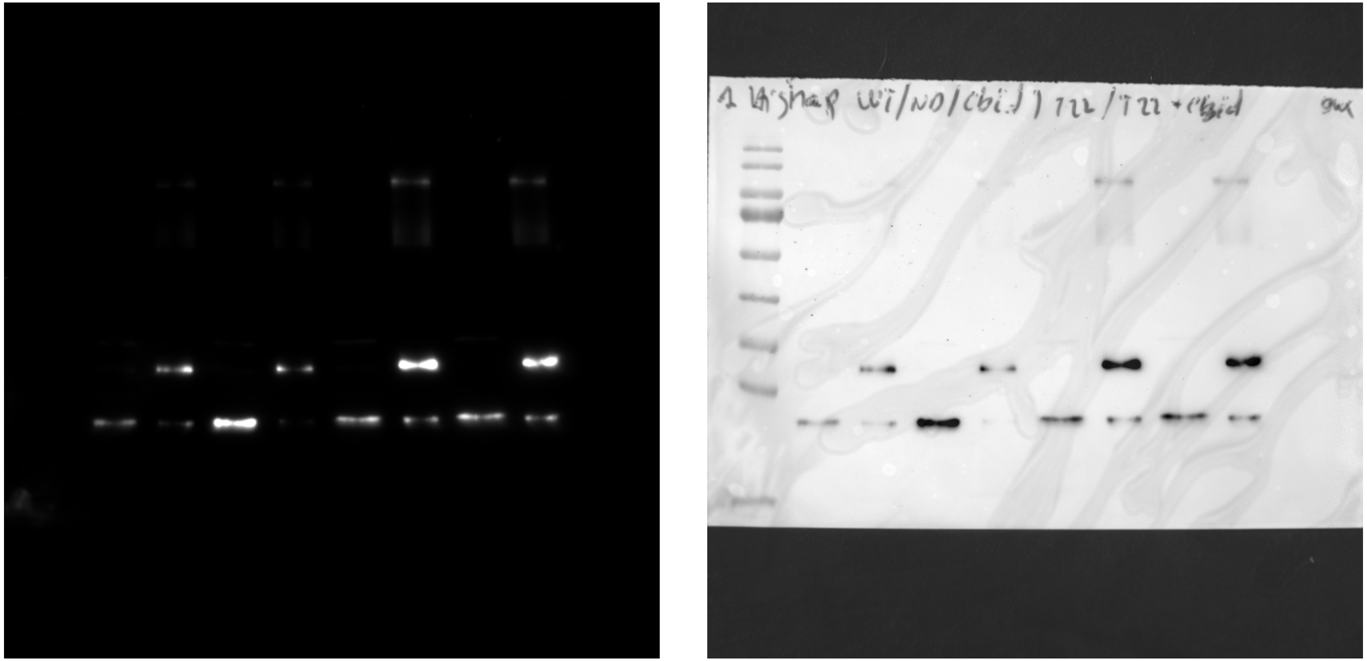

Fig3B_20230216

Bax

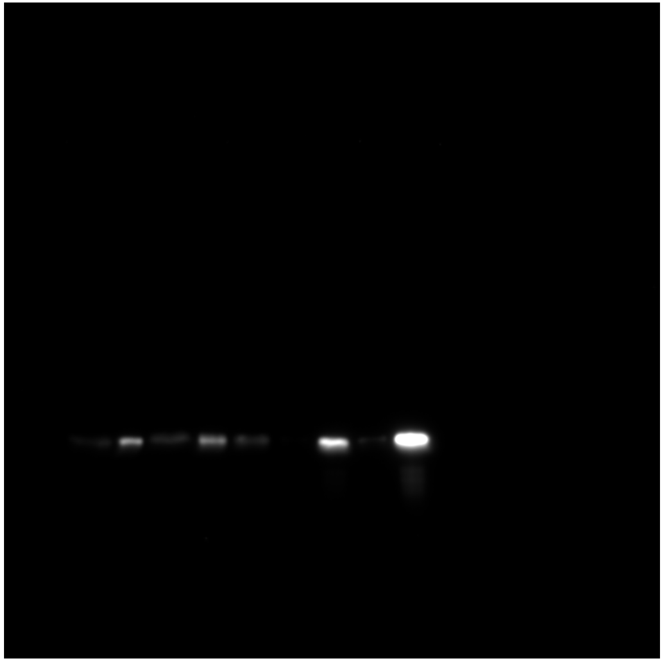

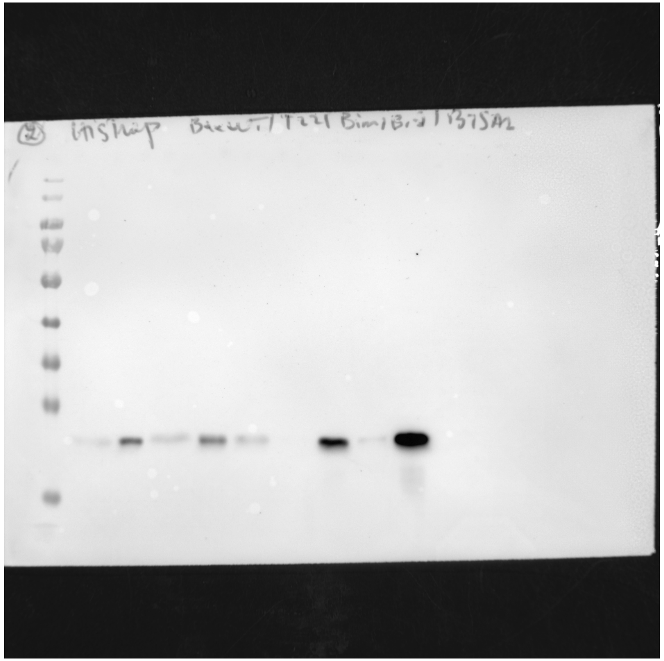

Fig3B_20230216

His6

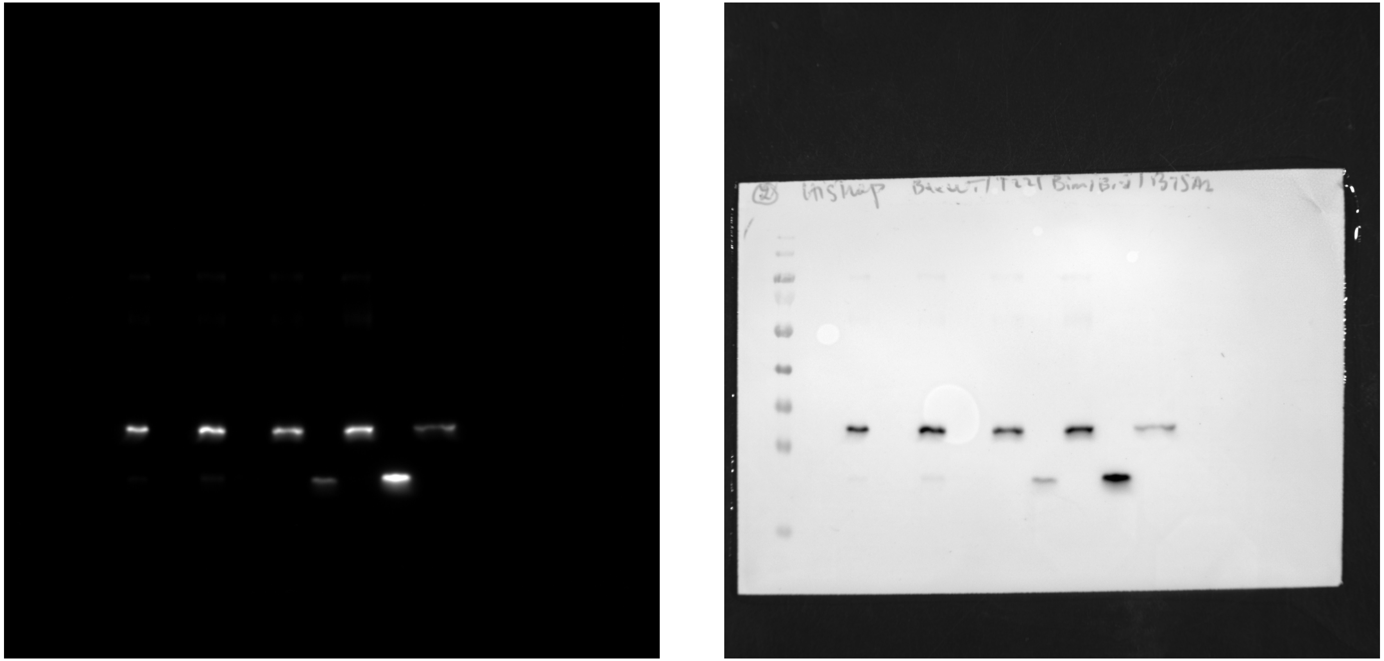

Fig3C_20230602

Bax

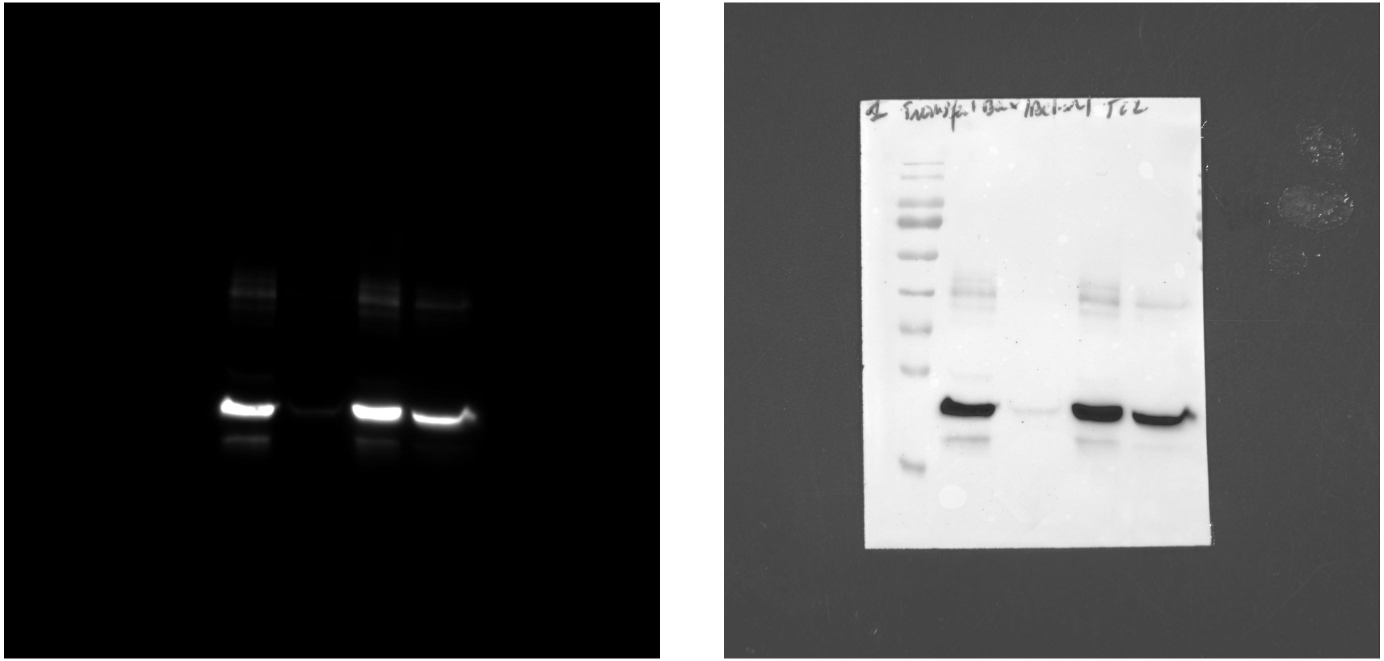

Fig3C_20230602

Bcl-xL

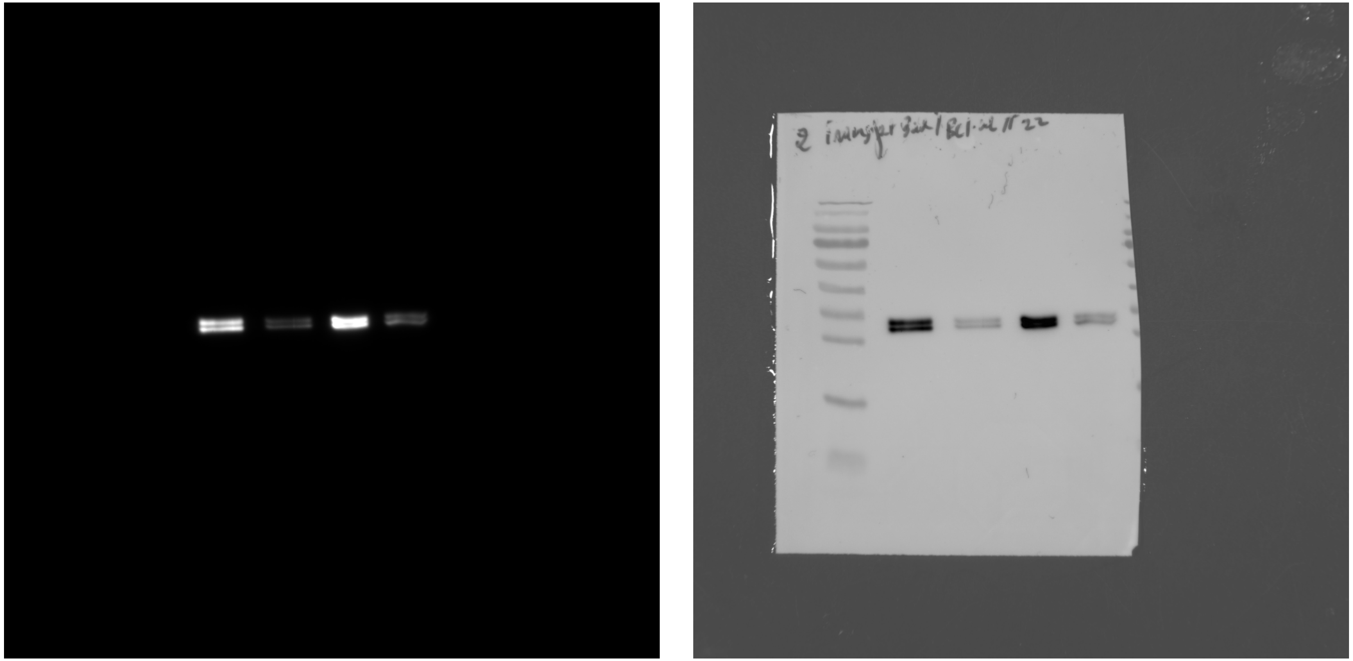

Fig3C_20230602

StrepII

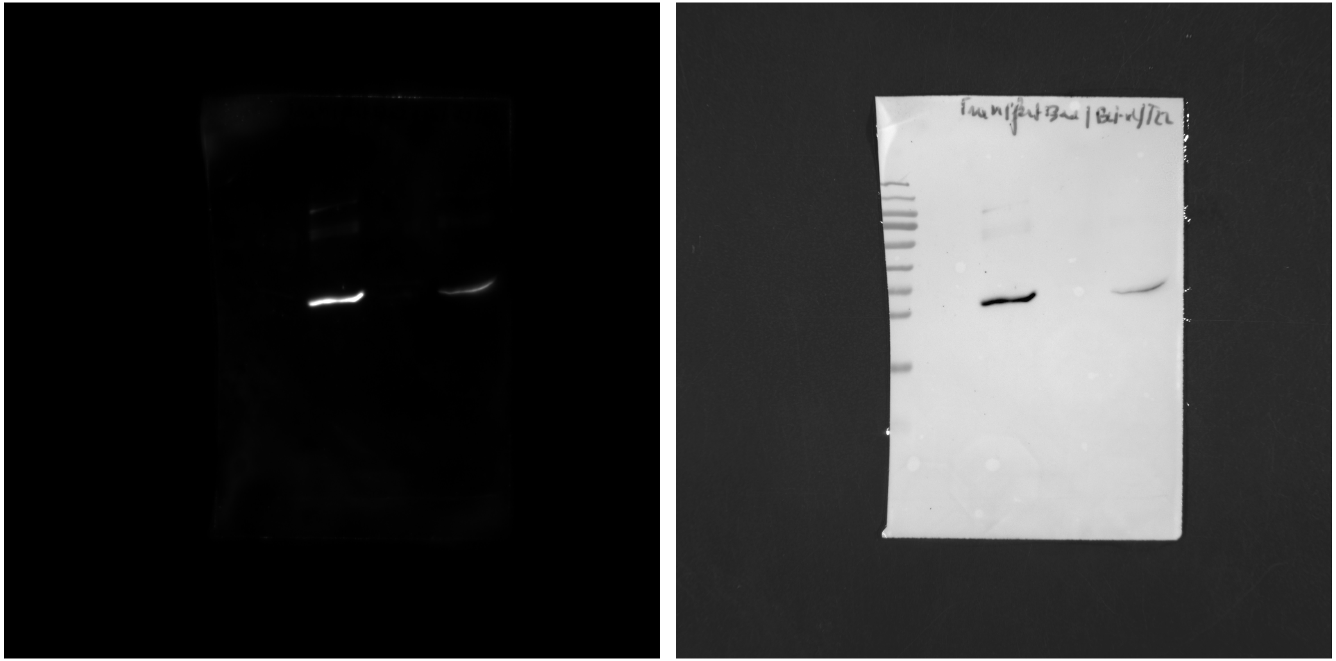

Fig3C_20230602

Tom22

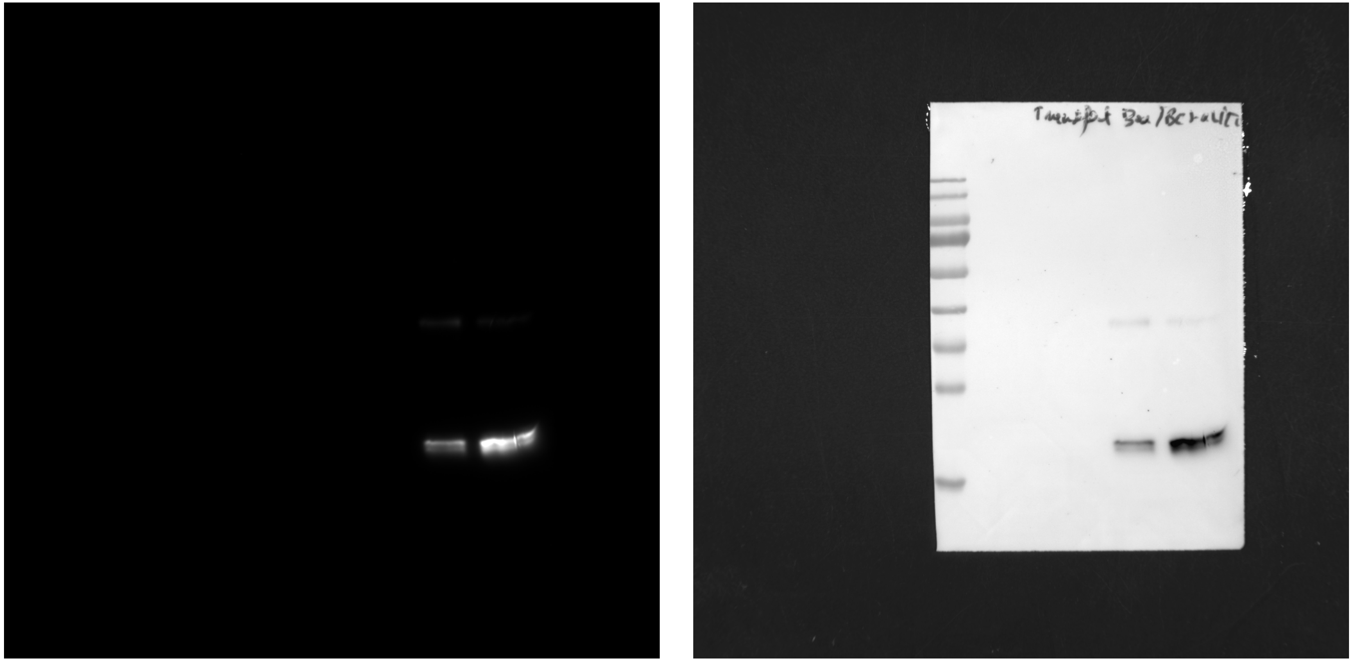

Fig3E_20230621

cytc

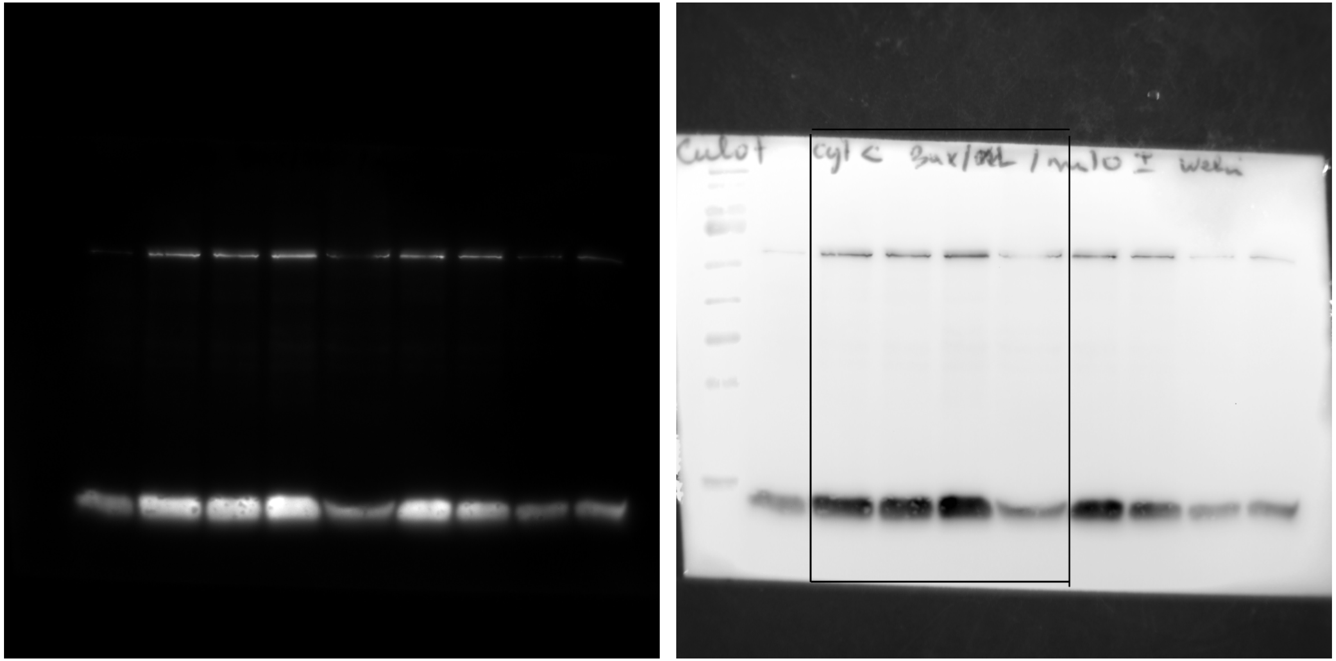

(the selection rectangle shows the lane used for the Figure. The 4 outside lanes on the right were done with a constitutively active Bax mutant, and the outside lane on the left was done with purified Bax (wihout nanodiscs). These were omitted for the clarity of the experiment.

Fig3E_20230621

porin

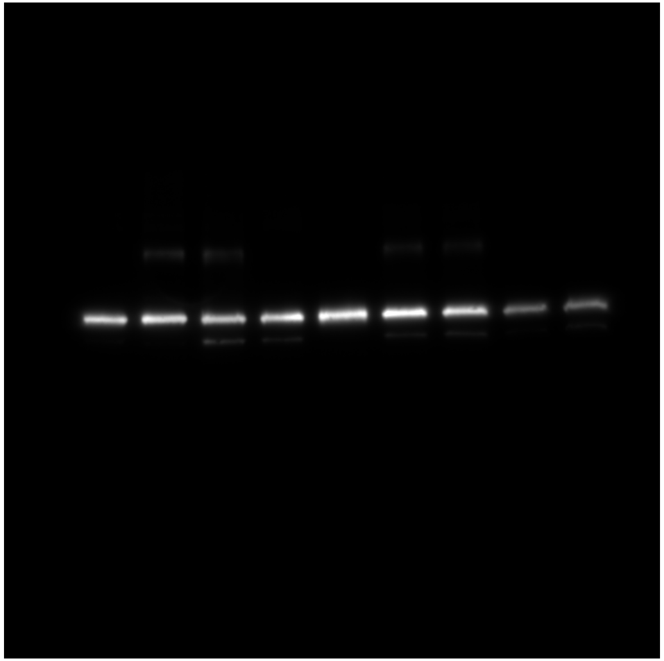

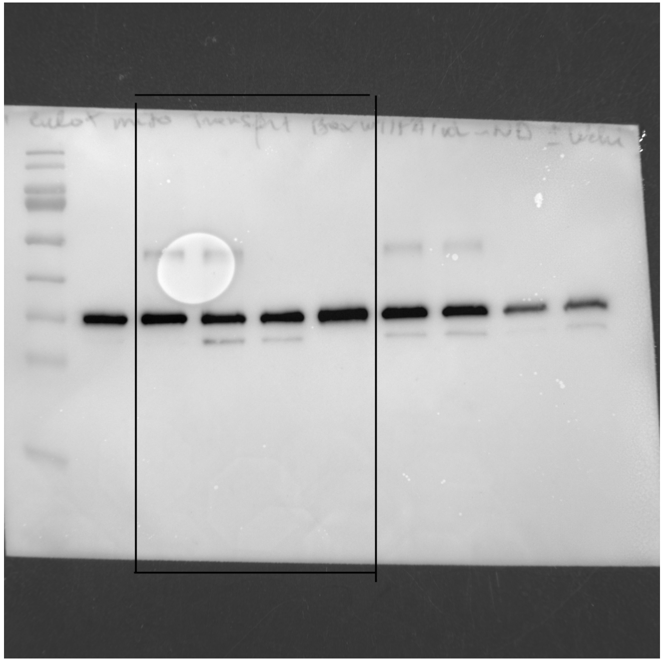

(the selection rectangle shows the lane used for the Figure. The 4 outside lanes on the right were done with
a constitutively active Bax mutant, and the outside lane on the left was done with purified Bax (wihout nanodiscs).
These were omitted for the clarity of the experiment.

Fig4A_20230801

Bax

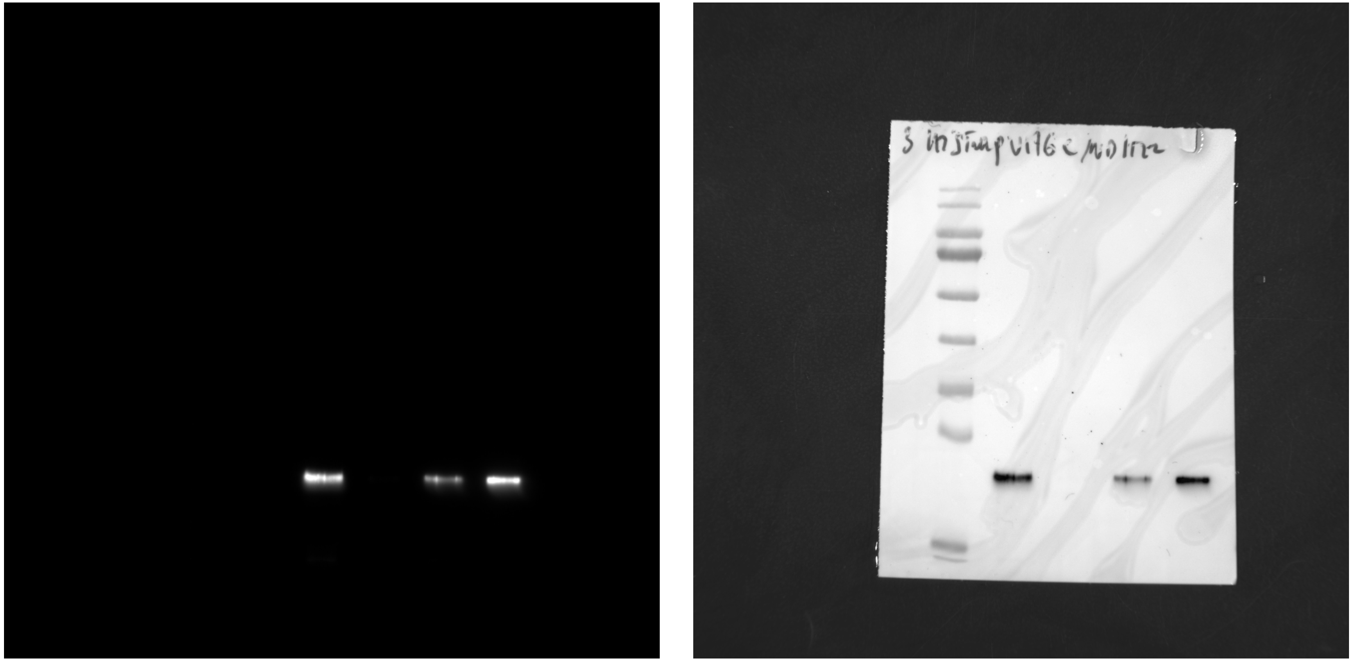

Fig4A_20230801

His6

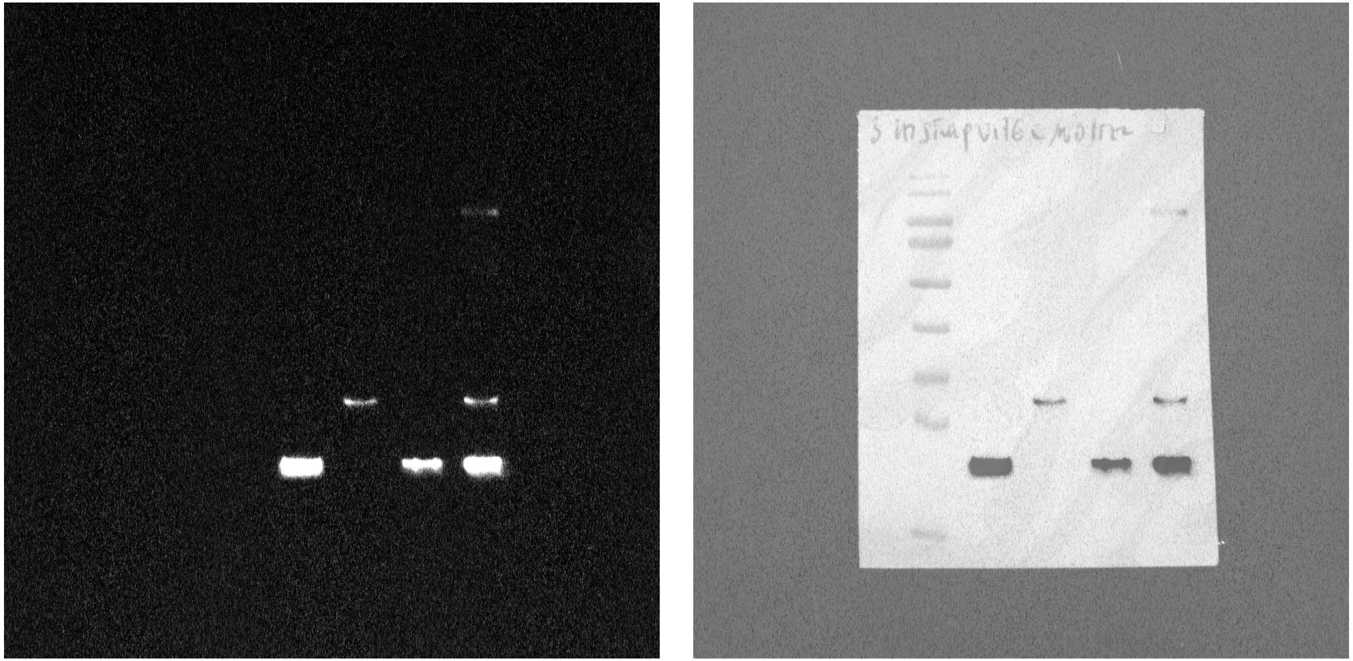

Fig4A_20230801

Tom22

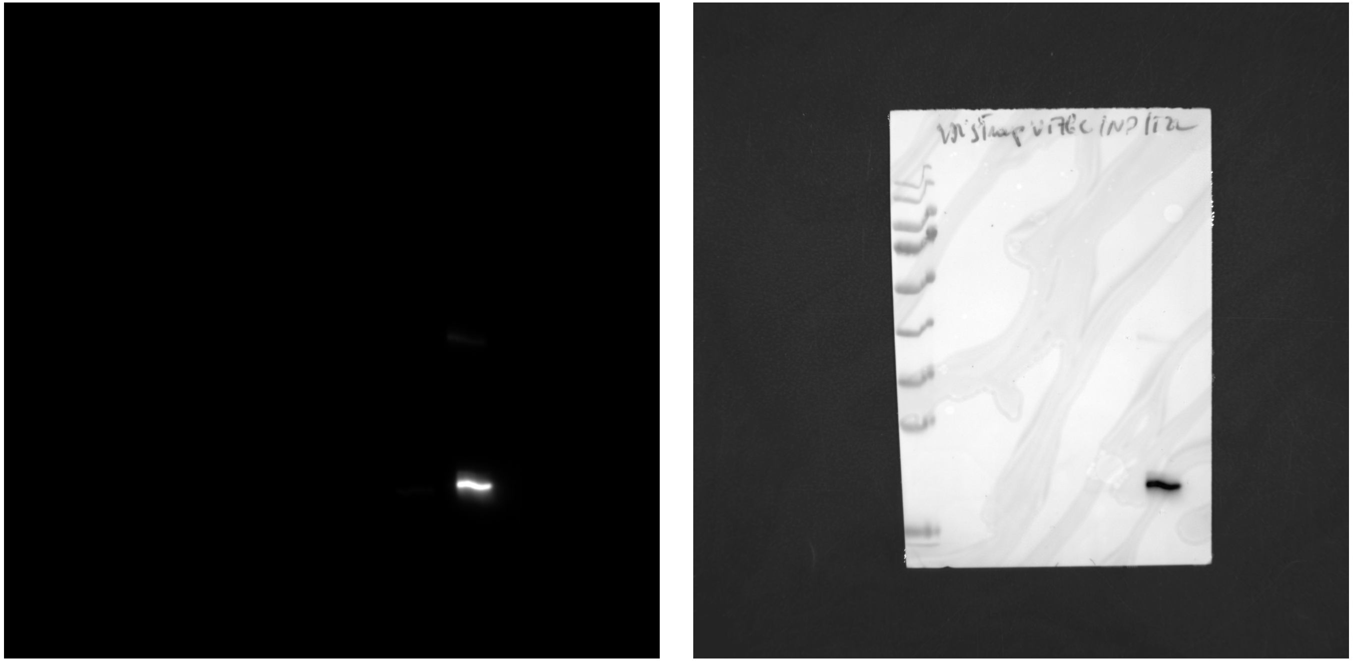

Fig4B_20230801

Bax

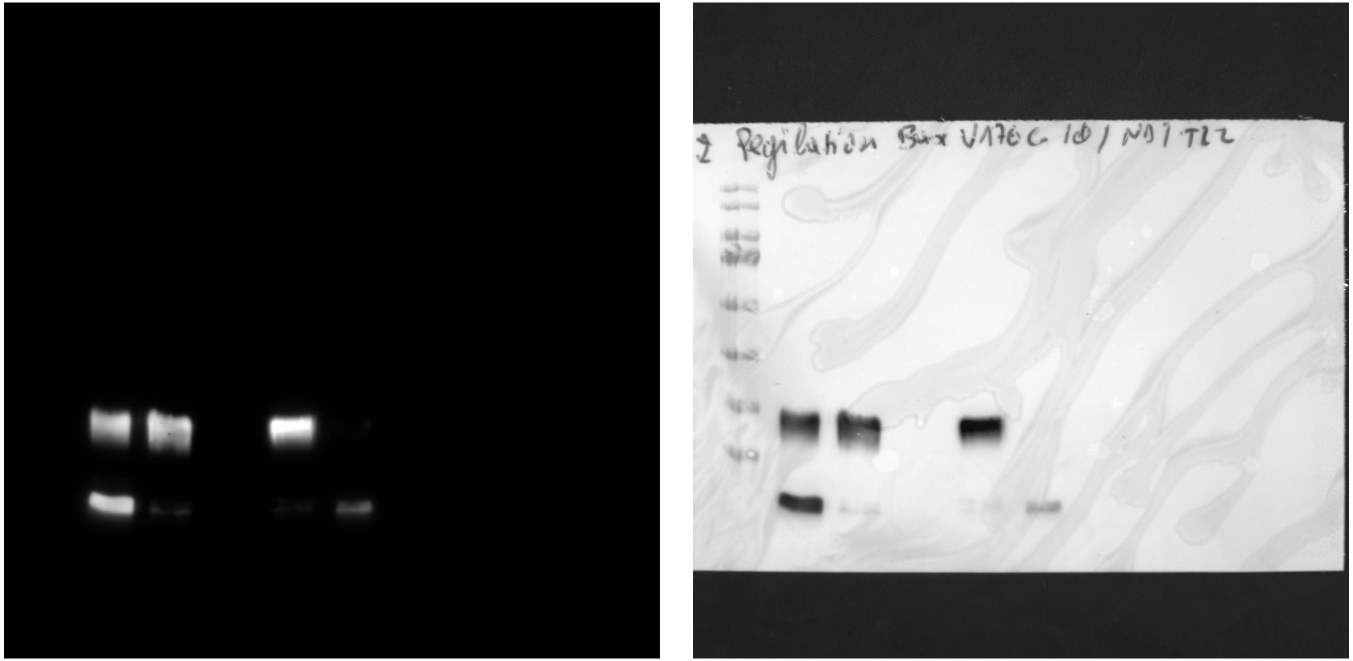

Fig4C_20230515

Bax

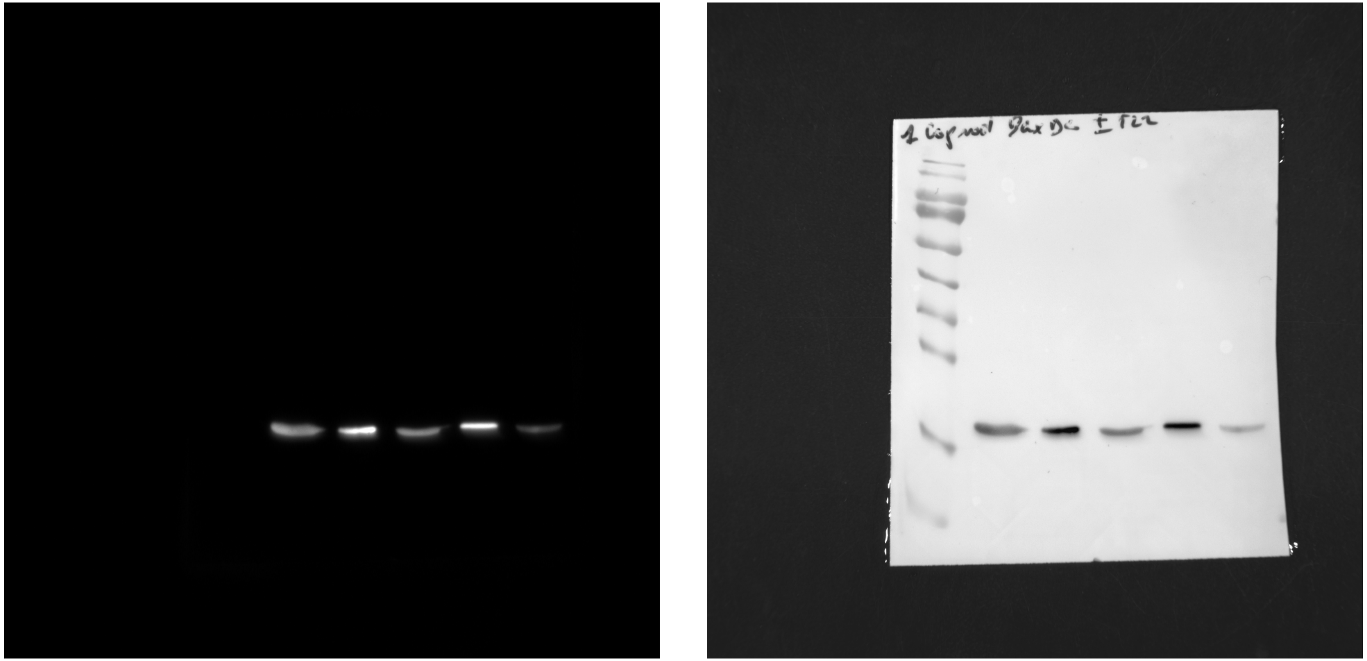

Fig4C_20230515

Tom22

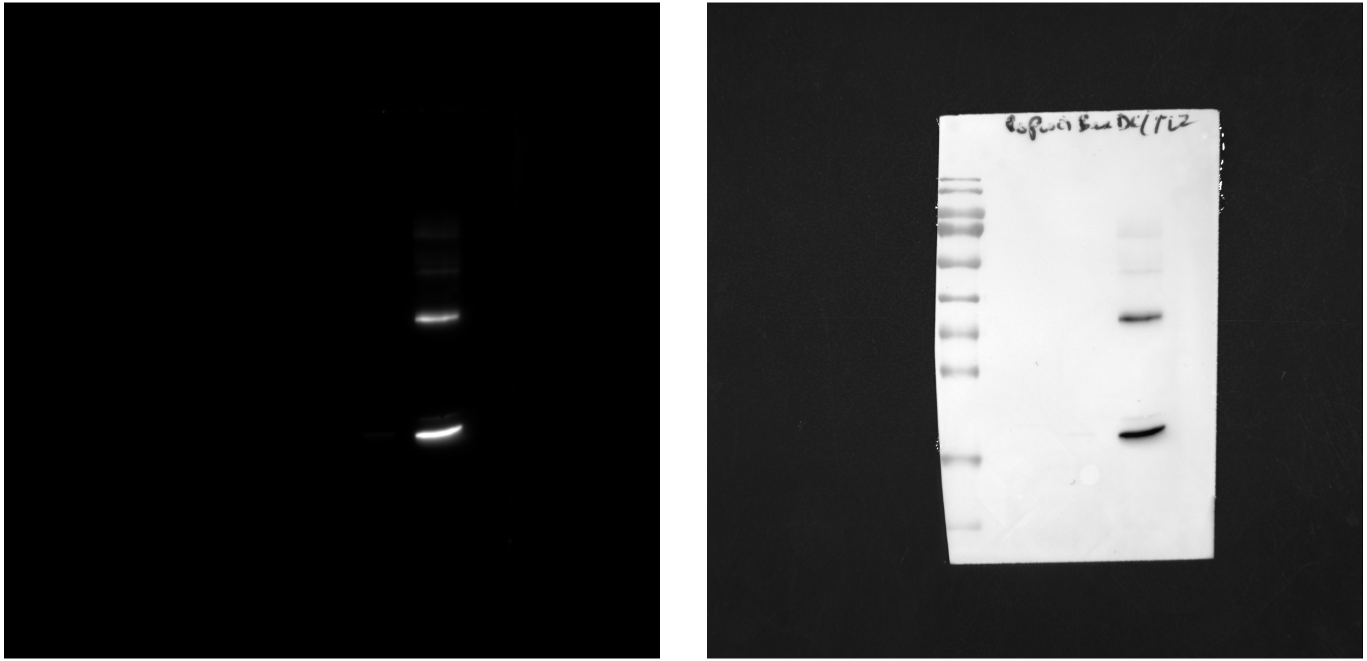

Fig4D_20230515

Bax

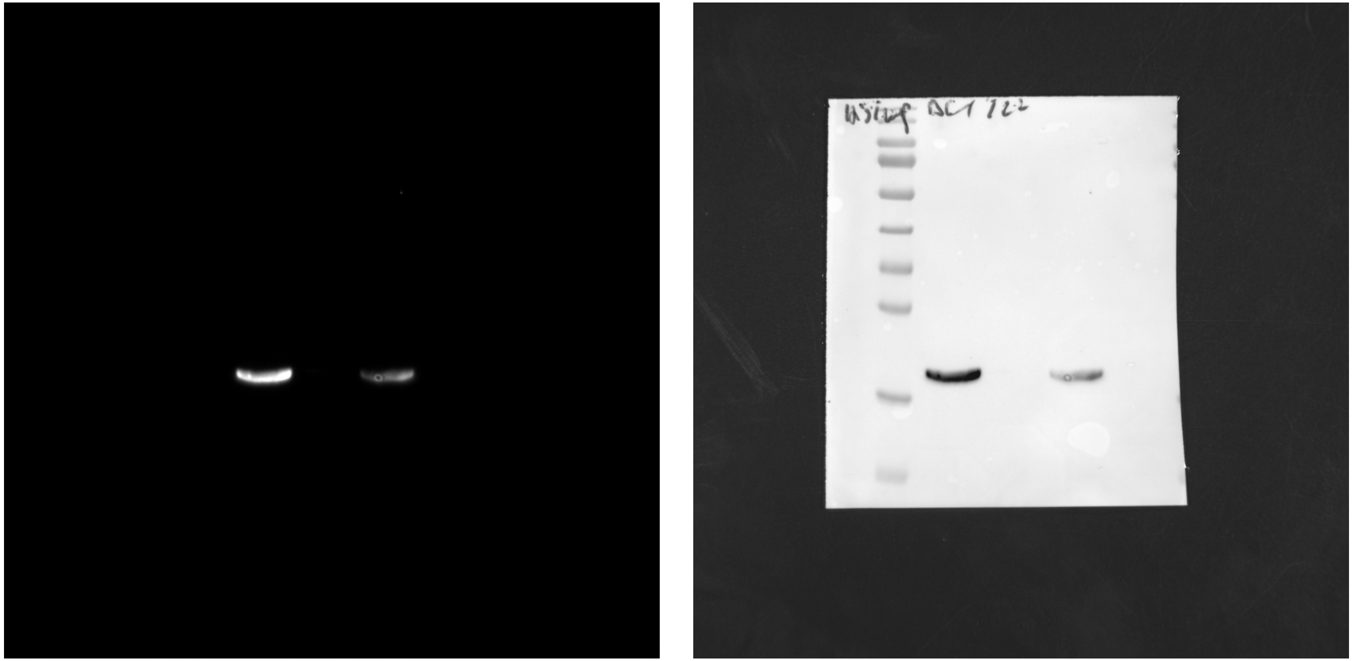

Fig4D_20230515

Tom22

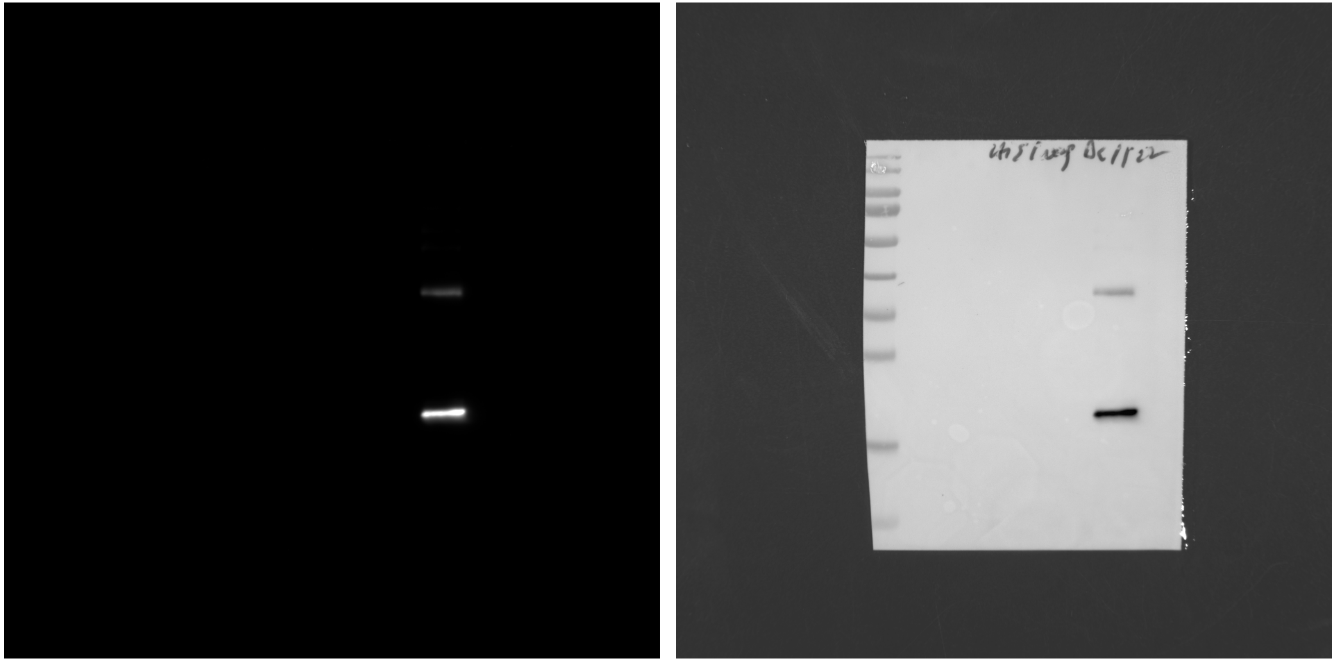

Fig4E_20230628

Bax

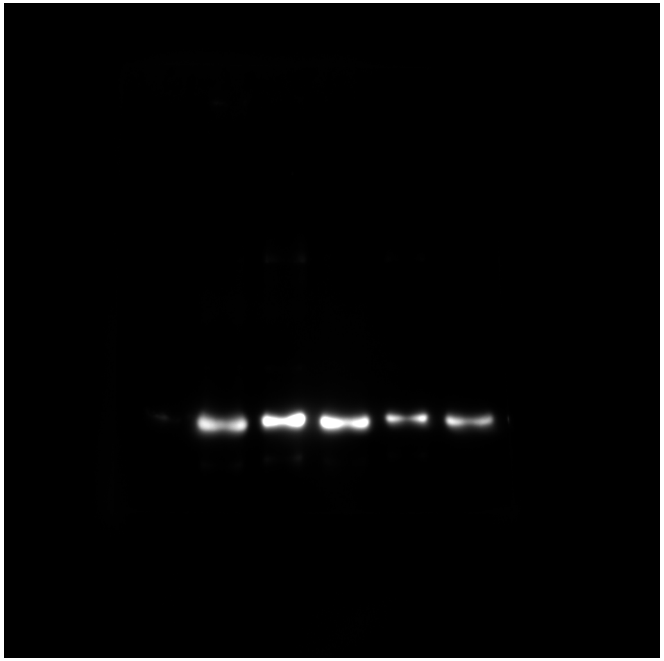

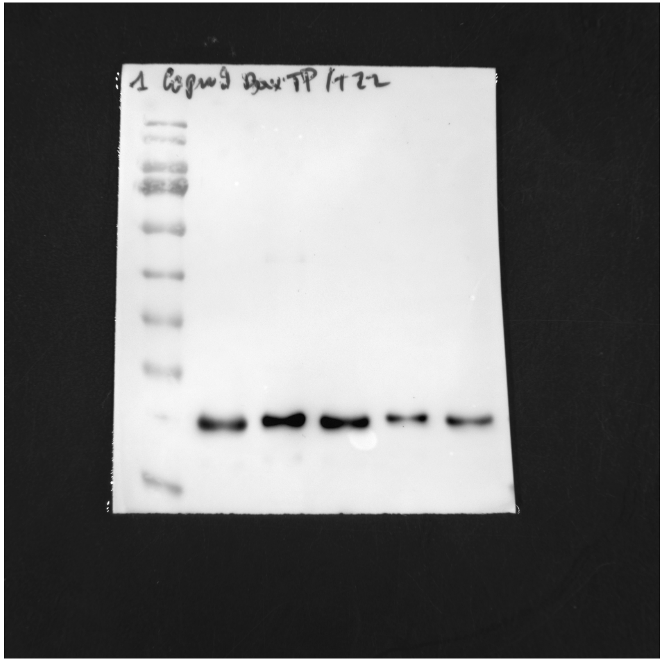

Fig4E_20230628

Tom22

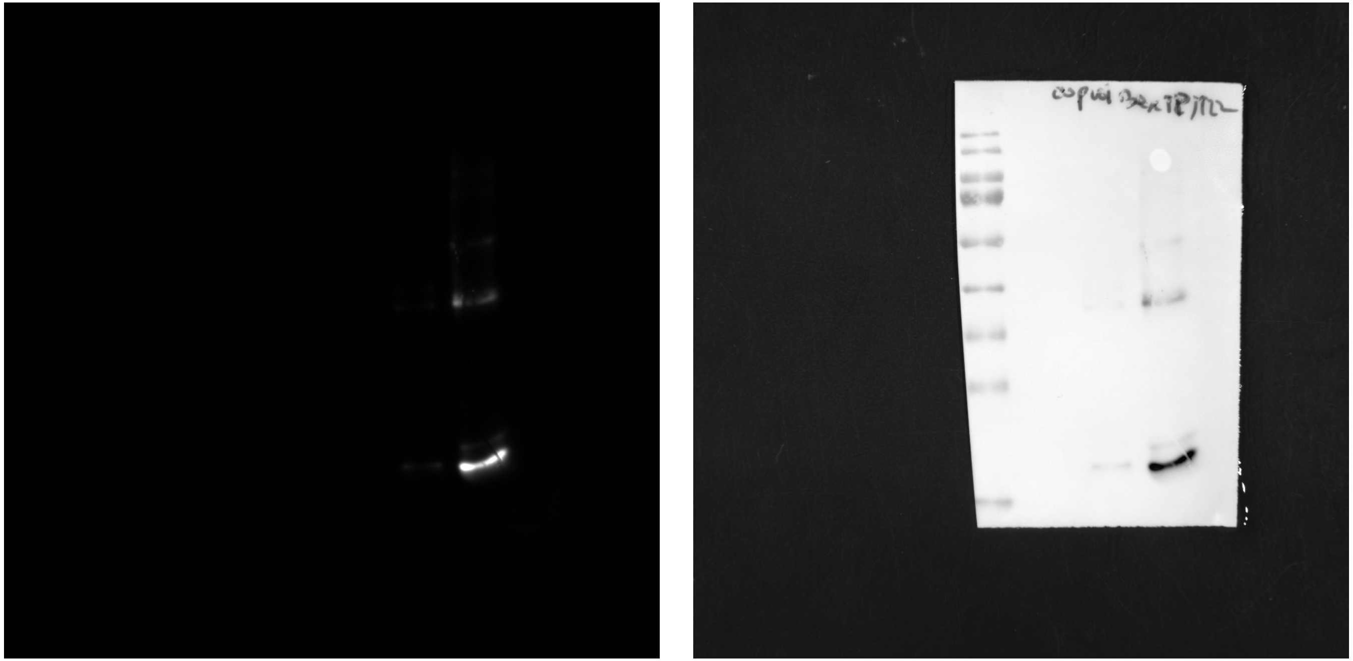

Fig4F_20230628

Bax

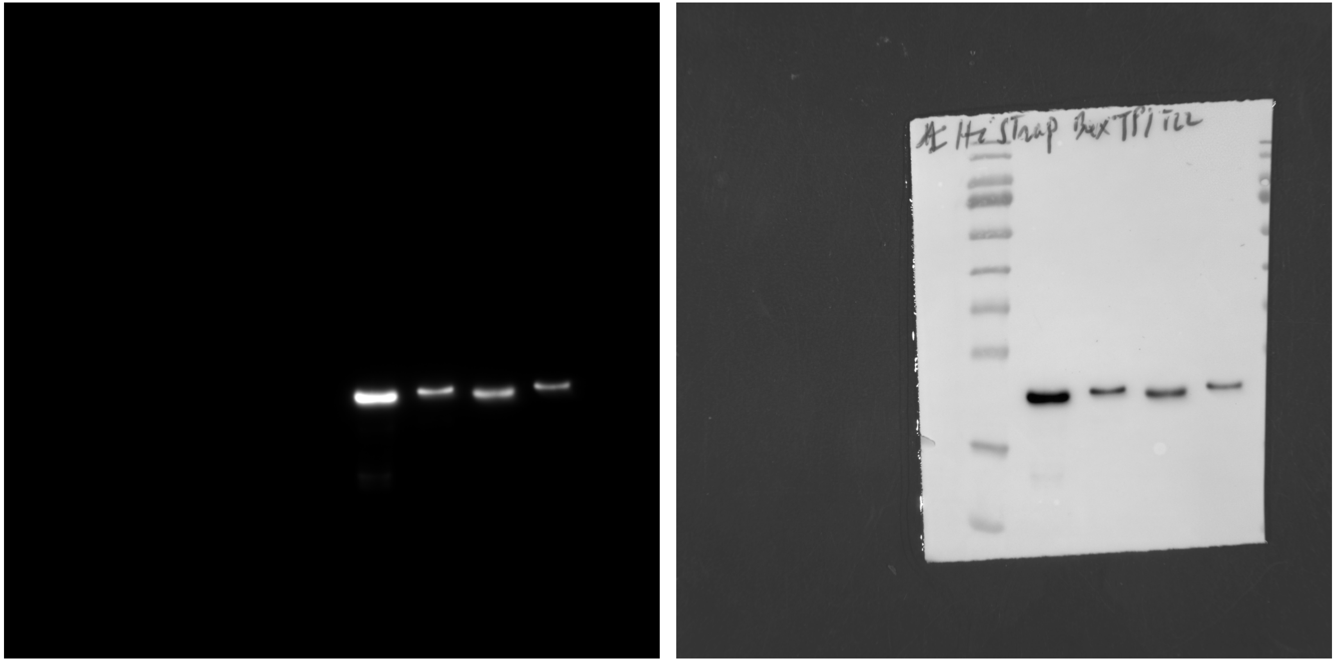

Fig4F_20230628

His6

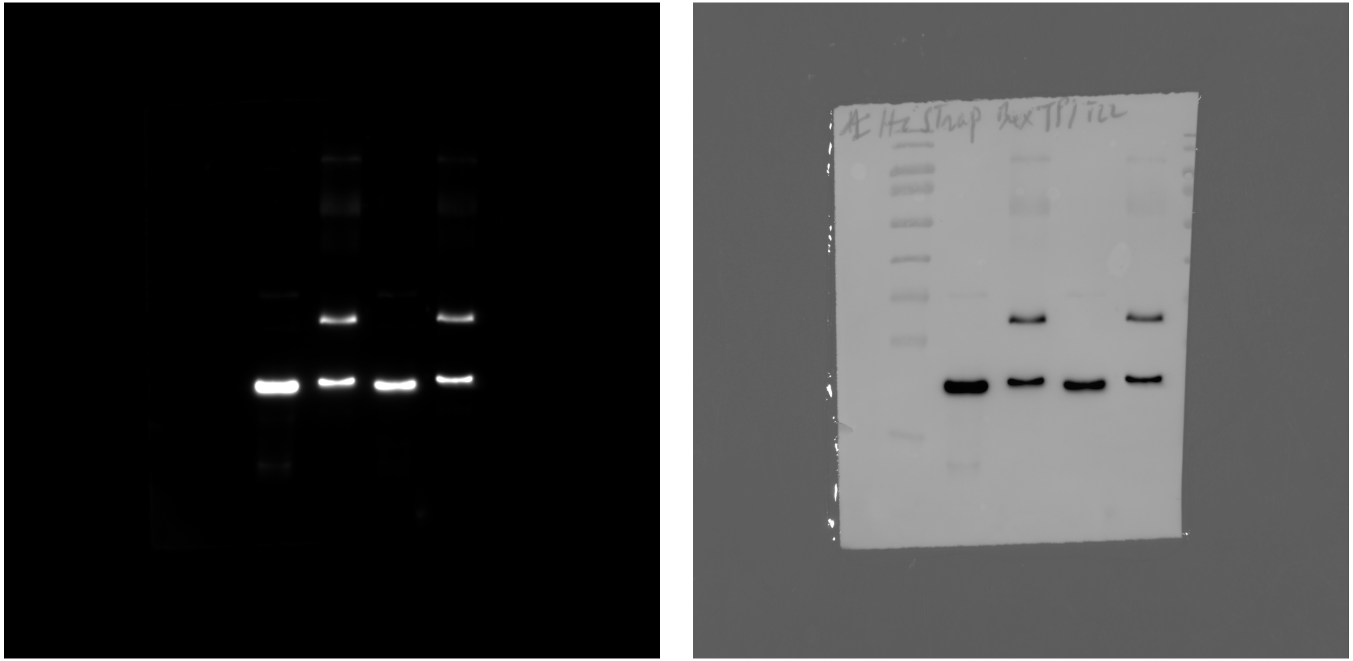

Fig4F_20230628

Tom22

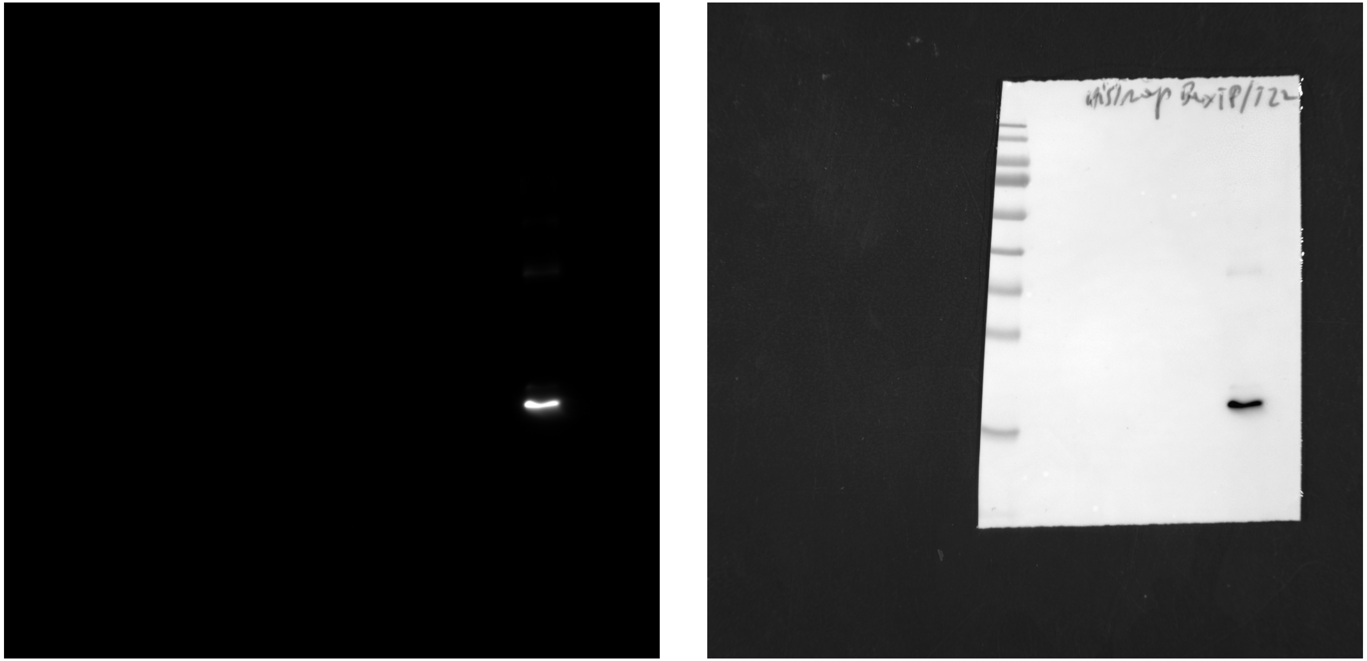

Fig5A_20230320

Bax

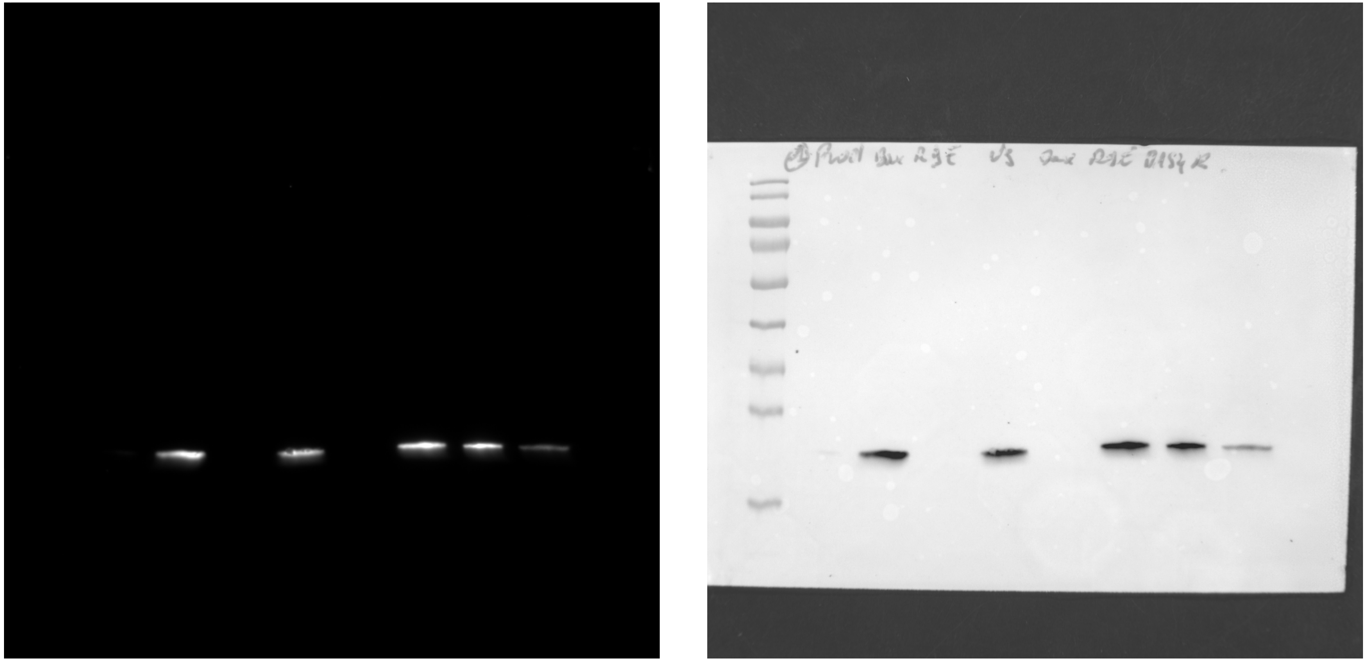

Fig5B_20230320

Bax

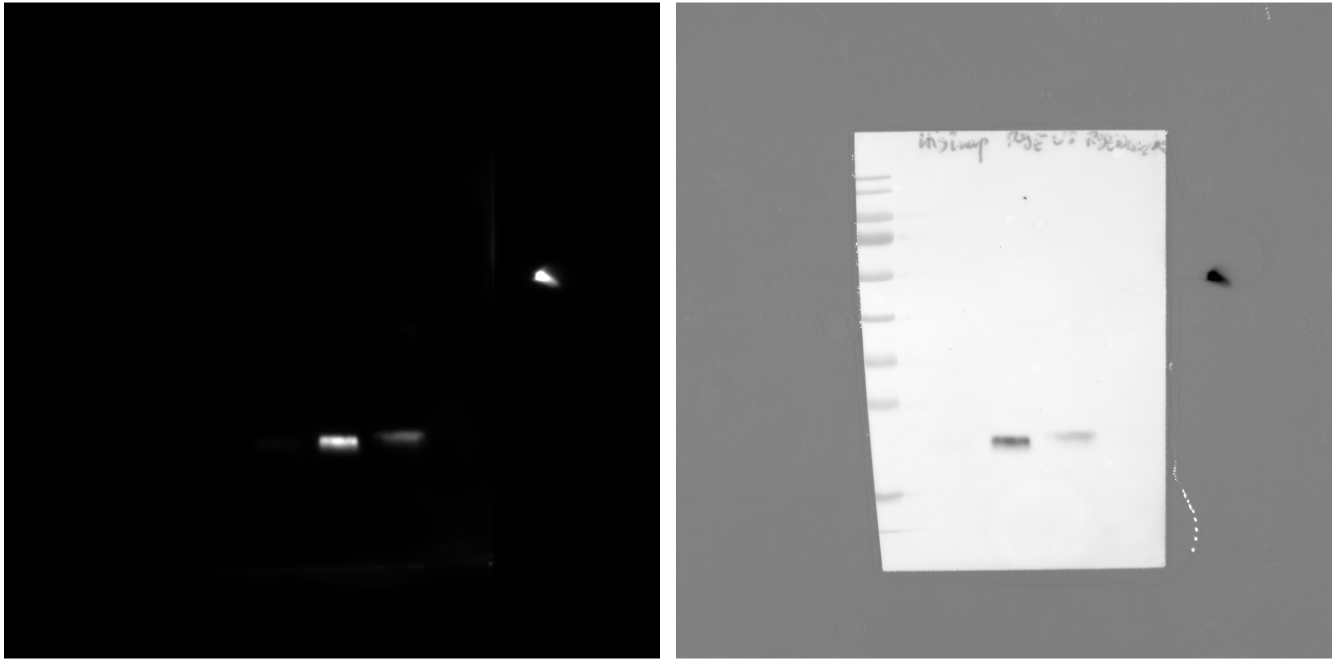

Fig5B_20230320

His6

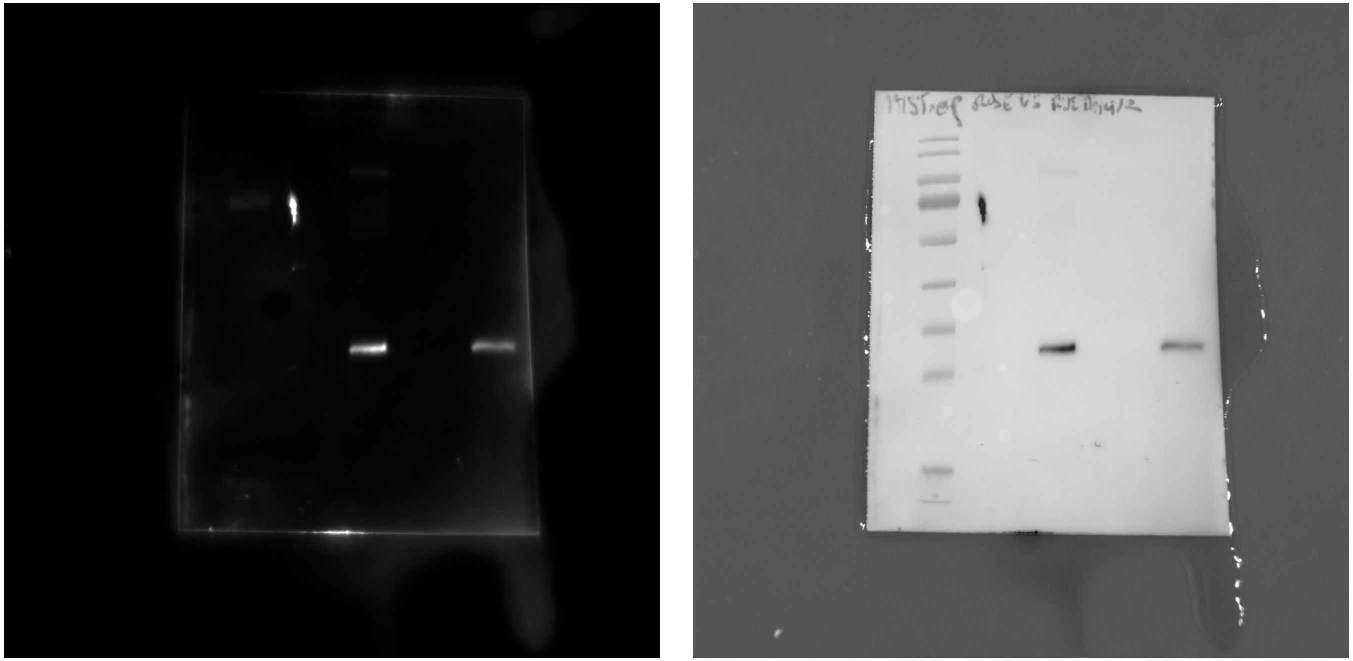

Fig5C_20230816

Bax

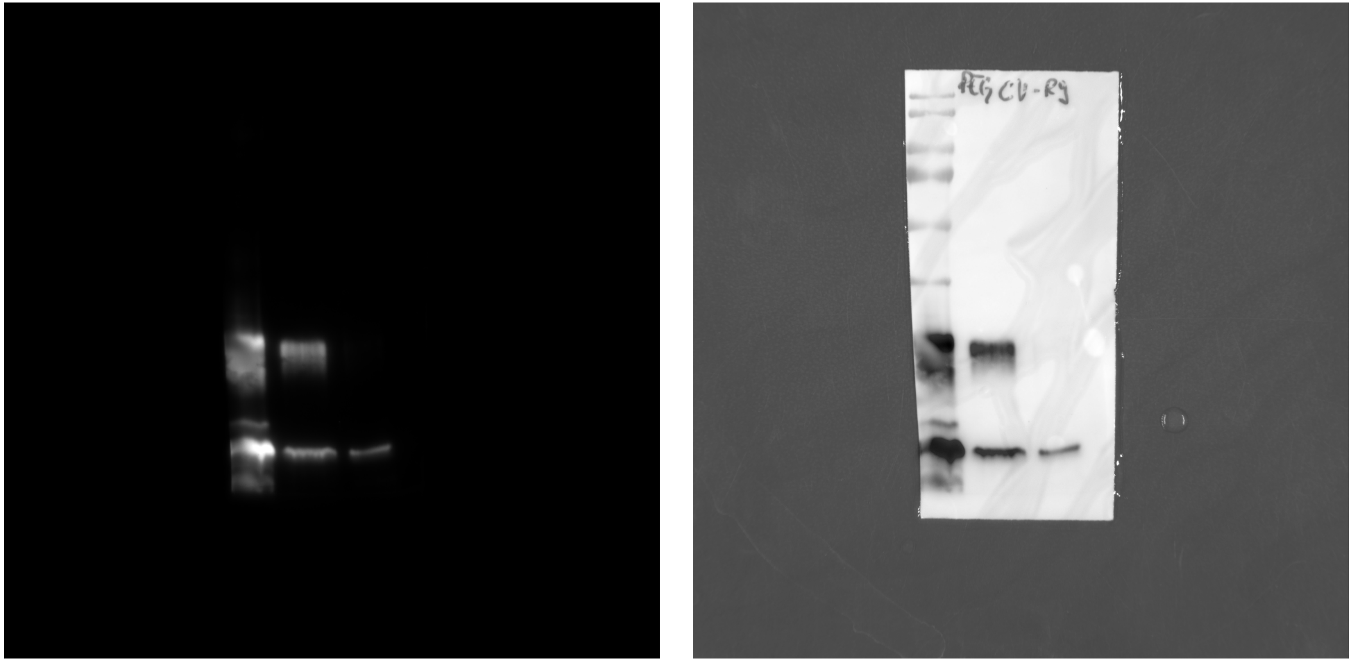

Fig5D_20230628

Bax

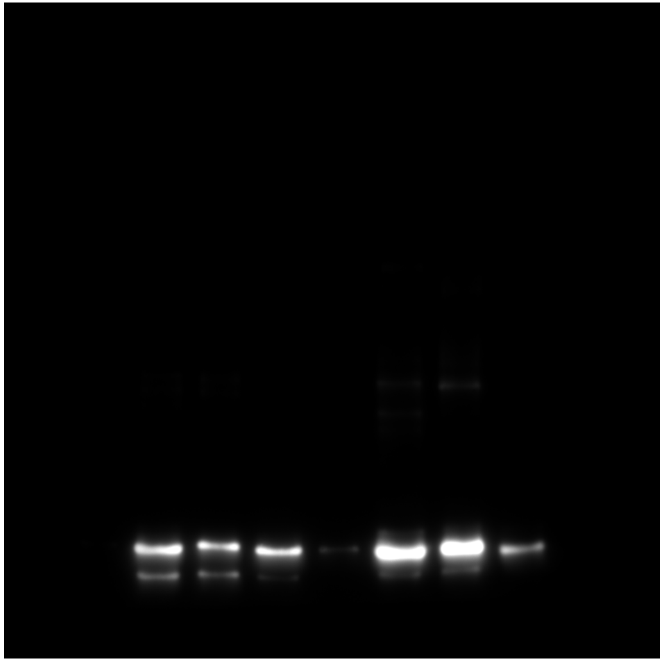

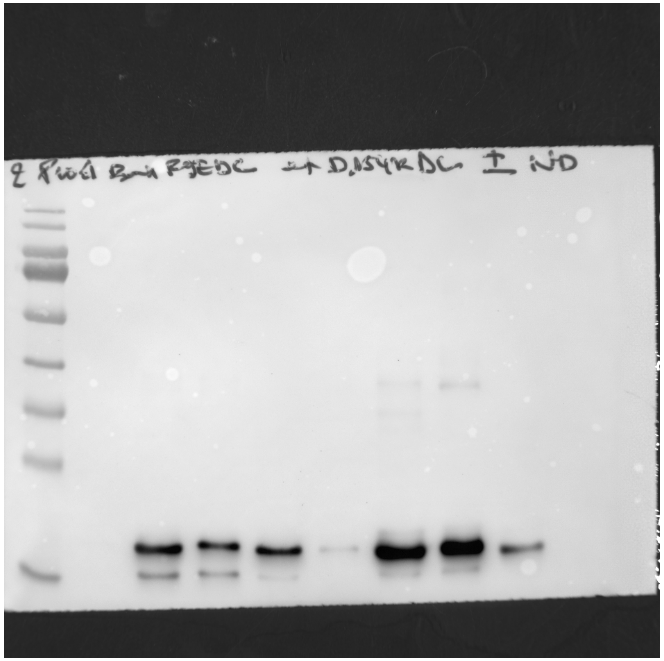

Fig5E_20230628

Bax

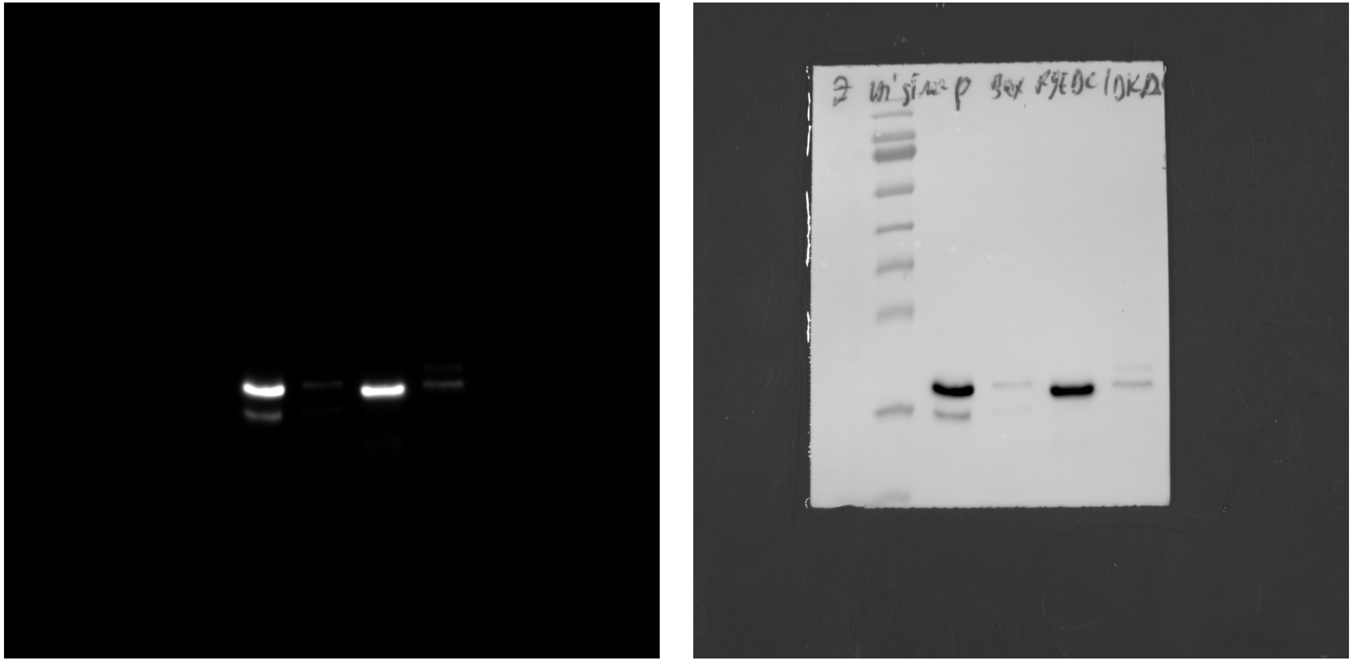

Fig5E_20230628

His6

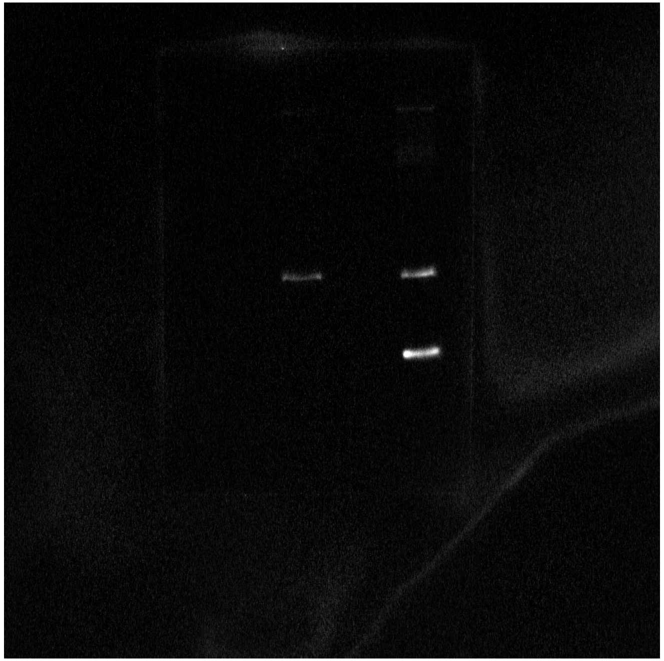

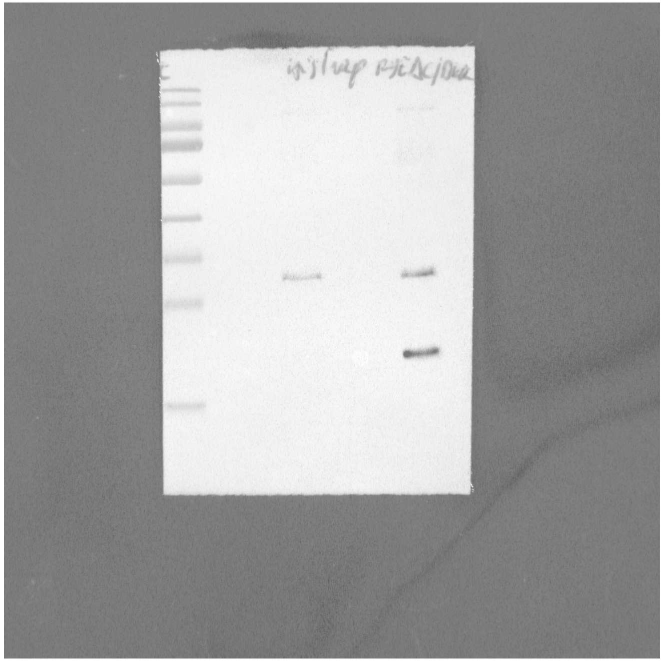

Fig5F_20230808

Bax

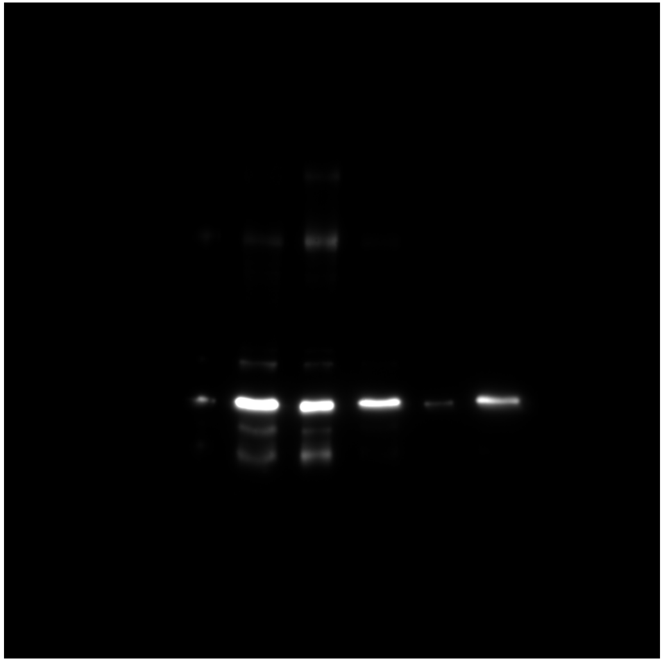

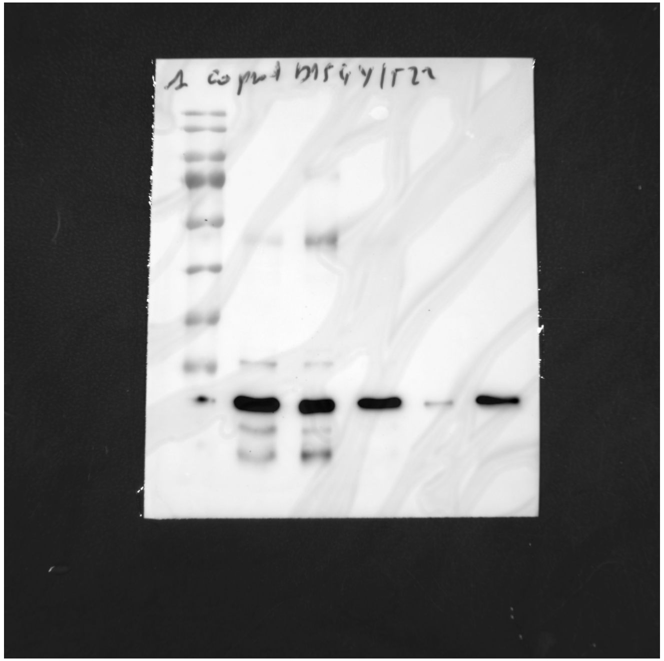

Fig5F_20230808

Tom22

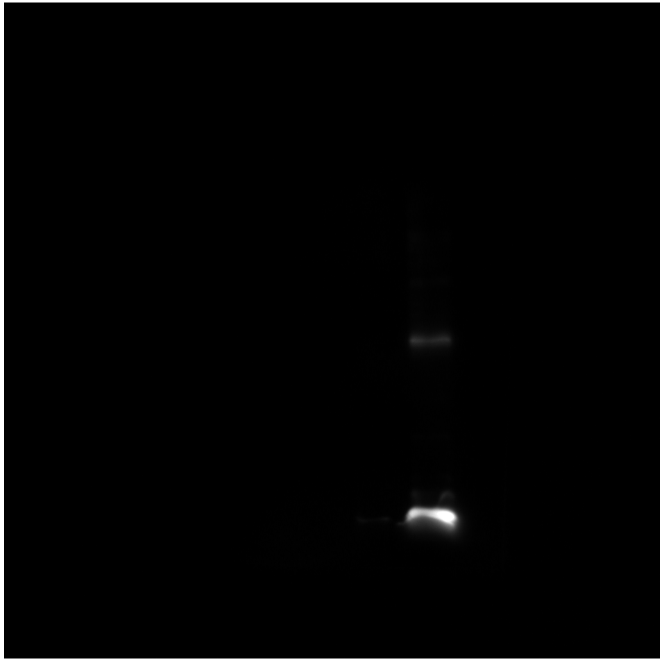

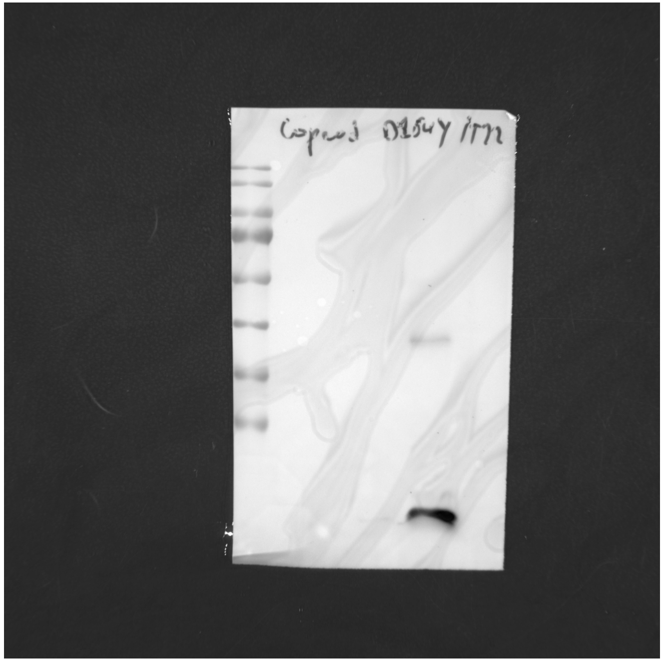

Fig5G_20230808

Bax

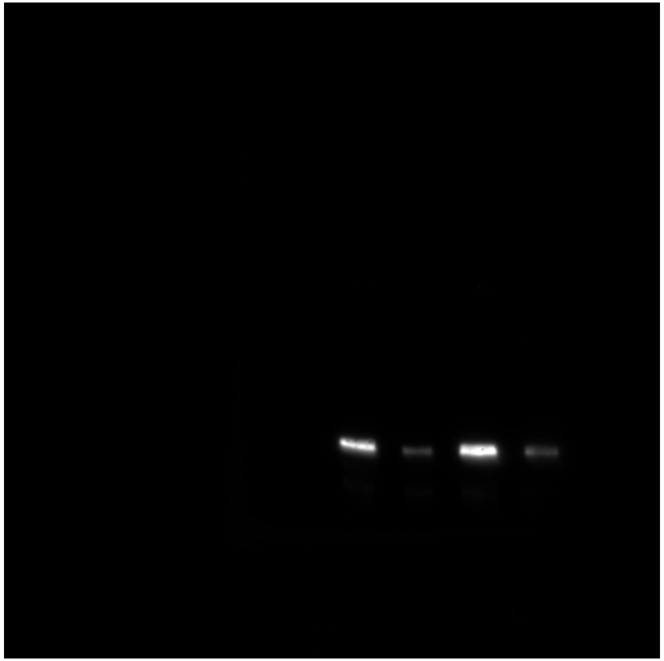

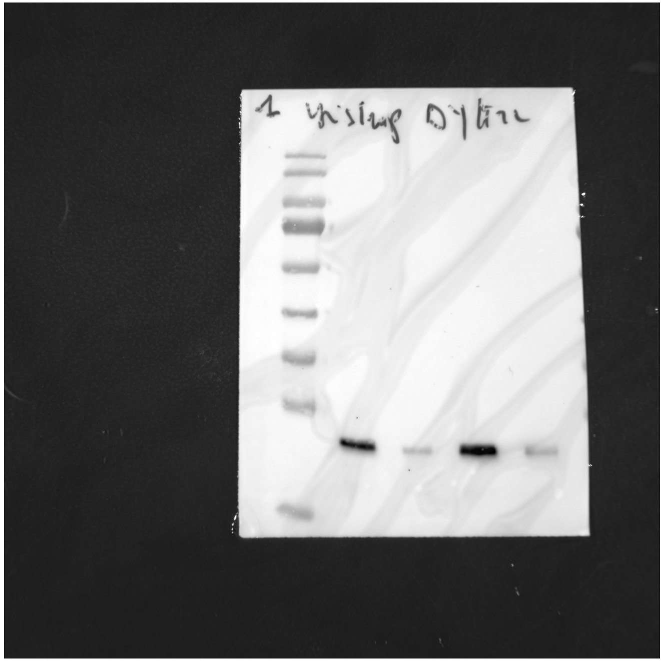

Fig5G_20230808

His6

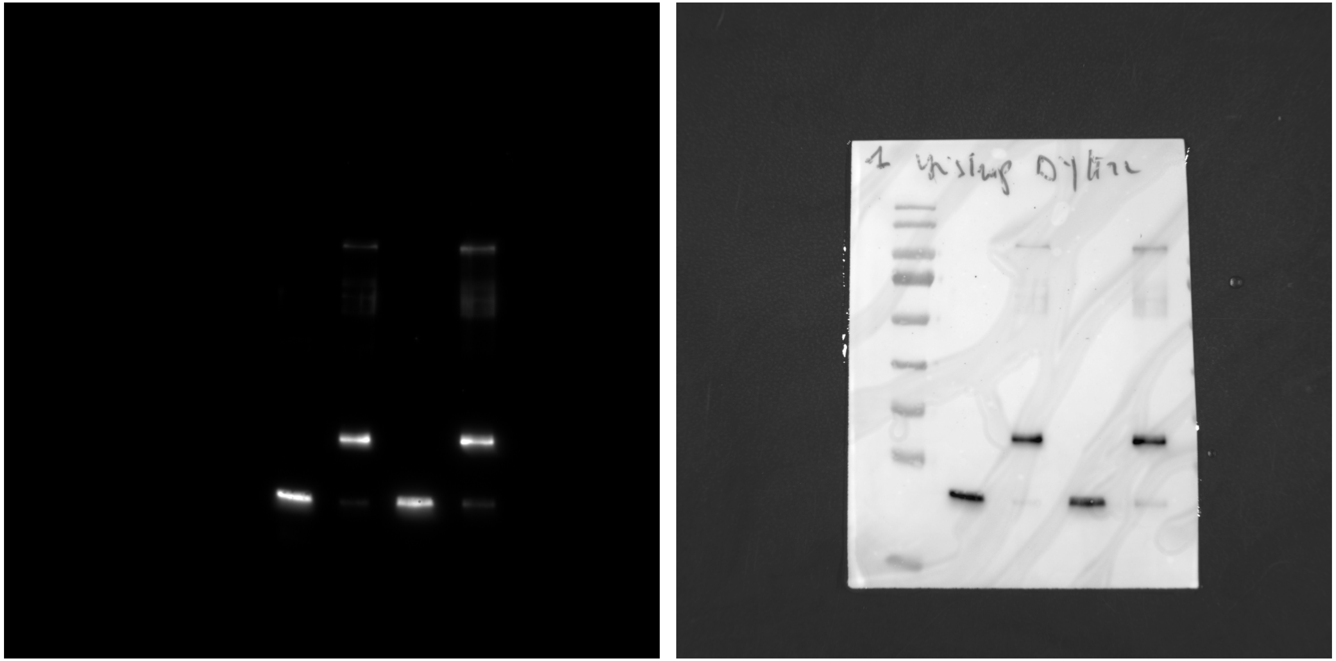

Fig5G_20230808

Tom22

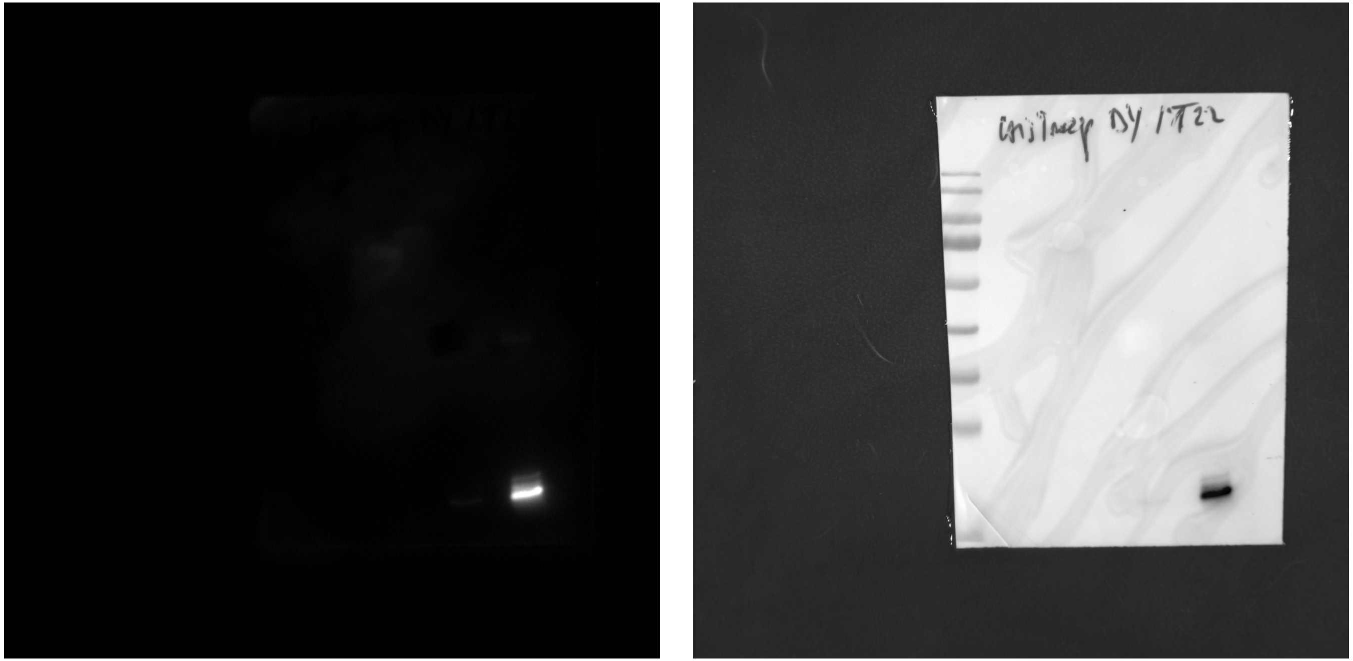

FigS2_20230419

Bax

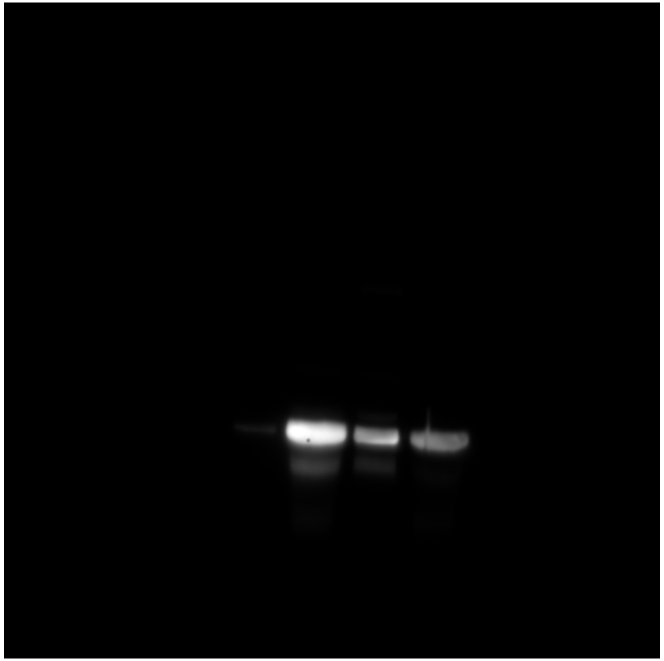

FigS3B_20230406

his6

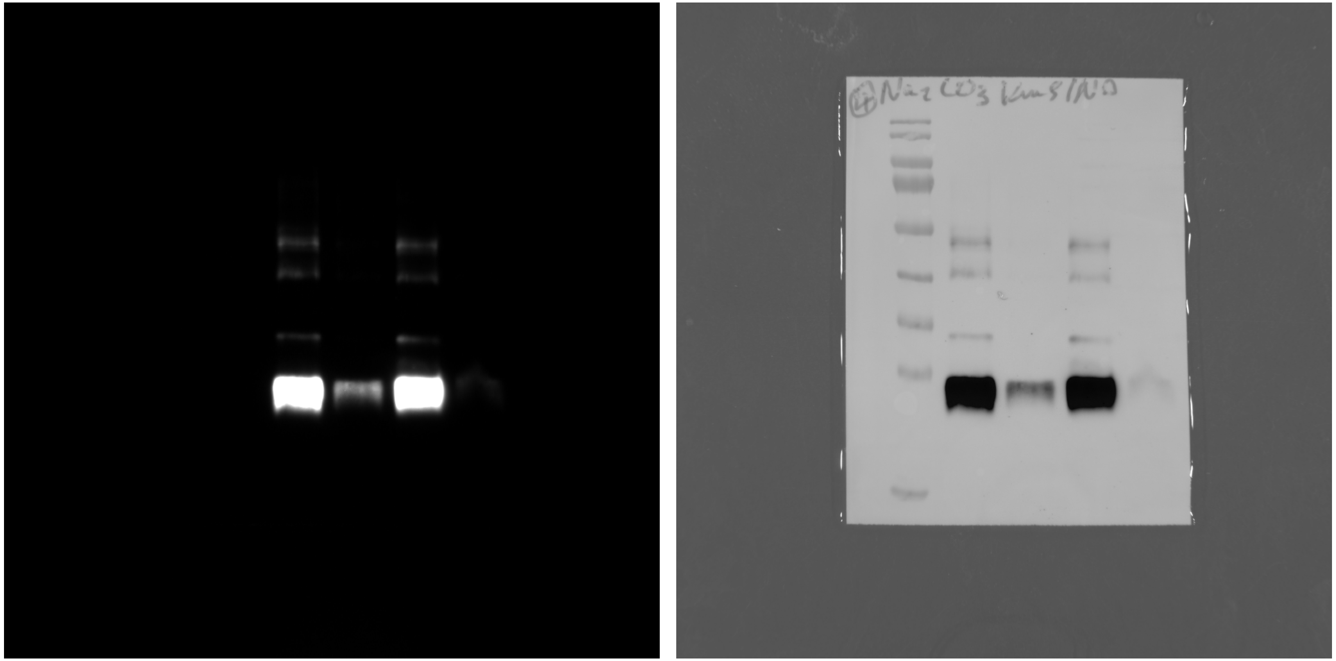

FigS3B_20230406

StrepII

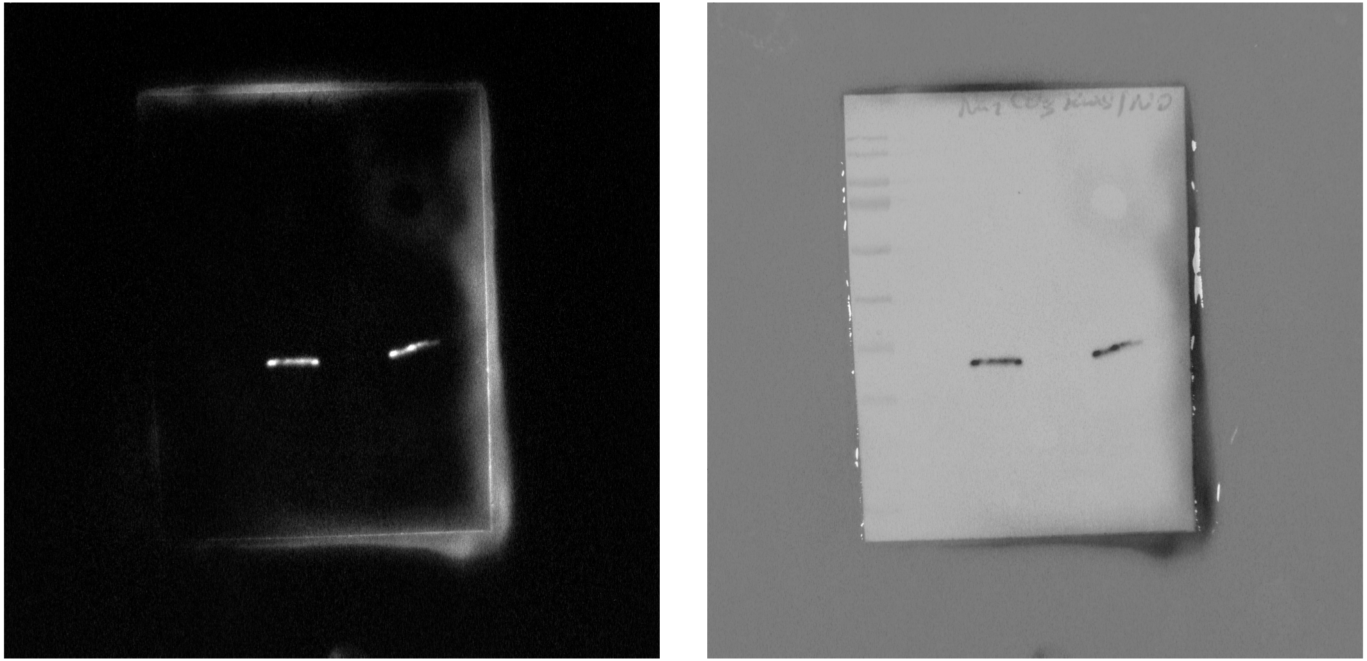

FigS3C_20230321

Bax

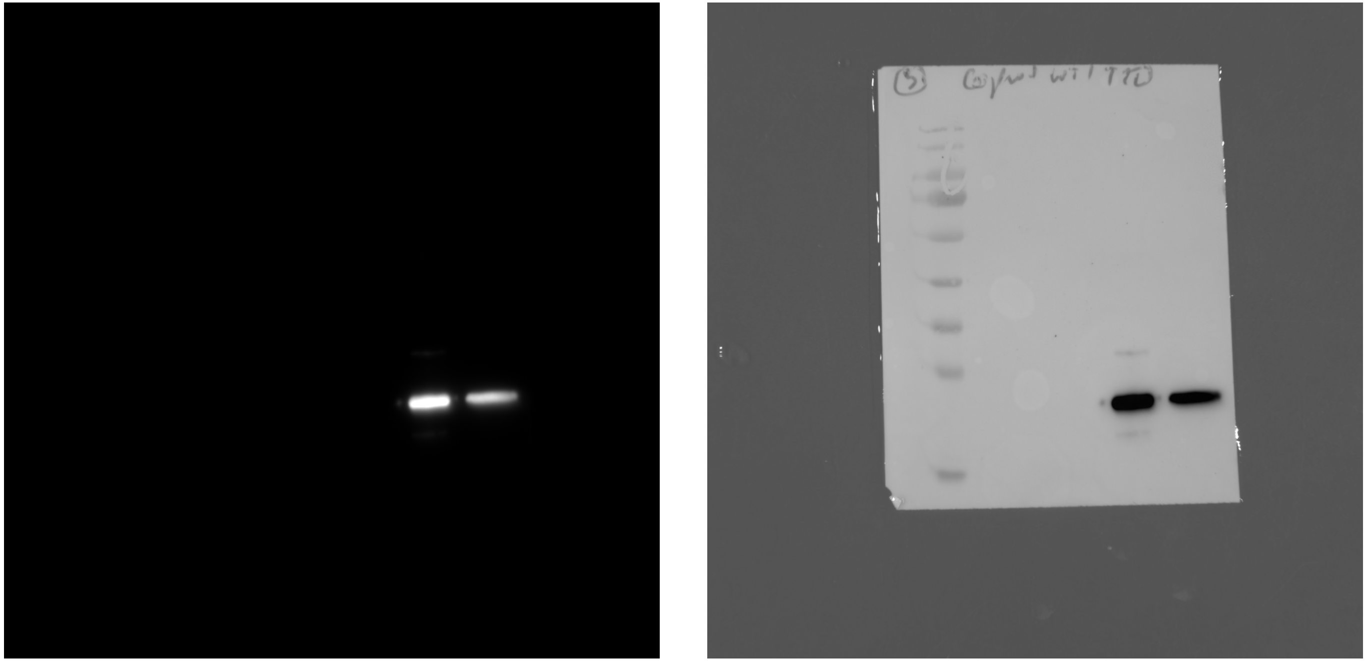

FigS3C_20230321

tom20

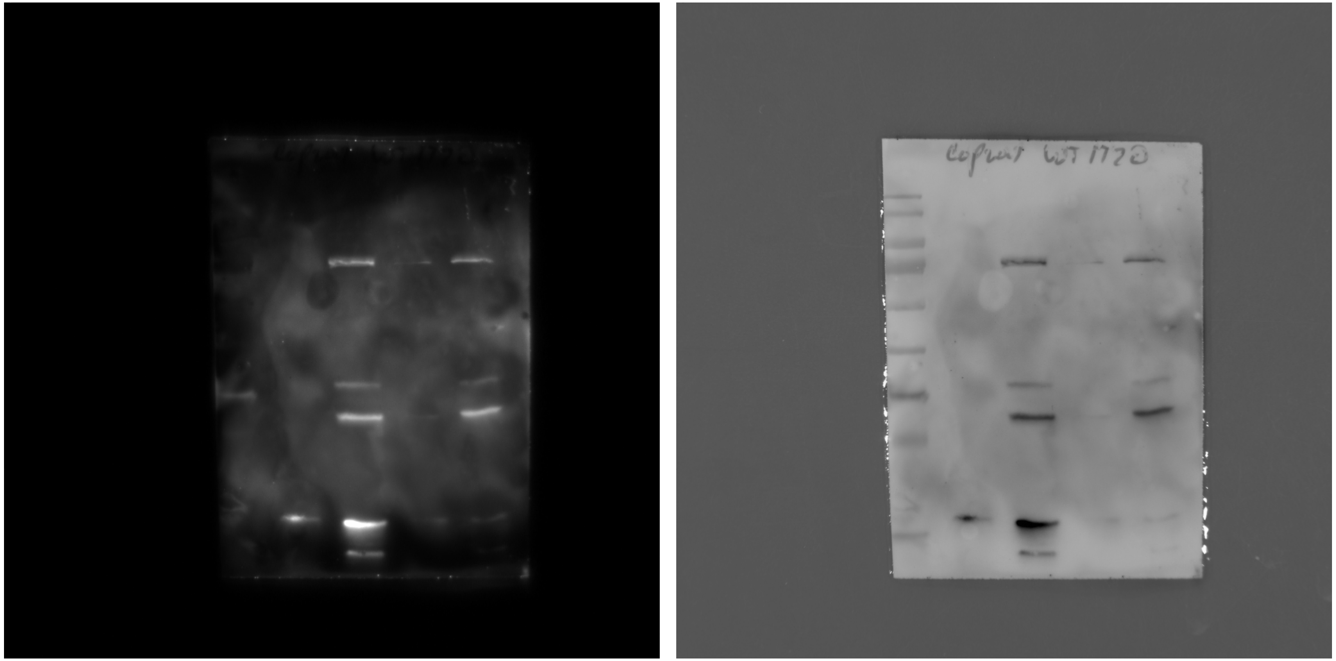

FigS3D_20230323

Bax

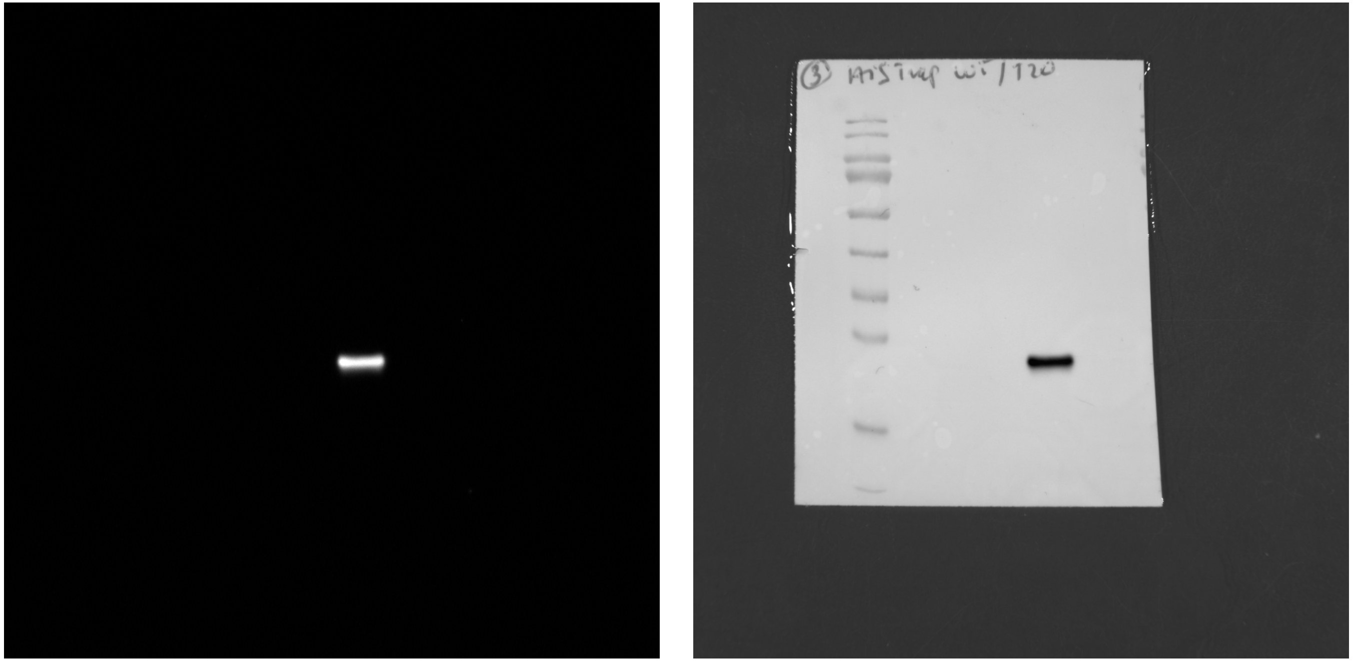

FigS3D_20230323

his6

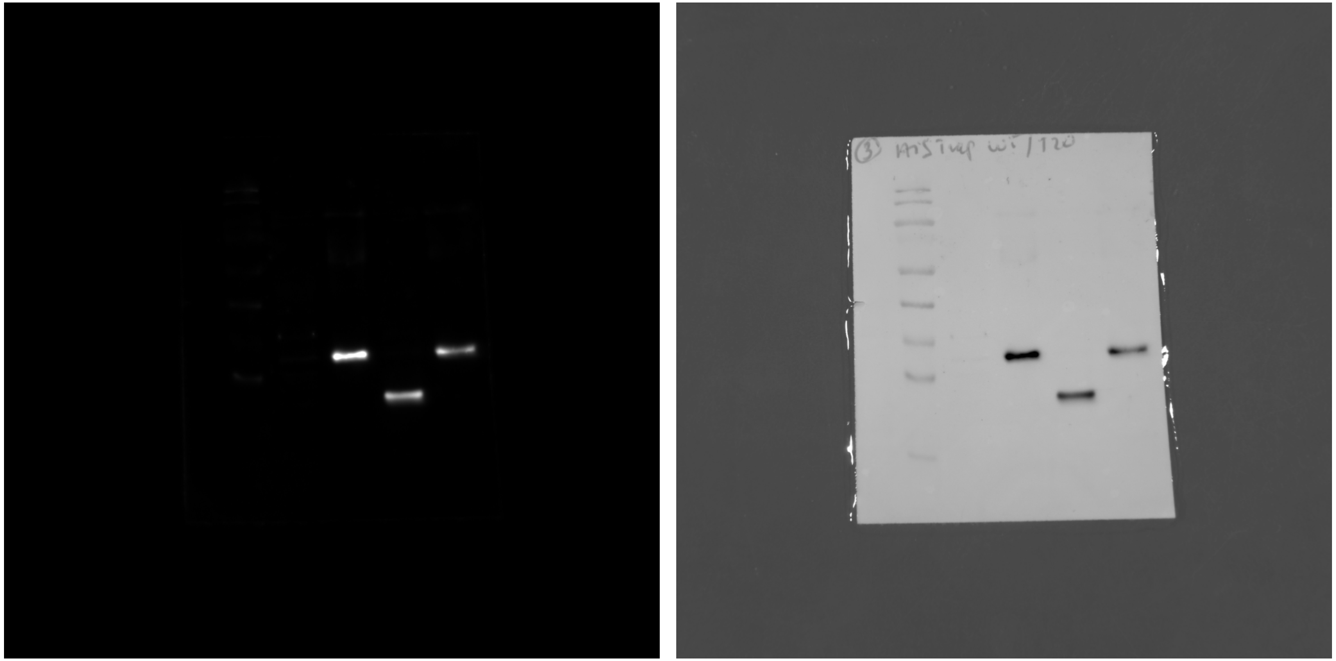

FigS3D_20230323

Tom20

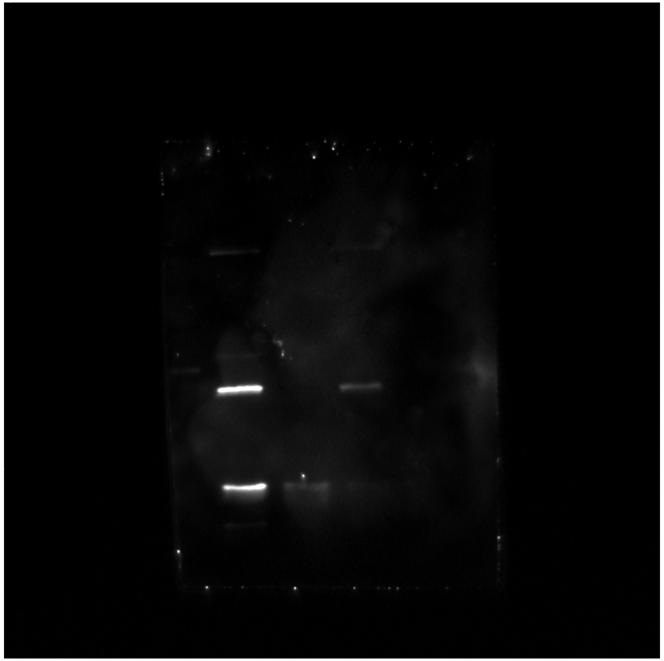

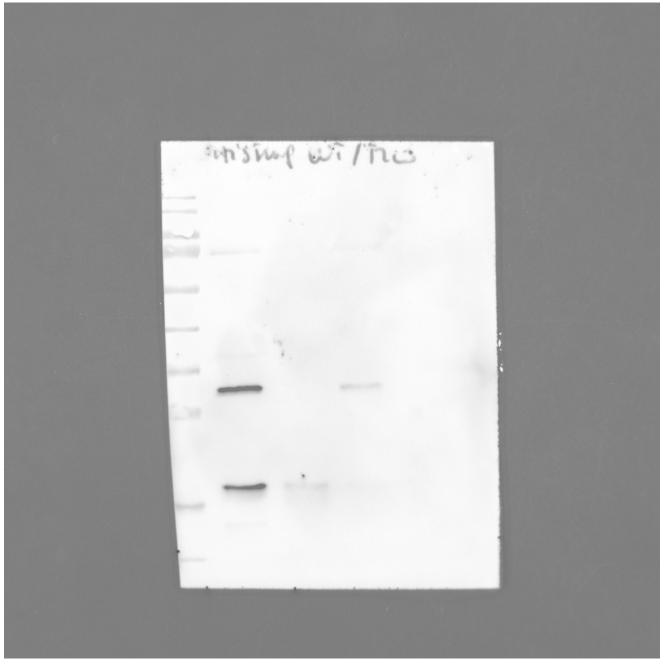

FigS4_20230818

bax

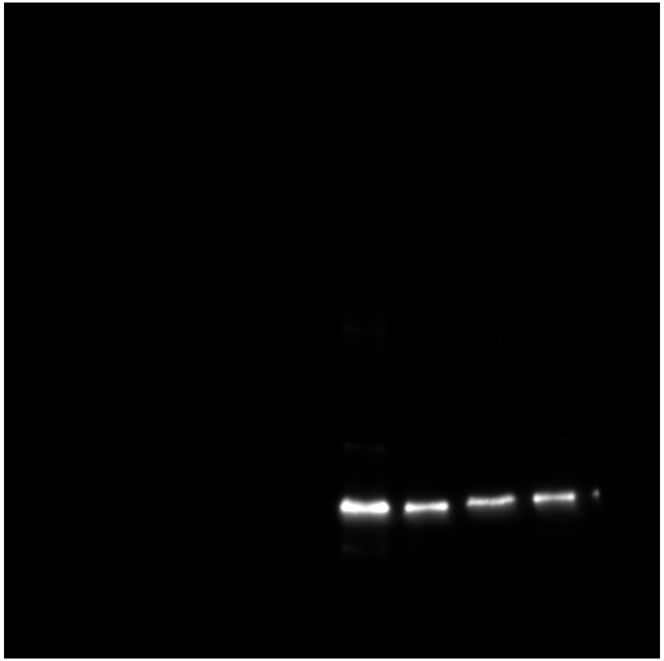

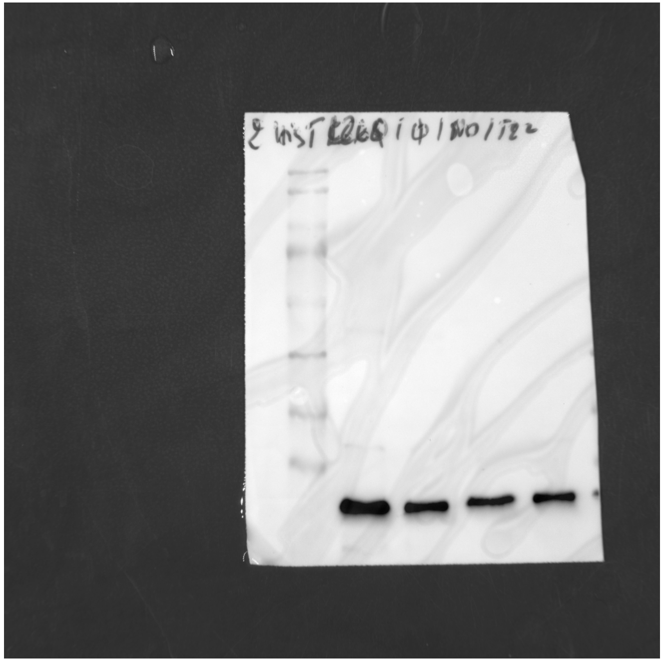

FigS4_20230818

his6

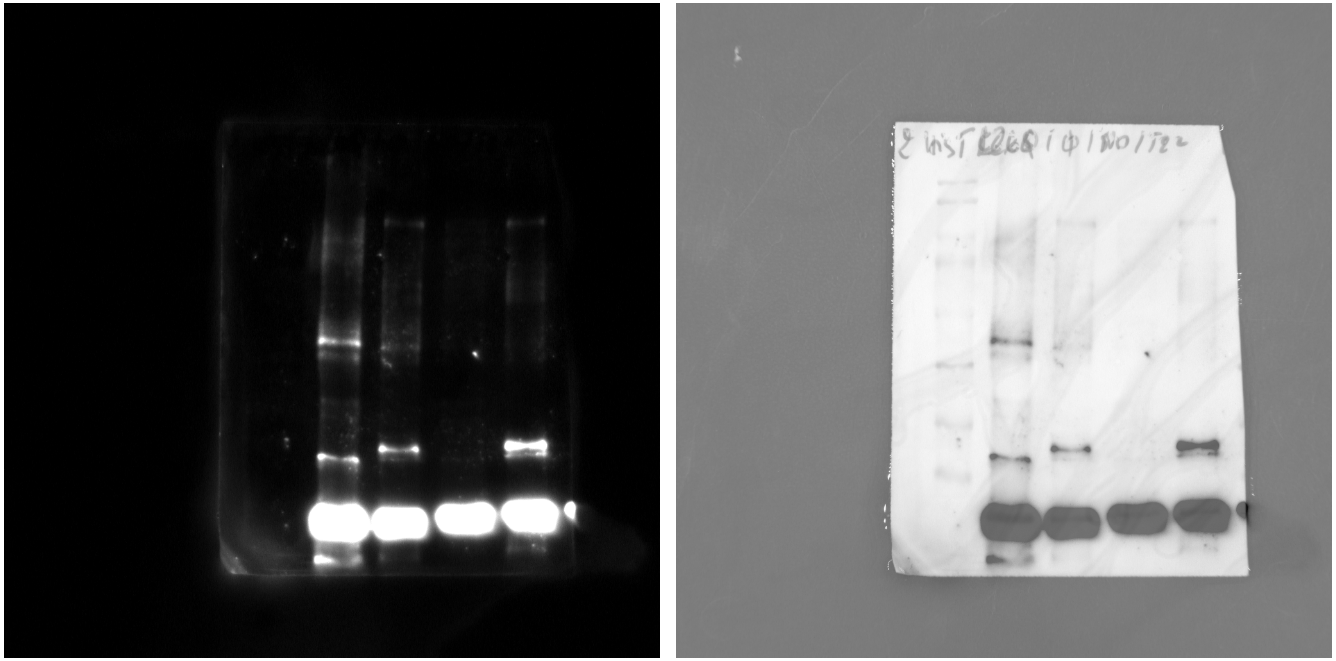

FigS4_20230818

tom22

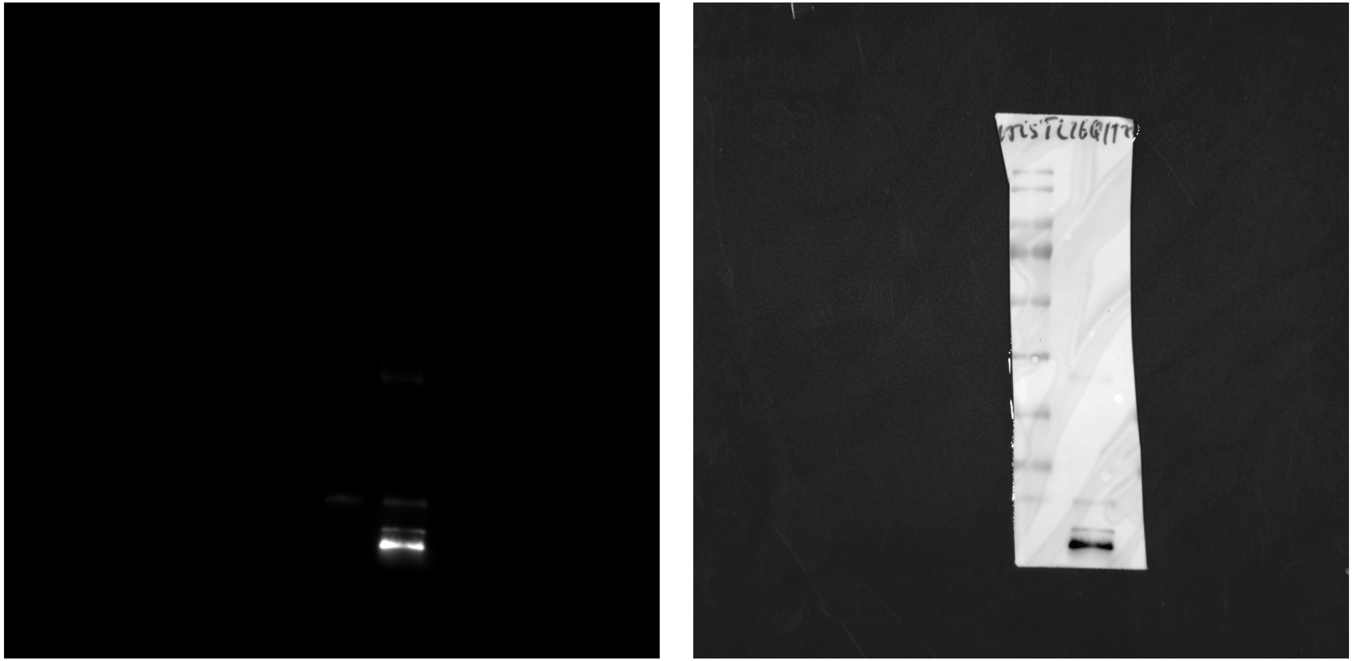

FigS5A_20230808

bax

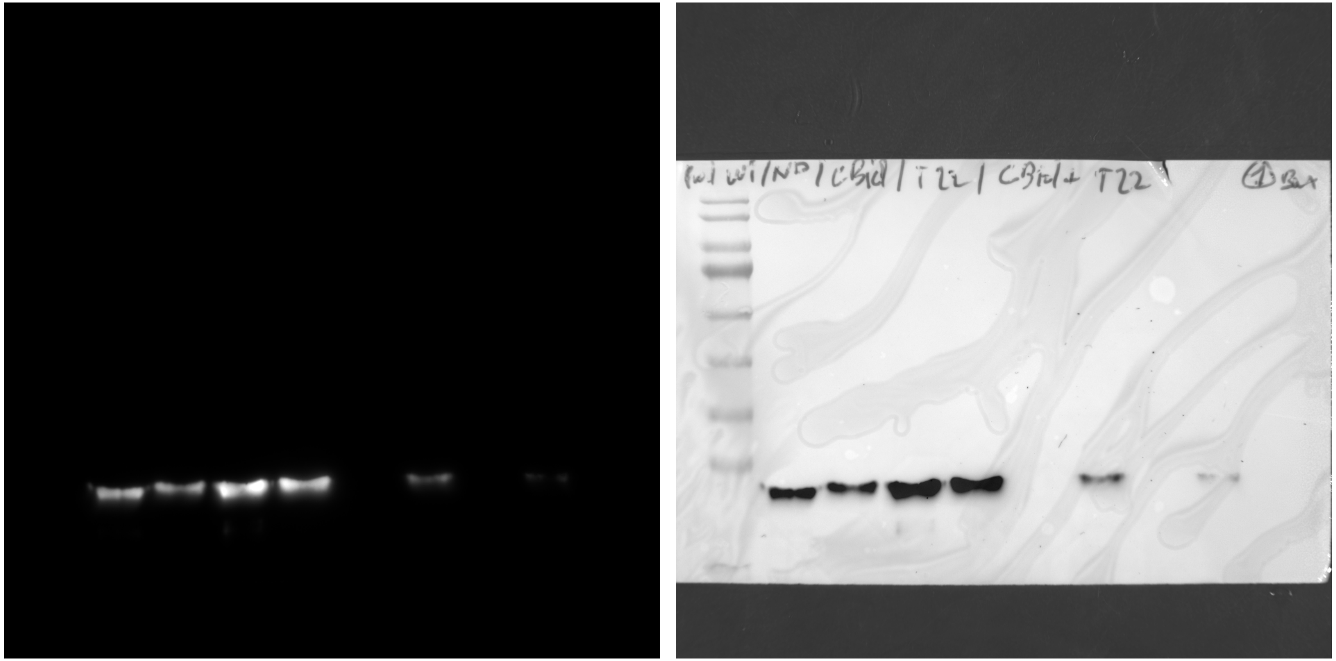

FigS5B_20230214

bax

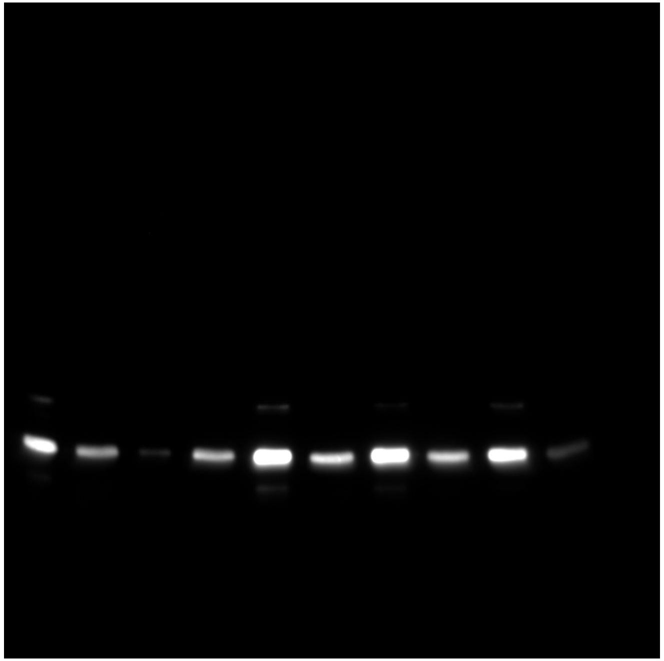

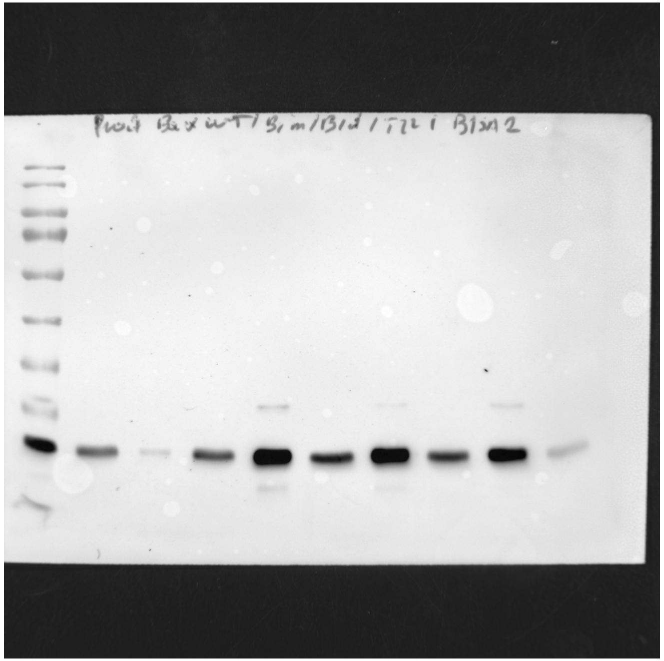

FigS5C_20230217

bax

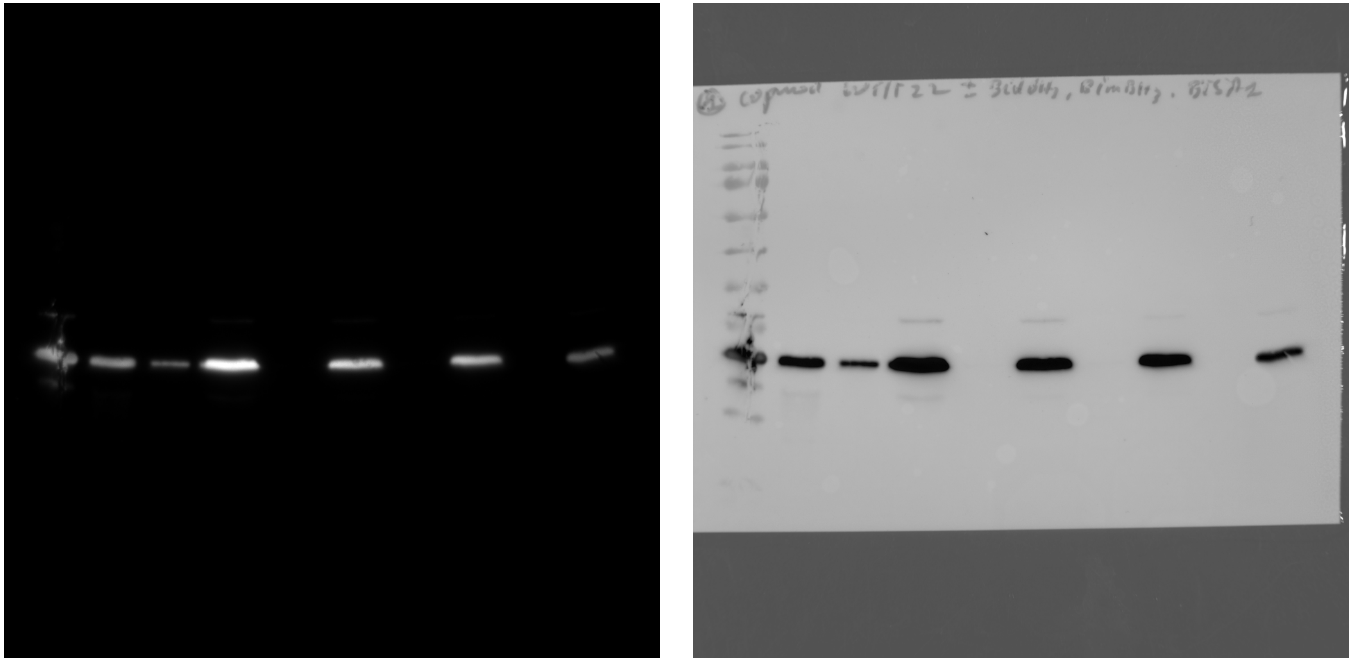

FigS5C_20230217

tom22

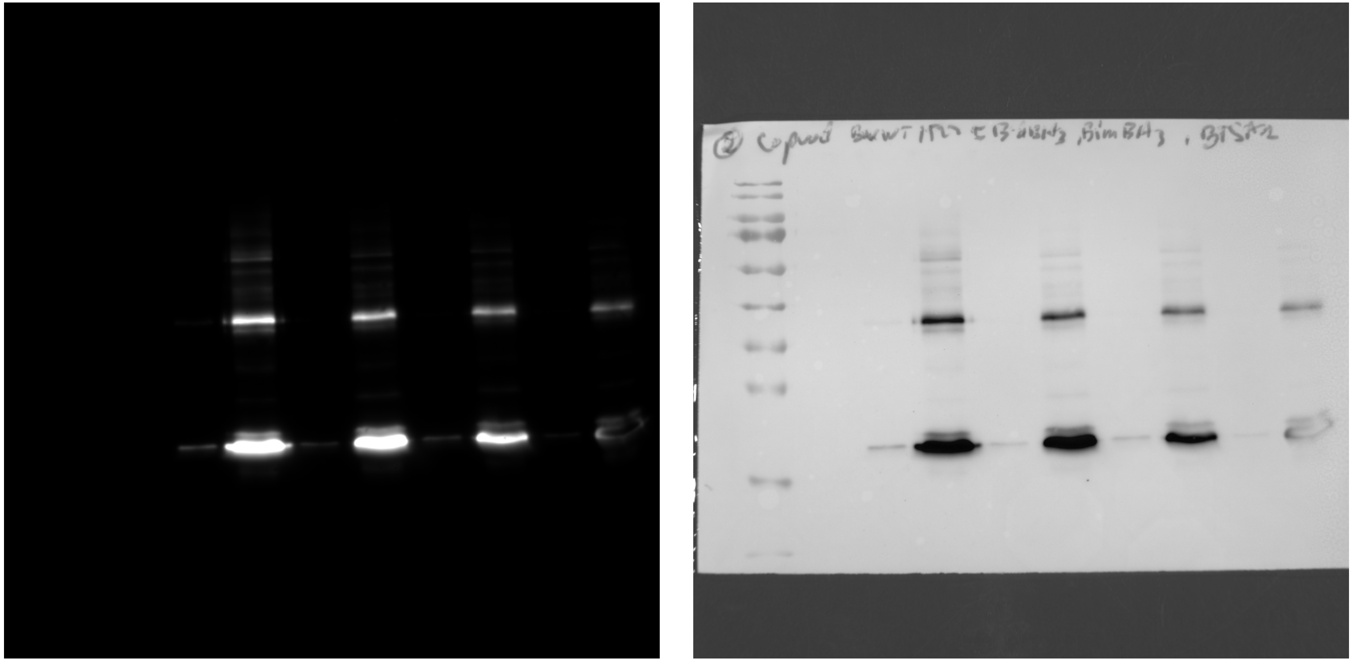

FigS5D_20230220

bax+tom22

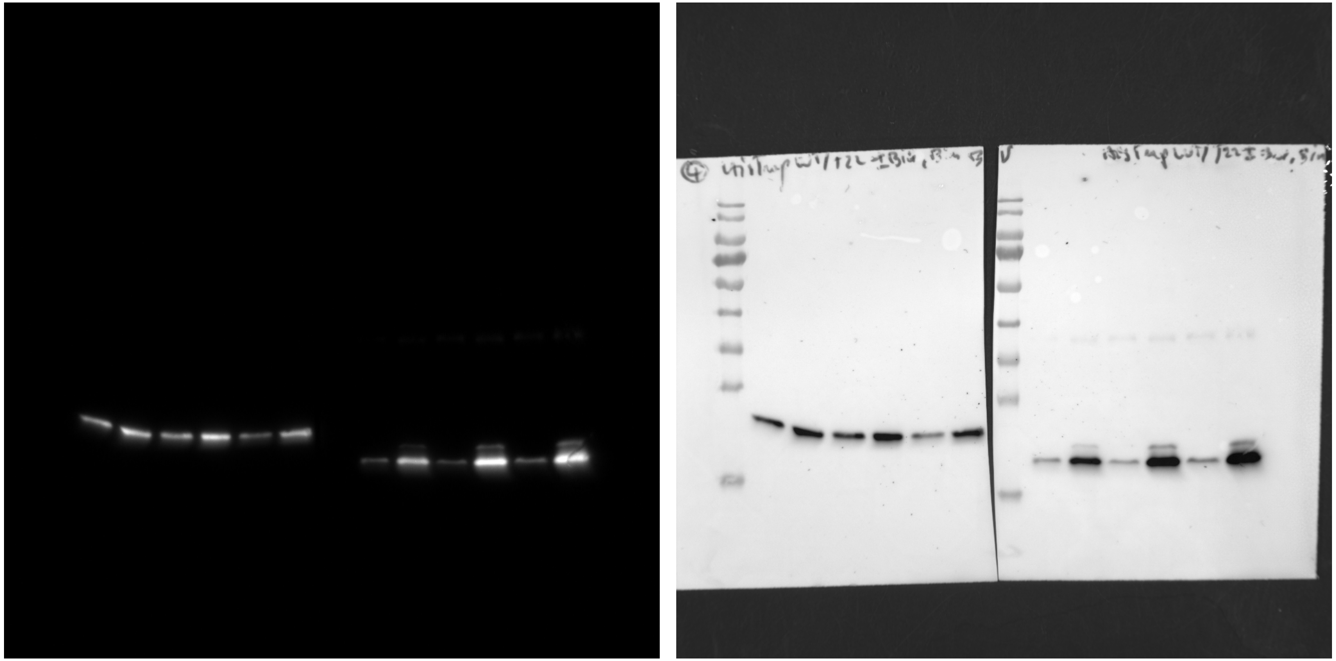

FigS5D_20230220

his6

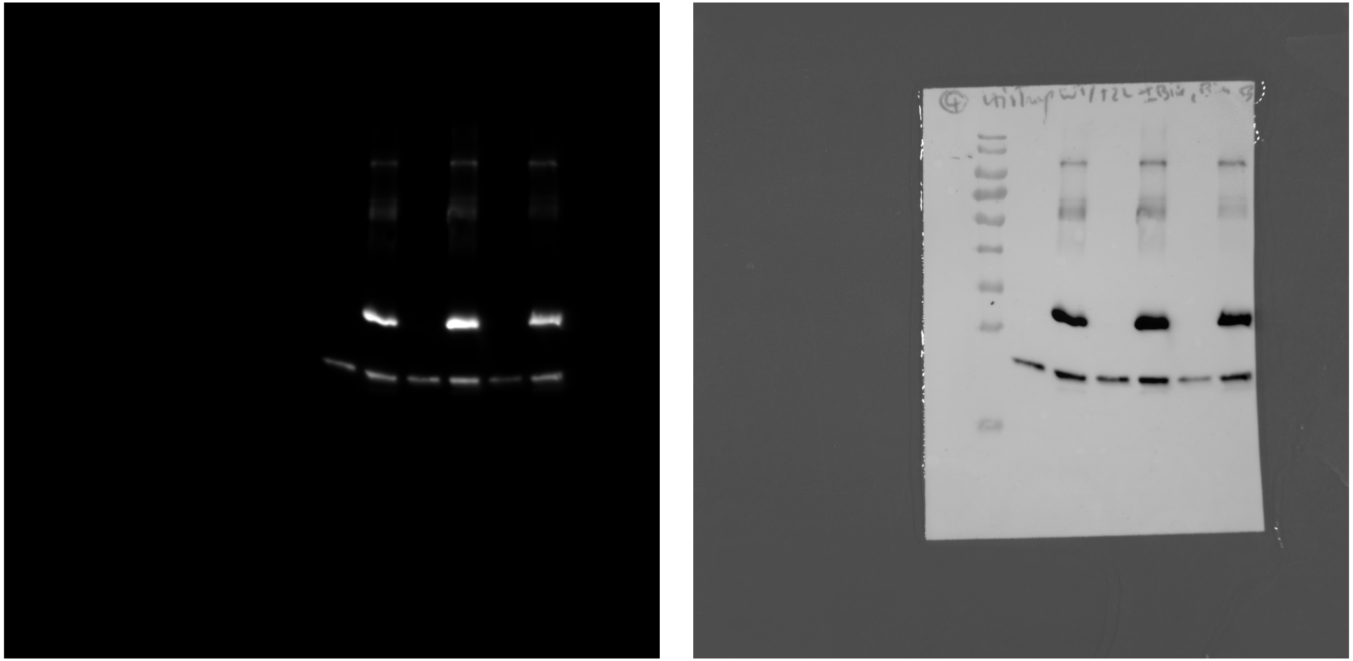

Supplement: Supplementary file 2 — Original Data [file 41420_2024_2108_MOESM2_ESM.pdf]
